# A new face of phenalenyl-based radicals in the transition metal-free C–H arylation of heteroarenes at room temperature: trapping the radical initiator *via* C–C σ-bond formation†
†Electronic supplementary information (ESI) available. CCDC 1520738. For ESI and crystallographic data in CIF or other electronic format see DOI: 10.1039/c7sc02661g



**DOI:** 10.1039/c7sc02661g

**Published:** 2017-09-12

**Authors:** Jasimuddin Ahmed, Sreejyothi P, Gonela Vijaykumar, Anex Jose, Manthan Raj, Swadhin K. Mandal

**Affiliations:** a Department of Chemical Sciences , Indian Institute of Science Education and Research Kolkata , Mohanpur-741246 , Kolkata , India . http://swadhin-mandal.weebly.com/ ; Email: swadhin.mandal@iiserkol.ac.in; b Zakir Husain Delhi College , University of Delhi , Delhi-110002 , India

## Abstract

The radical-mediated transition metal-free approach for the direct C–H bond functionalization of arenes is considered as a cost effective alternative to transition metal-based catalysis. An organic ligand-based radical plays a key role by generating an aryl radical which undergoes a subsequent functionalization process. The design principle of the present study takes advantage of a relatively stable odd alternant hydrocarbon-based phenalenyl (PLY) radical. In this study, the first transition metal-free catalyzed direct C–H arylation of a variety of heteroarenes such as azoles, furan, thiophene and pyridine at room temperature has been reported using a phenalenyl-based radical without employing any photoactivation step. This protocol has been successfully applied to the gram scale synthesis of core moieties of bioactive molecules. The phenalenyl-based radical initiator has been characterized crystallographically by trapping it *via* the formation of a C–C σ-bond between the phenalenyl radical and solvent-based radical species.

## Abstract 2

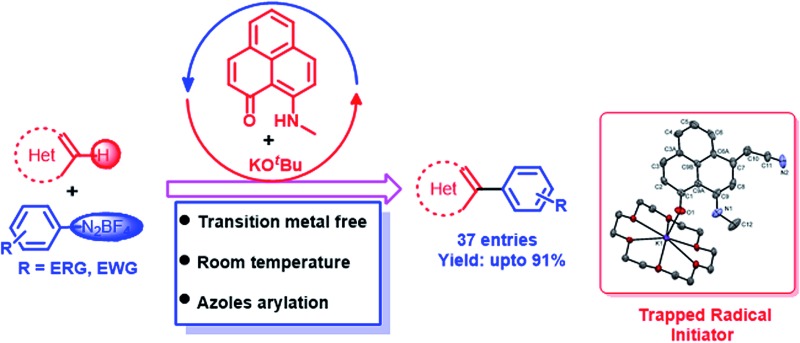
The first transition metal-free catalyzed direct C–H arylation of a variety of heteroarenes at room temperature has been reported using a phenalenyl-based radical without employing any photoactivation step.

## Introduction

**Scheme 1:**
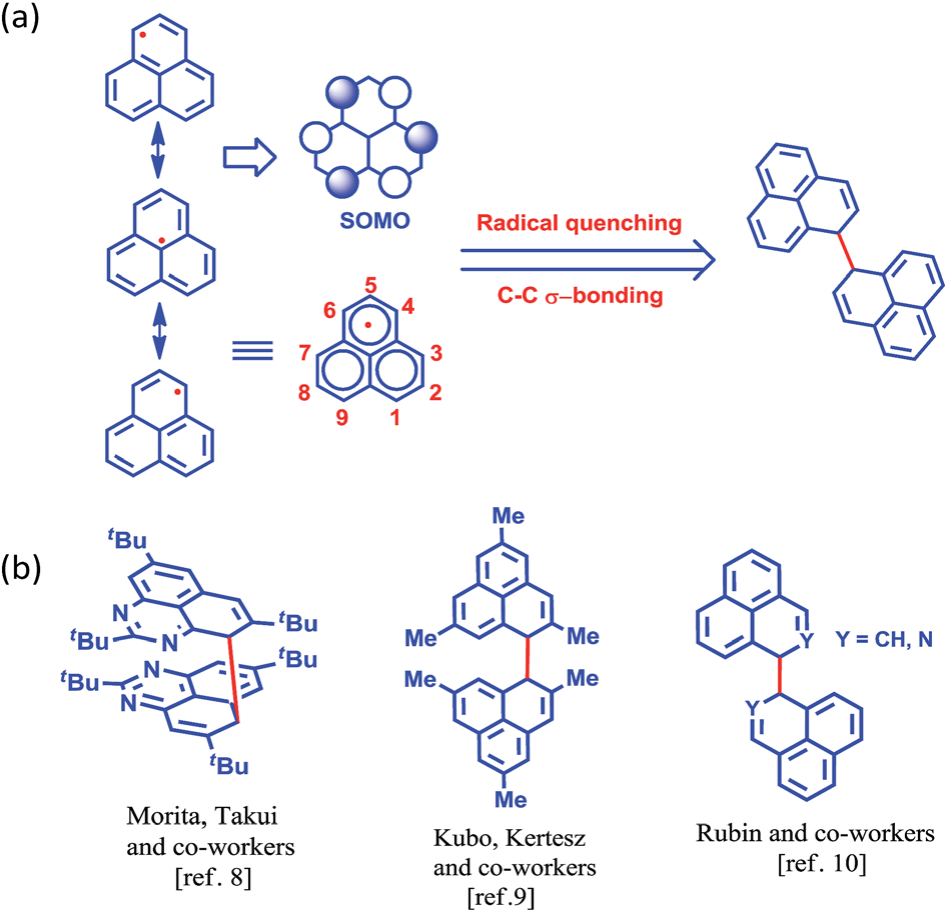
(a) Canonical forms of the phenalenyl radical showing the spin-bearing centers. (b) Examples of phenalenyl radicals that have undergone C–C σ-bond formation.

More than half a century ago,^1,2^ Hückel molecular orbital calculations revealed that the odd alternant hydrocarbon phenalenyl (PLY) has a non-bonding molecular orbital (NBMO)^2^ that can switch between redox active closed-shell (cationic) and open-shell (neutral radical) electronic configurations. Stabilization of the unpaired spin in the neutral radical state of phenalenyl has been utilized over the last two decades for designing various materials with intriguing properties^3–5^ since the pioneering suggestion by Haddon.^6^ The resonance stability of the phenalenyl radical indicates that C1, C3, C4, C6, C7 and C9 (Scheme 1a) are electron rich centers and thus they are termed as spin-bearing positions. Despite the solution characterization of the phenalenyl-based radical, it has been a challenge for a long time to characterize it in the solid state owing to its ready propensity to undergo radical quenching through C–C σ-bond formation (Scheme 1)^7–10^ *via* one of these spin-bearing carbon centers. Nevertheless, the realization of phenalenyl-based radicals in the solid state was accomplished either by chemically blocking the spin-bearing carbon centers or by introducing sterically demanding groups in the periphery of the phenalenyl radical.^5,11–13^ In this way, phenalenyl-based radicals gave rise to a new class of organic radical with promise for various applications such as in optoelectronic and spintronic devices, molecular switches, molecular batteries, quantum spin simulators, *etc.* ^3,8,14–16^

Recently, we used a strategy which avoids the isolation of the phenalenyl radical, and realized that the cationic (closed-shell) phenalenyl moiety with an empty NBMO can be generated by metal ion coordination,^17–19^ and the topic has recently been reviewed.^20^ In the present work, we have utilized K(I) ion-coordinated phenalenyl, which can accept an electron from a donor to generate the phenalenyl-based radical to undergo the facile C–H functionalization of different heteroarenes under mild conditions. In recent years, direct catalytic C–H functionalization has emerged as a powerful synthetic platform for the step-economical synthesis of bi-aryls.^21^ In this regard, the transition metal-free base-promoted homolytic aromatic substitution (BHAS) of arenes with aryl halides in the presence of a base has drawn considerable attention as an alternative strategy to the intensively investigated transition metal-catalyzed C–H activation.^22^ Seminal research efforts by the groups of Itami,^23^ Shi,^24^ Shirakawa, Hayashi,^25^ Lei^26^ and Murphy^27^ reported the diversity and scope of this newly developed method in transition metal-free arylation reactions. Previously, we reported the BHAS reaction of benzene derivatives using aryl iodide in the presence of a catalytic amount of phenalenyl ligand at an elevated temperature (130 °C).^28^ Despite this progress, BHAS in general faces major disadvantages, such as: (1) the use of rather extreme reaction conditions, with a typical reaction temperature ranging from 100 to 160 °C, (2) the very high loading of base, KO^*t*^Bu (2–3 equiv.), and organic ligand (typically 20–40 mol% loading), (3) the use of a very high number of equivalents of arene partners (50–100 equiv.) and (4) the fact that it is not useful for the arylation of heteroarenes, particularly at room temperature.

The transition metal-free direct arylation of heteroarenes under ambient conditions (at room temperature without light stimulation) is extremely rare in the literature.^29^ A notable effort by König^30^ reported the direct C–H arylation of heteroarenes under metal-free conditions at room temperature using aryl diazonium salt as a coupling partner. Very recently, Lee and coworkers reported aryl diazonium as a coupling partner for biaryl synthesis using rare metal-based catalysts (Ru and Au).^31^ However, all of these catalytic methods required stimulation by light, and the scope of the reaction was not tested for the C–H arylation of azoles. This is considered as an important objective, which was very recently accomplished by Ackermann^32^ at room temperature using a transition metal-based catalyst (Cu) and light. We herein report transition metal-free catalysis at room temperature without photocatalytic activation for the direct C–H arylation of heteroarenes, including azoles, using aryl diazonium salt as a coupling partner. A phenalenyl (PLY) ligand has been selected for this catalysis, as PLY is well-known to stabilize the radical state due to the presence of a nonbonding molecular orbital (NBMO).^17^ The notable achievements of our current catalytic protocol are: (1) the successful transition metal-free direct C–H arylation of heteroarenes including azole substrates, (2) neither heating nor light irradiation is required and (3) the method provides easy access to medicinally important structural motifs.

**Scheme 2:**
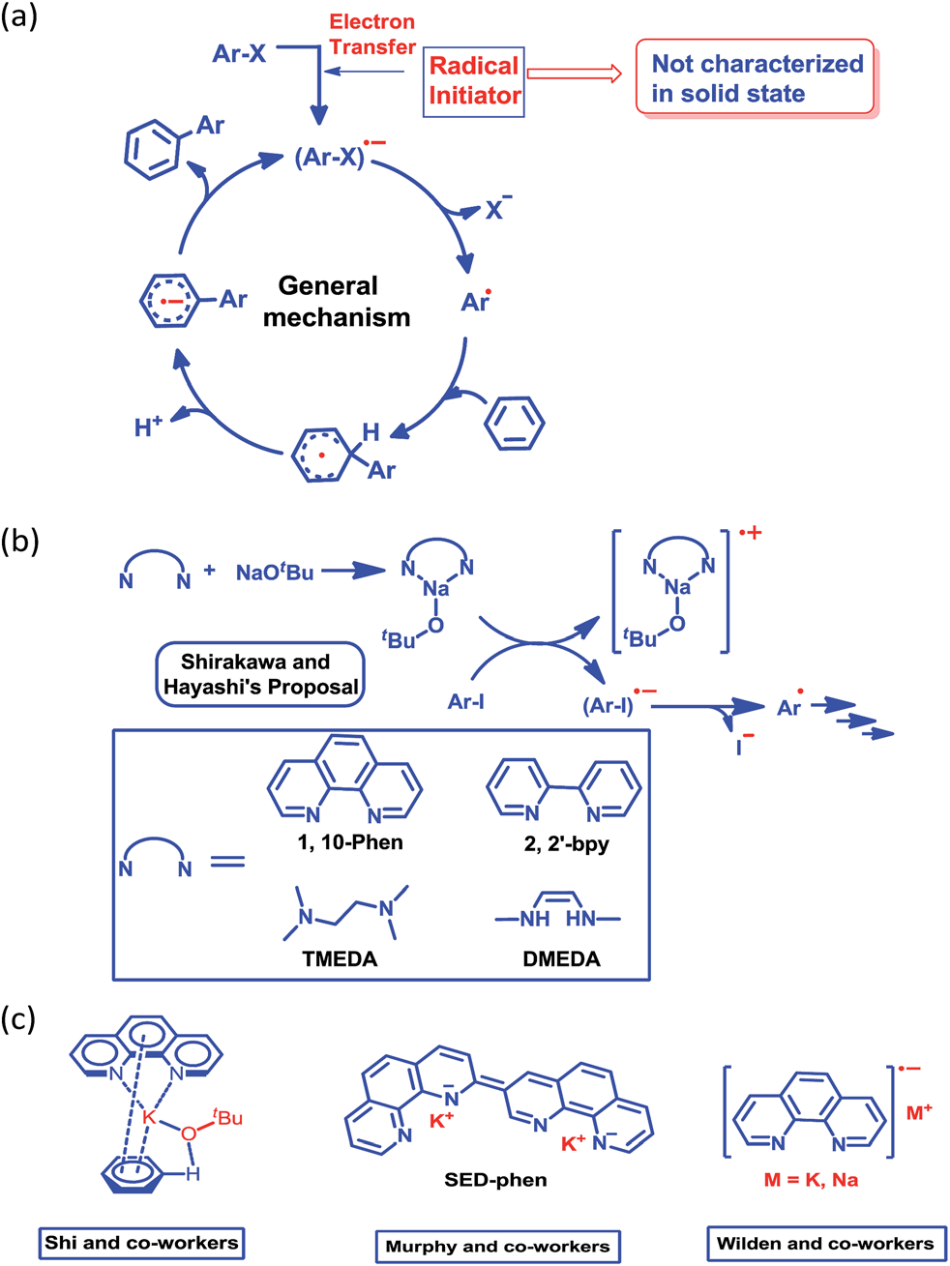
(a) General mechanism for the radical-mediated C–H functionalization process. (b) Shirakawa and Hayashi’s proposal of an organic ligand metal complex-based initiation process. (c) The proposed key intermediates for this reaction.

Although nearly a decade has passed since transition metal-free catalytic arylation was reported,^23^ a complete understanding of the mechanistic process supported by a solid state structure is missing in current literature. It is now generally accepted that the reaction works through a radical-mediated single electron transfer (SET) process, generating a highly reactive aryl radical species (Scheme 2a).^33^ Shirakawa, Hayashi and co-workers have proposed that an externally added organic ligand along with a base may generate a radical initiator, which can subsequently inject an electron into the aryl substrate (Scheme 2b).^25^

This proposal was further reinforced by the work of Wilden and co-workers.^34^ Later on, Murphy and co-workers proposed an alternative concept of a super electron donor (SED) which initiates the radical reaction (Scheme 2c).^35*a*^ Despite several proposals, there remains an open question about the radical initiator as it has never been trapped. Shi and coworkers proposed a structure of the intermediate (Scheme 2c) considering the phenanthroline, base and substrate, however they failed to document any solid state structural evidence.^24^ In this study, we were able to trap and structurally characterize the elusive organic ligand-based radical initiator which plays the key role in the generation of the highly reactive aryl radical.

## Results and discussion

We optimized the catalytic C–H arylation reaction using thiazole (**1**) as the heteroarene partner and **2a** as the aryl diazonium coupling partner to obtain the product **3a** through arylation at the C2 position of thiazole (Table 1) under different conditions using PLY-based ligands.

**Table 1:** Reaction optimization for the transition metal-free C–H arylation of thiazole (**1**) at room temperature^*a*^

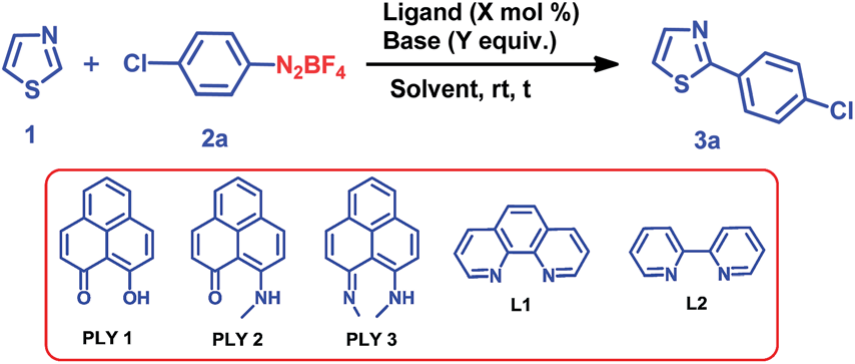					
Entry	Ligand (mol%)	Base (equiv.)	Solvent	Time (h)	Yield^*b*^ (%)
1	PLY1 (5)	KO^*t*^Bu (1)	DMSO	24	28
2	PLY2 (5)	KO^*t*^Bu (1)	DMSO	24	67
3	PLY3 (5)	KO^*t*^Bu (1)	DMSO	24	59
4	PLY2 (5)	KO^*t*^Bu (1)	DMF	24	37
5	PLY2 (5)	KO^*t*^Bu (1)	THF	24	0
6	PLY2 (5)	NaO^*t*^Bu (1)	DMSO	24	34
7	PLY2 (5)	KO^*t*^Bu (0.1)	DMSO	24	64
8	PLY2 (5)	KO^*t*^Bu (0.1)	DMSO	12	46
9	PLY2 (1)	KO^*t*^Bu (0.1)	DMSO	24	32
10	PLY2 (3)	KO^*t*^Bu (0.1)	DMSO	24	49
11^*c*^	PLY2 (5)	KO^*t*^Bu (0.1)	DMSO	24	62
12	None	KO^*t*^Bu (0.1)	DMSO	24	<10
13	PLY2 (5)	None	DMSO	24	0
14	**L1**	KO^*t*^Bu (0.1)	DMSO	24	<10
15	**L2**	KO^*t*^Bu (0.1)	DMSO	24	<10

^*a*^Reaction conditions: thiazole (0.72 mmol), **2a** (0.24 mmol), ligand (5 mol%, 0.012 mmol/1 mol%, 0.0024 mmol/3 mol%, 0.0072 mmol) and KO*^t^*Bu/NaO*^t^*Bu (1 equiv., 0.24 mmol/10 mol%, 0.024 mmol).

^*b*^Isolated yield.

^*c*^Dark conditions.

We tested three different PLY ligands, PLY1, PLY2 and PLY3 (Table 1), with 5 mol% loading in the presence of 1 equiv. of KO^*t*^Bu (w.r.t. the diazo coupling partner) in DMSO for 24 h at ambient temperature. PLY2 displayed the best efficacy (67% yield) over the other two PLY ligands (PLY1: 28% yield and PLY3: 59% yield, Table 1, entries 1–3). When the same reaction was performed with 10 mol% of KO^*t*^Bu, the yield of product **3a** remained the same as that with 1 equiv. of base (Table 1, entry 7). Low catalyst loading experiments were performed using 1 and 3 mol% PLY2, which resulted in 32% and 49% yields, respectively (Table 1, entries 9 and 10). To investigate the effect of light on this reaction (if any), the reaction was performed under completely dark conditions, affording 62% yield of the product (Table 1, entry 11), which clearly indicates that there is no role of light in this reaction protocol. When the same reaction was performed with 10 mol% of only KO^*t*^Bu (in the absence of any PLY ligand), keeping other conditions identical (as in entry 2 of Table 1), the yield of product **3a** was drastically reduced to below 10% (entry 12, Table 1). This result clearly points out that the presence of a PLY ligand in a catalytic amount is essential for this reaction to enhance the yield up to 64% (entry 7, Table 1). Furthermore, under these optimized conditions, we also tested two well-known organic ligands frequently used for base-promoted direct C–H arylation, 1,10-phenanthroline (**L1**) and 2,2′-bipyridine (**L2**). Both ligands afforded a yield of **3a** below 10% (Table 1, entries 14 and 15). This observation establishes that PLY-based ligands are superior to these organic ligands, which have been typically used in semi-stoichiometric loading conditions (20–40 mol%). Finally, the direct C–H arylation of thiazole (**1**) with aryl diazonium salt **2a** in the presence of 5 mol% PLY2 and 10 mol% KO*^t^*Bu in DMSO for 24 h at room temperature (Table 1, entry 7) may be considered as the most attractive condition among all tested conditions (Table 1).

**Fig. 1:**
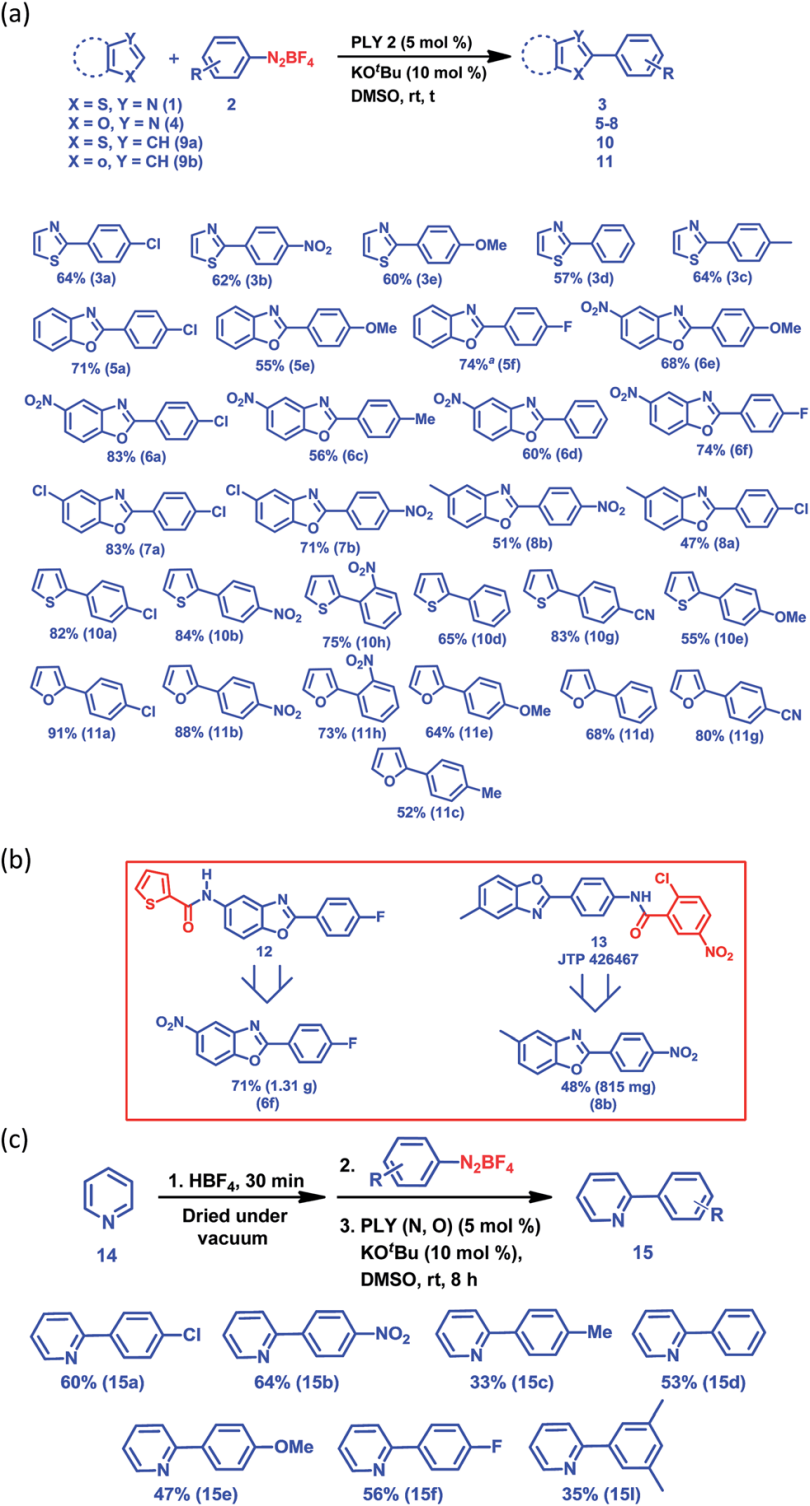
The transition metal-free C–H arylation of heteroarenes at room temperature. (a) The substrate scope for heteroarene arylation. (b) Large-scale synthesis of the core parts of bioactive molecules. (c) The C2 arylation of pyridine. ^*a*^NMR conversion: *t* = 24 h for azole arylation and *t* = 8 h for furan and thiophene arylation.

Next, we examined the applicability of our catalytic protocol towards different azole and aryl diazonium salt coupling partners. At first, our protocol was successfully applied to the arylation of thiazole (**1**) with five different aryl diazonium salt coupling partners, affording 57–64% (Fig. 1a) yields of the C2-arylated products of thiazole (**3a–3e**). The yields of the arylated products for the activated aryl coupling partners (**3a** and **3b**) and un-activated aryl coupling partners (**3c–3e**) are similar. Itami and co-workers reported a Pd and Ni-catalyzed C2 selective arylation of simple thiazole for the programmed synthesis of tri-arylated thiazoles, the yield of the corresponding product using a Ni-based catalytic protocol at 120 °C is comparable with our yield (for **3d**).^36^ Furthermore, the arylation of five different benzoxazoles was successfully carried out with different aryl diazonium coupling partners containing both electron-withdrawing and electron-donating groups (Fig. 1a). Here, 6-nitrobenzoxazole (**4b**) and 6-chlorobenzoxazole (**4c**) resulted in excellent yields of the corresponding C2-arylated products (up to 83%) compared to the arylation yield of simple benzoxazole (**4a**) and 6-methylbenzoxazole (**4e**) (up to 71%). Furthermore, it is noteworthy that the present protocol was also successfully applied for the C–H arylation of heteroarenes such as thiophene (**9a**) and furan (**9b**) at room temperature, resulting in good to excellent yields (13 examples, 52–91% yields of **10a–11h**, Fig. 1a). The direct C–H arylation of heteroarenes (mainly furan and thiophene) under metal-free conditions at room temperature using aryl diazonium salt as a coupling partner has been reported by König and coworkers,^30^ but it required light stimulation. Carrillo and coworkers^29^ reported the metal-free arylation of furan and thiophene at room temperature only with activated diazonium coupling partners. This synthetic strategy has the potential to build up an efficient and common platform for the arylation of biologically active azoles at room temperature under transition metal-free conditions. It may be noted that different functional groups, such as halogens, nitro groups and nitriles, are compatible with our present C–H arylation protocol, extending the scope of this organic transformation. We utilized this protocol for the large-scale synthesis of **6f** and **8b**, which are core moieties of two different biologically active molecules: an antimicrobial agent (**12**)^37^ and the selective PPAR antagonist JTP 426467 (**13**),^38^ respectively (Fig. 1b). The successful synthesis of these core parts of bioactive molecules at room temperature indeed shows the unique applicability of our C–H arylation approach under cost-effective transition metal-free conditions.

Next, we started an investigation into the C2 arylation of pyridine (**14**) which mainly relies on transition metal catalysis.^39^ Very recently, the TiO_2_-catalyzed C2 arylation of pyridine has been carried out in a flow reactor in the presence of blue light with moderate to excellent yield.^40^ At first, the arylation of pyridine was attempted following the method developed for the arylation of furan and thiophene, however it did not result in the formation of any pyridine-arylated product. Finally, an alternative strategy was used to make the C2 position of pyridine more electrophilic by forming the pyridyl salt *in situ* on addition of an acid (HBF_4_). The pyridine arylation was carried out with various aryl diazonium salt coupling partners and the results are presented in Fig. 1c (also see the ESI,† pages 5 and 6). Using this methodology, the arylation of pyridine (**14**) was carried out with seven different aryl diazonium salt coupling partners (**2a–f** and **2I**) to yield the C2-arylated products (**15a–f** and **15I**) in moderate to good yields (33–64%) (Fig. 1c) under transition metal-free conditions at room temperature. However, our method did not succeed for the arylation of simple arenes such as benzene when attempted with aryl diazonium salt as the coupling partner at room temperature.

**Fig. 2:**
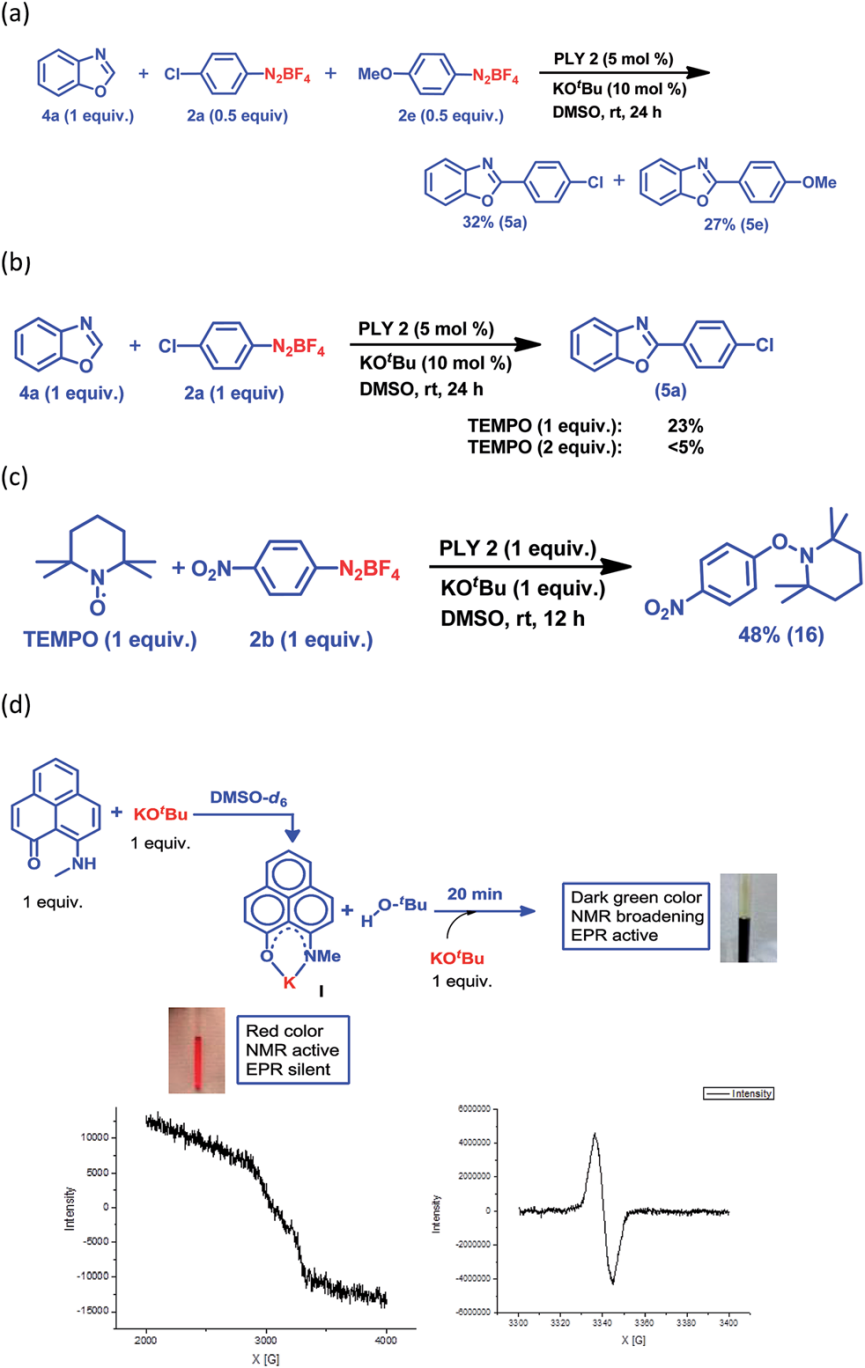
Mechanistic investigations. (a) The competition reaction between benzoxazole and a mixture of two aryl diazonium salts, **2a** and **2e**. (b) Inhibition of the benzoxazole arylation reaction in the presence of TEMPO. (c) Trapping of the aryl radical by TEMPO. (d) The stoichiometric reaction between a phenalenyl-based ligand and KO^*t*^Bu along with EPR spectra of complex **I** (before reduction) and green colored species (after reduction).

Keeping this versatile and efficient C–H arylation of different heteroarenes in hand, we started a detailed investigation into the mechanistic pathway. At first, an intermolecular competition experiment between benzoxazole and a mixture of two different aryl diazonium coupling partners, one of which had an electron-donating substituent (**2e**) and the other had an electron-withdrawing substituent (**2a**), afforded **5e** (27%) and **5a** (32%) respectively (Fig. 2a), suggesting that there was no bias from the electronic effect of the substituents. This result strongly indicates that a radical intermediate is involved in the catalytic cycle.^25,28^ Next, the reaction between benzoxazole (**4a**) and the aryl diazonium salt coupling partner (**2a**) was carried out in the presence of a well-known radical scavenger, 2,2,6,6-tetramethylpiperidinoxyl (TEMPO), under different loading conditions (Fig. 2b). In the presence of 1 equiv. of TEMPO, only 23% of the arylated product was obtained, whereas in the presence of 2 equiv. of TEMPO, the reaction barely proceeded at all.

Furthermore, a reaction between TEMPO and the aryl diazonium salt coupling partner **2b** was performed with our optimized conditions but in the absence of any heteroarene partner, which resulted in the formation of a TEMPO-trapped aryl radical intermediate, **16**, with 48% yield (Fig. 2c). These results clearly suggest that a radical-mediated SET process is involved, which has been well-accepted for radical-assisted direct C–H arylation.^23–27^ To understand this process of aryl radical generation, we attempted to trap the elusive organic ligand-based radical (in this case, the phenalenyl-based radical), which was proposed as an initiator for aryl radical generation nearly a decade ago.^24^

**Fig. 3:**
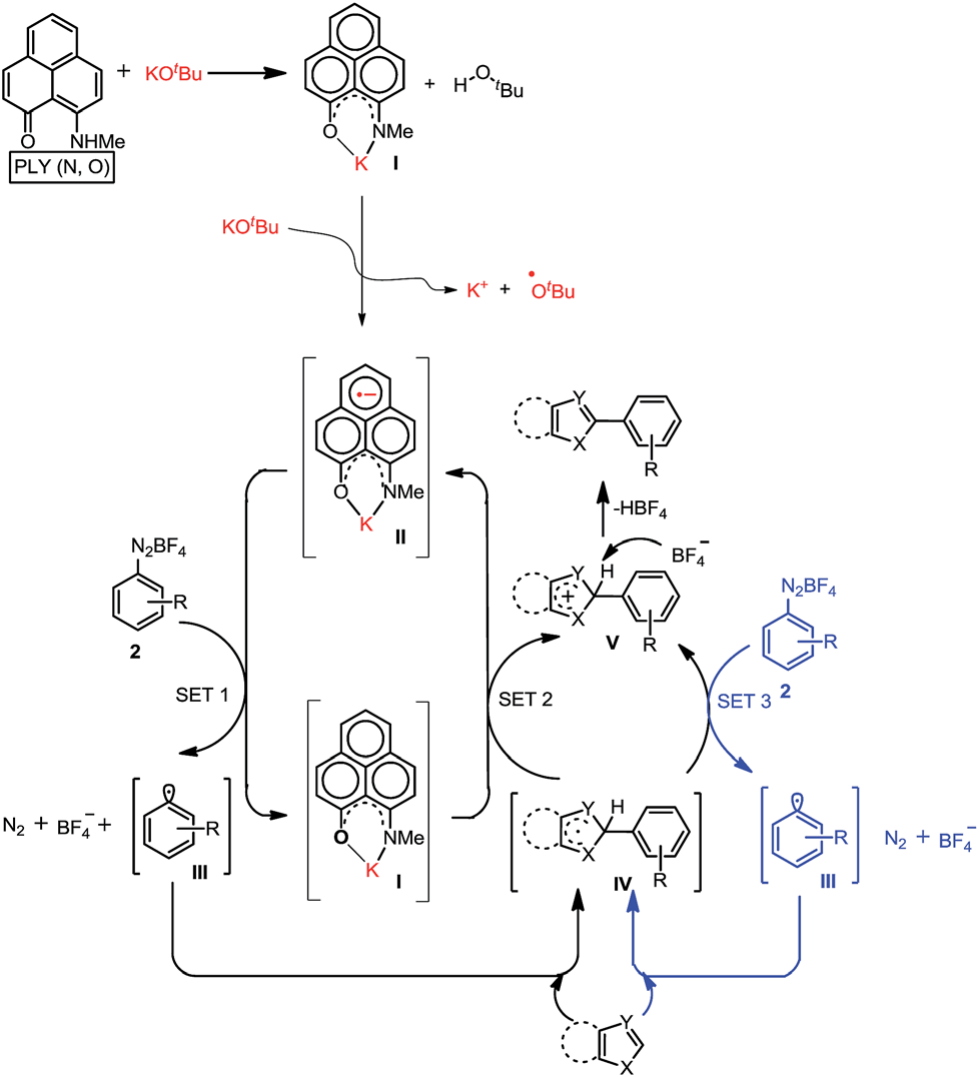
The plausible mechanistic reaction pathway.

At first, we attempted to synthesize the K(I) ion-coordinated phenalenyl complex by performing the stoichiometric reaction strictly under dry and inert conditions. Upon addition of yellow-colored PLY2 and KO^*t*^Bu (1 : 1) in DMSO-*d*_6_ solvent (see the ESI†), the reaction mixture immediately turned red (Fig. 2d). The ^1^H NMR spectrum of the red solution in DMSO-*d*_6_ indicated the formation of a K-PLY complex, **I**, which resulted in the upfield shift of peaks in the ^1^H NMR spectrum compared to those in the ^1^H NMR spectrum of the free PLY2 ligand in the same solvent (see the ESI, Fig. S3†). The absence of the N–H proton (present in free PLY2) in the ^1^H NMR spectrum (see the ESI, Fig. S3†) near *δ* 12 ppm clearly suggests abstraction of the N–H proton from the PLY2 ligand and formation of a PLY coordinated K(I) complex **I**. Furthermore, the ^1^H NMR spectrum of the red solution showed the presence of free *tert*-butanol (*δ* 1.11 ppm), which confirms the deprotonation of the PLY2 ligand. Immediate EPR measurement of this red solution displayed no EPR signal, establishing the reaction mixture as EPR-silent. On addition of another equiv. of KO^*t*^Bu to the solution, the red solution undergoes a sharp color change to deep green within 20 min (Fig. 2d). The EPR measurement of this green reaction mixture reveals a strong EPR signal, indicating the generation of a radical species (*g* = 2.00187, Fig. 2d). On the basis of the above observations and the literature reports,^24–26^ a plausible mechanism is proposed in Fig. 3. Herein, complex **I** is generated *in situ* by the abstraction of the N–H proton from PLY2 in the presence of KO^*t*^Bu. The PLY ligand, upon coordinating with the metal ion (K^+^), generates the closed shell cationic state of the PLY moiety with an empty NBMO, as observed in our earlier studies.^18,19^ This empty NBMO can readily interact with another equiv. of KO^*t*^Bu, which transfers an electron resulting in the formation of an active PLY-based radical anion, **II**, and an O^*t*^Bu radical (*vide infra*). The role of KO*^t^*Bu in transferring electron is well documented in the literature.^34,35b,c^ The earlier studies revealed that the generated *tert*-butoxide radical undergoes *in situ* decomposition.^35d,e^ Subsequently, the singly occupied molecular orbital (SOMO) of the phenalenyl-based radical anion **II** can transfer the electron (*via* SET) to the aryl diazonium salt, forming a very reactive aryl radical, **III**.^30^ During this process, the radical anion **II** converts into complex **I**. This proposition is further reinforced by Shirakawa and Hayashi’s^24,25^ mechanistic outline, where SET occurs from a formal NaO^*t*^Bu-phenanthroline complex to the corresponding aryl iodide (Scheme 2b), generating an aryl radical. The aryl radical **III** reacts with the heteroarene at the more electrophilic C2 position, forming a radical transition state, **IV**. Subsequently, **IV** undergoes another SET process to regenerate the active catalyst **II** from complex **I** and transforms into a cationic intermediate, **V**. Finally, proton abstraction from **V** by BF_4_^–^ leads to the C2-arylated product.^30^

**Fig. 4:**
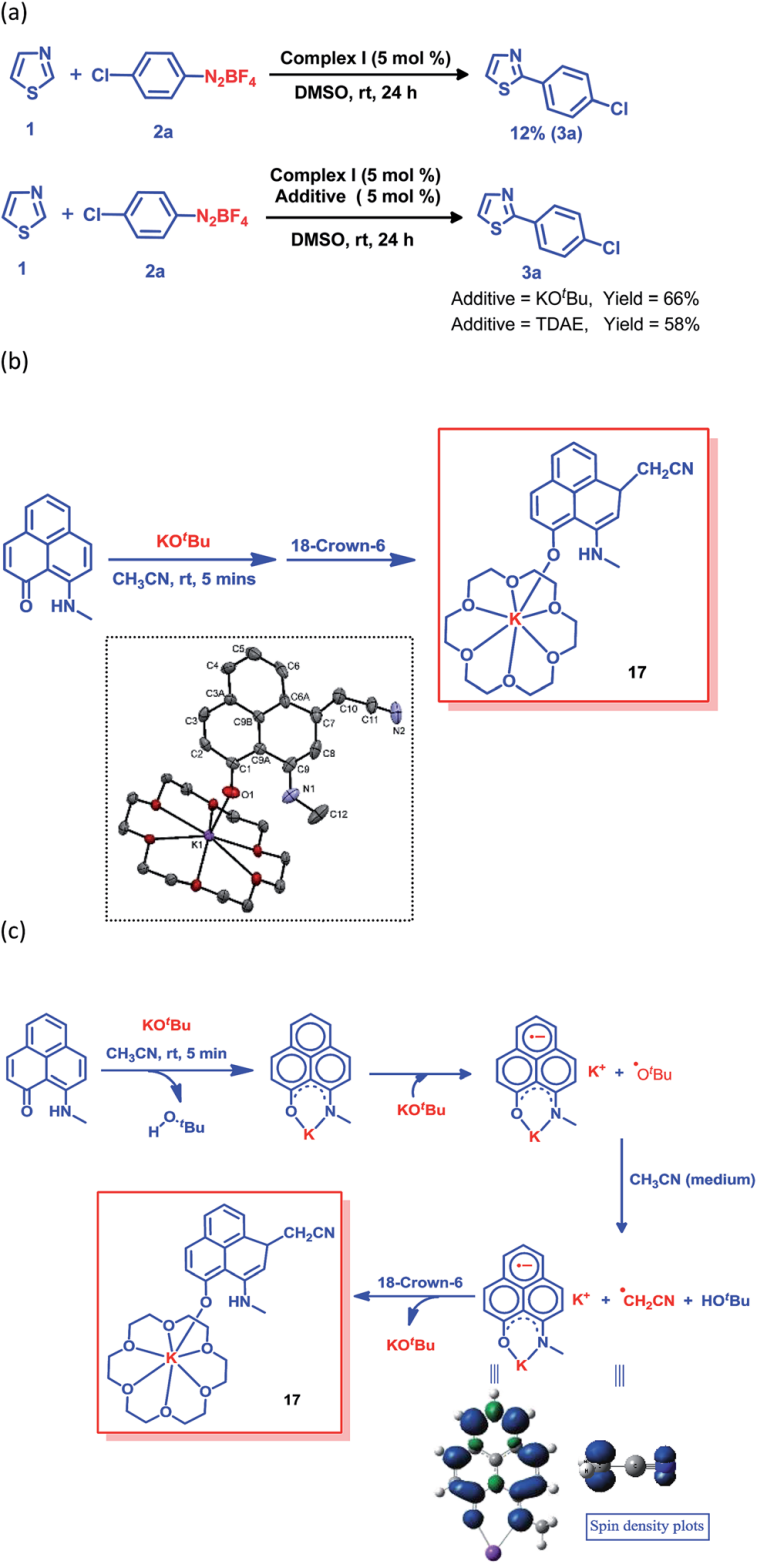
The radical trapping reaction in acetonitrile. (a) The effect of an electron donor (KO^*t*^Bu or TDAE) on the yield. (b) Formation of the trapped phenalenyl radical and its solid state structure, and perspective ORTEP views of the molecular structure of **17**. Thermal ellipsoids are drawn with 50% probability. Hydrogen atoms and solvent molecules (acetonitrile) have been omitted for the sake of clarity. Selected bond lengths (Å) and bond angles (°) for the structure of complex **17**: O1–K1 2.570(4), O1–C1 1.300(6), C10–C11 1.397(8), C7–C10 1.602(8) and N2–C11 1.128(7); K1–O1–C1 139.7(3), C6A–C7–C8 112.8(4), C9–C8–C7 123.3(5), C11–C10–C7 111.5(5) and N2–C11–C10 173.9(7). (c) The reaction pathway for trapping the phenalenyl-based radical complex and spin density plots of the K-PLY2 radical complex and CH_2_CN radical.

The generation of the aryl radical is the basis for all transition metal-free radical-based coupling catalysis reactions. Since the first report on a transition metal-free radical-based coupling method, over the last decade, an organic ligand-based radical initiator has been postulated but it has never been trapped or realized in the solid state.^24,25,28^ To understand this radical initiator generation process, we first set up a few control experiments using KO^*t*^Bu, as well as using a well-known organic electron donor, tetrakis(dimethylamino)ethylene (TDAE). At first, the K complex of PLY2 was prepared in an alternative way by adding PLY2 and KO^*t*^Bu (1 : 1) in benzene with stirring at room temperature for 10 min until a red precipitate was formed. The reaction mixture was then completely dried under reduced pressure to make sure there was no residual butanol present. It was characterized by ^1^H NMR spectroscopy, and the spectrum was very similar to the ^1^H NMR spectrum of the K-PLY2 complex (**I**) prepared in DMSO-*d*_6_ (see the ESI, Fig. S3†). A catalytic reaction between 4-chlorophenyl diazonium salt (**2a**) and thiazole (**1**) in the presence of 5 mol% K-PLY2 complex (the red solid obtained from benzene which was free of any *tert*-butanol) in DMSO resulted in only 12% C2-arylated product formation (Fig. 4a). The addition of KO^*t*^Bu (5 mol%) results in a dramatic improvement of the yield to 66%. We observed a similar yield enhancement to 58% when we used a purely organic electron donor, TDAE (Fig. 4a). This result indicates that KO^*t*^Bu or TDAE acts as an electron donor to generate the PLY-based radical which subsequently undergoes a SET process to complete the catalytic cycle. Moreover, the addition of external KO^*t*^Bu or TDAE to the K-PLY2 complex resulted in a sharp change in color from red to deep green within 15–20 min, suggesting phenalenyl-based radical generation (also see Fig. 2d above). It may be concluded that the initially formed red K-PLY complex becomes a dark green-colored radical species after taking an electron from KO^*t*^Bu or TDAE. A similar observation was noted with thiophene as the heteroarene partner (see the ESI, page S11†). Next, we were able to trap the elusive radical-based SET initiator from the reaction between the PLY2 ligand and KO^*t*^Bu at room temperature when stabilized with 18-crown-6 ether in acetonitrile solvent. The stoichiometric reaction between the PLY2 ligand and KO^*t*^Bu (1 : 2) in CH_3_CN solvent was performed inside a nitrogen-filled glovebox and the reaction mixture was kept inside the glovebox for crystallization at –20 °C, and dark colored single crystals of compound **17** were obtained after standing overnight (Fig. 4b). The crystals were mounted on a diffractometer while strictly maintaining the crystal temperature below 0 °C with the help of a steady flow of liquid nitrogen. The extreme sensitivity of the crystals was noted as they were unstable over 20 °C, even inside the glovebox. The dark crystals immediately melted into a yellow liquid upon exposure to the atmosphere. To our surprise, the single crystal X-ray structure confirmed the structure of **17**, as shown in Fig. 4b.

The formation of **17** may be explained in a straightforward way by considering a simple base-assisted anion generation (nucleophile) from the acetonitrile solvent followed by recombination of the nucleophile and electrophile. However, in this case, the recombination should occur at the most electrophilic site in the PLY ring (*i.e.* at the *ortho* position of the electronegative O atom of the PLY ligand, C2, which is a non-spin-bearing centre). More logically, the formation of **17** may be rationalized as a quenched state of the phenalenyl radical by forming a C–C σ-bond between the phenalenyl-based radical (*via* the spin-bearing centre) and the CH_2_CN radical formed *in situ*, which is reminiscent of the well-known dimerization process between two radicals *via* C–C σ-bond formation (between two spin-bearing centres) as observed earlier in a number of studies in phenalenyl chemistry (Scheme 1). The C–C σ-bond formation at one of the remote spin-bearing centres far from the electronegative oxygen atom, rather than at one of the more electrophilic non-spin-bearing centres (closer to the oxygen atom), perhaps precludes the possibility of simple nucleophilic addition to the PLY-K complex. The formation of compound **17** may thus be rationalized by considering the following steps, as described in Fig. 4c. In the stoichiometric reaction, firstly the K-PLY complex (**I**) was formed, as described in Fig. 2, which can accept an electron from another equiv. of KO^*t*^Bu. Upon transfer of a single electron, KO^*t*^Bu becomes a butoxide radical which can abstract a proton from CH_3_CN, resulting in the formation of a CH_2_CN radical. The existence of the CH_2_CN radical has been described in earlier reports.^41^ Very recently, CH_2_CN radical formation was reported by Dong and co-workers upon reaction between a methyl radical (generated *in situ* from a *tert*-butoxide radical) and MeCN.^42^ Our theoretical calculations using the B3LYP/6-311g(d) level of theory^43^ reveal that the Δ*G* value is –5.586 kcal mol^–1^ for proton abstraction from acetonitrile by a butoxide radical (Fig. 4c). The spin density calculations support the notion that most of the spin resides over the carbon atom of the CH_2_CN radical. Thus, the CH_2_CN radical can subsequently undergo C–C σ-bond formation with the phenalenyl-based radical in one of its spin-bearing centres, as shown by the calculated spin density representation (Fig. 4c). The H^+^ from butanol can subsequently protonate the nitrogen of the K ion-coordinated PLY2 ligand, generating compound **17** in a concerted manner, in which the K ion is stabilized by coordination with one oxygen from the PLY2 ligand and the oxygen atoms of the crown ether. The X-ray structure reveals a C–C bond distance of 1.616 Å, which is comparable to those observed in previously reported C–C σ-bonded dimeric phenalenyl radicals.^7^ The isolation of **17** is direct solid state evidence showcasing that single electron transfer from KO^*t*^Bu to an organic ligand (in this case the phenalenyl ligand) is a rational step towards realizing the radical initiator which can subsequently undergo a SET process to generate the reactive aryl radical.

## Conclusions

In conclusion, we have developed for the first time the direct C–H arylation of a variety of heteroarenes, such as azoles, thiophene, furan and pyridine, under transition metal-free conditions at room temperature without any light stimulation. This protocol has the unique potential to perform the C2 selective C–H arylation of azole moieties, which can be applied to the synthesis of core parts of different bioactive molecules on the gram scale. We have taken advantage of a resonance-stabilized phenalenyl radical that has been known for half a century to trap the intermediate *via* formation of a C–C σ-bond. The empty NBMO of the K-coordinated PLY complex acts as an electron acceptor, and the SOMO of this complex further transfers its electron to the aryl coupling partner, generating the active aryl radical. For the first time, it was possible to characterize the trapped radical intermediate using single crystal X-ray crystallography as a direct evidence for the radical-mediated transition metal-free coupling catalysis, which was reported nearly one decade ago.

## Experimental section

### General considerations

All solvents were distilled from Na/benzophenone or calcium hydride prior to use. All chemicals were purchased and used as received. The ^1^H and ^13^C {^1^H} NMR spectra were recorded on 400 and 500 MHz spectrometers in CDCl_3_ with residual undeuterated solvent (CDCl_3_, 7.26/77.0) as an internal standard. Chemical shifts (*δ*) are given in ppm, and *J* values are given in Hz. All chemical shifts were reported in ppm using tetramethylsilane as a reference. Chemical shifts (*δ*) downfield from the reference standard were assigned positive values. Open column chromatography and thin layer chromatography (TLC) were performed on silica gel (Merck silica gel 100–200 mesh). Potassium *tert*-butoxide was purchased from Sigma-Aldrich. All benzoxazoles (**4a–d**) were synthesized following the reported literature.^44^ All aryldiazonium tetrafluoroborate salts were synthesized following the reported literature.^29^ The modified methodology for arylation of pyridine is described in the ESI (pages S5 and S6†).

### General procedure for the C–H arylation of azoles

Benzoxazoles **4a–d** (0.48 mmol)/thiazole (0.72 mmol), diazo coupling partner (0.24 mmol), PLY2 (2.5 mg, 5 mol%, 0.012 mmol) and KO^*t*^Bu (3 mg, 10 mol%, 0.024 mmol) were added to a 25 mL pressure tube and DMSO (1 mL) was poured into the reaction mixture inside a nitrogen-filled glovebox. The final reaction mixture was allowed to stir for 24 h at room temperature. After completion of the reaction, the product was extracted using 25 mL dichloromethane (DCM) and dried over anhydrous sodium sulphate. The solvent was removed under reduced pressure and the crude product was purified by column chromatography on silica gel (100–200 mesh) using a hexane/EtOAc mixture to yield the pure desired product.

### General procedure for the C–H arylation of thiophene and furan

Heteroarene **9a/9b** (1.2 mmol), diazo coupling partner (0.24 mmol), PLY2 (2.5 mg, 5 mol%, 0.012 mmol) and KO^*t*^Bu (3 mg, 10 mol%, 0.024 mmol) were added to a 25 mL pressure tube and DMSO (1 mL) was poured into the reaction mixture inside the glovebox. The final reaction mixture was allowed to stir for 8 h at room temperature. After completion of the reaction, the product was extracted using 25 mL dichloromethane (DCM) and dried over anhydrous sodium sulphate. The solvent was removed under reduced pressure and the crude product was purified by column chromatography on silica gel (100–200 mesh) using a hexane/EtOAc mixture to yield the pure desired product.

### Procedure for the TEMPO-trapped intermediate preparation

TEMPO (0.24 mmol), diazo coupling partner **2b** (0.24 mmol), PLY2 (50 mg, 0.24 mmol) and KO^*t*^Bu (30 mg, 0.24 mmol) were added to a 25 mL pressure tube and DMSO (1 mL) was poured into the reaction mixture inside the glovebox. The final reaction mixture was allowed to stir for 12 h at room temperature. After completion of the reaction, the product was extracted using 25 mL dichloromethane (DCM) and dried over anhydrous sodium sulphate. The solvent was removed under reduced pressure and product **16** was purified by column chromatography on silica gel (100–200 mesh) using hexane/EtOAc.

### Crystallization of complex **17**

PLY2 (20 mg, 0.0952 mmol) and KO^*t*^Bu (21.2 mg, 0.19 mmol) were dissolved together in dry acetonitrile solvent inside a nitrogen filled glovebox and 18-crown-6 ether was added (0.19 mmol). After standing overnight, dark-colored block-shaped crystals of **17** appeared at –20 °C.

### Crystallographic characterization of **17**

A suitable single crystal of **17** was selected and intensity data were collected on a SuperNova (Dual, Cu at zero, Eos) diffractometer. Using Olex2,^45^ the structure was solved with the Superflip^46^ structure solution program using charge flipping and refined with the ShelXL^47^ refinement package using least squares minimization. The crystallographic data for the structural analysis of **17** were deposited at the Cambridge Crystallographic Data Centre, CCDC, no. ; 1520738. Copies of this information can be obtained from the Director, CCDC, 12 Union Road, Cambridge CB2 1EZ, UK (fax: +44 1233 336033, email: ; Email: deposit@ccdc.ac.uk or ; www.ccdc.cam.ac.uk).†

## Conflicts of interest

There are no conflicts to declare.
