# Supplementary material for: A new face of phenalenyl-based radicals in the transition metal-free C–H arylation of heteroarenes at room temperature: trapping the radical initiator via C–C σ-bond formation
Source: Chem Sci. 2017 Sep 12;8(11):7798–806. doi: 10.1039/c7sc02661g (PMC5674448; doi:10.1039/c7sc02661g)
Supplement: Supplementary file 1 [file SC-008-C7SC02661G-s001.pdf]

Electronic Supplementary Material (ESI) for Chemical Science.
This journal is © The Royal Society of Chemistry 2017

# Electronic Supplementary Information

## New Face of Phenalenyl Based Radical in Transition Metal Free C-H Arylation of Heteroarenes at Room Temperature: Trapping the Radical Initiator via C-C σ Bond Formation

*Jasimuddin Ahmed*[†], *Sreejyothi P*[†], *Gonela Vijaykumar*[†], *Anex Jose*[†], *Manthan Raj*[‡] *and Swadhin K. Mandal**[†]

[†]Department of Chemical Sciences, Indian Institute of Science Education and Research-Kolkata, Mohanpur-741246, India.

[‡] Zakir Husain Delhi College, University of Delhi, Delhi-110002, India.

*E-mail: swadhin.mandal@iiserkol.ac.in

**Contents:**

I. Experimental section……………………………………………………………..S2-S11
II. The analytical and spectral characterization data of the products…………………......S12-S24
III.$^{1}$H NMR and $^{13}$C NMR spectra of the products……………………………………...S25-S63
IV. X-ray structure and data of compound **17**………………………………………..S64-S68
V. Computational Details…………………………………………………………….S68-S71
VI. References……………………………………………………………………..S71-S73

## I. Experimental Section

### General consideration:

All solvents were distilled from Na/benzophenone or calcium hydride prior to use. All chemicals were purchased and used as received. The $^{1}H$ and $^{13}C$ {$^{1}H$} NMR spectra were recorded on 400 and 500 MHz spectrometers in $CDCl_3$ with residual undeuterated solvent ($CDCl_3$, 7.26/77.0) as an internal standard. Chemical shifts ($\delta$) are given in ppm, and $J$ values are given in Hz. All chemical shifts were reported in ppm using tetramethylsilane as a reference. Chemical shifts ($\delta$) downfield from the reference standard were assigned positive values. Open-column chromatography and thin-layer chromatography (TLC) were performed on silica gel (Merck silica gel 100-200 mesh). Evaporation of solvents was performed under reduced pressure using a rotary evaporator. All benzoxazoles (**4a**-**4d**) were synthesized following reported literature.[1]

### Synthesis of Aryldiazonium tetrafluoroborate:[2]

Substituted aniline (10 mmol) was dissolved in 5 mL of distilled water at room temperature then 4 mL of 46% hydrofluoroboric acid was added to the mixture. The resulting reaction mixture was cooled down to 0-5 $^{o}C$ using an ice bath. Sodium nitrite (0.69 g) was dissolved in 1.5 mL of water and cooled down to 0-5 $^{o}C$ separately, this cold sodium nitrite solution was added drop-wise to the main reaction mixture. The resulting mixture was stirred for 30 min maintaining the temperature 0-5 $^{o}C$ and the precipitate was collected by filtration after washing the residue with ice cold water. The final residue was dissolved in minimum amount of acetone and then diethyl ether was added. Pure product was collected as crystals from this mixture of acetone and diethyl ether solvent. The final aryldiazonium tetrafluoroborate crystals were washed with diethyl ether and dried under vacuum.

### Reaction Procedure for Optimization Study on C-H Arylation of Thiazole.

Thiazole (0.72 mmol), diazo coupling partner (**2a**, 0.24 mmol), ligand and base were taken in a 25 mL quartz tube and then DMSO (1 mL) was poured in to the reaction mixture inside a nitrogen filled glovebox. The final reaction mixture was allowed to stir for required time at room temperature. After completion of the reaction, product was extracted in 25 mL dichloromethane (DCM) and dried over anhydrous sodium sulphate. The solvent was removed under reduced

pressure and crude product was purified by column chromatography on silica gel (100-200 mesh) using hexane/EtOAc mixture to yield the pure desired products.

**General Procedure for C-H Arylation of azoles (3 and 4a-d).**

Thiazole (0.72 mmol)/benzoxazoles **4a-d** (0.48 mmol), diazo coupling partner (0.24 mmol), PLY 2 (2.5 mg, 5 mol%, 0.012 mmol), KO$^t$Bu (3 mg, 10 mol%, 0.024 mmol) were taken in a 25 mL pressure tube and then DMSO (1 mL) was poured in to the reaction mixture inside a nitrogen filled glovebox. The final reaction mixture was allowed to stir for 24 h at room temperature. After completion of the reaction, product was extracted in 25 mL dichloromethane (DCM) and dried over anhydrous sodium sulphate. The solvent was removed under reduced pressure and crude product was purified by column chromatography on silica gel (100-200 mesh) using hexane/EtOAc mixture to yield the pure desired products.

**Procedure for Large Scale Synthesis of 6f (1.31 g):**

6-Nitrobenzoxazole (14.3 mmol, 2.35 g), diazo coupling partner **2f** (7.15 mmol, 1.5 g), PLY 2 (75, 5 mol%, 0.357 mmol), KO$^t$Bu (80 mg, 10 mol%, 0.71 mmol) were taken in a 50 mL pressure tube and then DMSO (10 mL) was poured in to the reaction mixture inside glovebox. The final reaction mixture was allowed to stir for 24 h at room temperature. After completion of the reaction, product was extracted in 100 mL dichloromethane (DCM) and dried over anhydrous sodium sulphate. The solvent was removed under reduced pressure and crude product was purified by column chromatography on silica gel (100-200 mesh) using hexane/EtOAc mixture to yield the pure product **6f** with 71% yield (1.31 g).

**Procedure for Large Scale Synthesis of 8b (815 mg):**

6-Methylbenzoxazole (13.4 mmol, 1.8 g), diazo coupling partner **2b** (6.68 mmol, 1.6 g), PLY 2 (70, 5 mol%, 0.334 mmol), KO$^t$Bu (75 mg, 10 mol%, 0.668 mmol) were taken in a 50 mL pressure tube and then DMSO (10 mL) was poured in to the reaction mixture inside the glovebox. The final reaction mixture was allowed to stir for 36 h at room temperature. After completion of the reaction, product was extracted in 100 mL dichloromethane (DCM) and dried over anhydrous sodium sulphate. The solvent was removed under reduced pressure and crude

product was purified by column chromatography on silica gel (100-200 mesh) using hexane/EtOAc mixture to yield the pure product **8b** with 48% yield (815 mg).

**Table S1. Reaction Optimization Table for Arylation of Thiophene.**

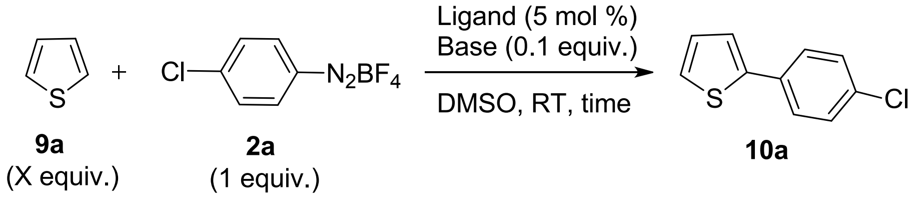


| Entry | Equiv. of 9a | Time (h) | Yield (%) |
|---|---|---|---|
| 1. | 3 | 24 | 71 |
| 2 | 5 | 24 | 84 |
| 3 | 5 | 12 | 85 |
| **4** | **5** | **8** | **82** |

**General Procedure for C-H Arylation of Thiophene and Furan:**

Heteroarene **9a/9b** (1.2 mmol), diazo coupling partner (0.24 mmol), PLY 2 (2.5 mg, 5 mol%, 0.012 mmol), KO$^t$Bu (3 mg, 10 mol%, 0.024 mmol) were taken in a 25 mL pressure tube and then DMSO (1 mL) was poured to the reaction mixture inside the glovebox. The final reaction mixture was allowed to stir for 8 h at room temperature. After completion of the reaction, product was extracted in 25 mL dichloromethane (DCM) and dried over anhydrous sodium sulphate. The solvent was removed under reduced pressure and crude product was purified by column chromatography on silica gel (100-200 mesh) using hexane/EtOAc mixture to yield the pure desired product.

**Arylation of Pyridine and benzene:**

Pyridine/benzene, diazo coupling partner (0.48 mmol), PLY2 (5 mg, 5 mol%, 0.024 mmol), KO$^t$Bu (6 mg, 10 mol%, 0.048 mmol) were taken in a 25 mL pressure tube and then DMSO (1 mL) was poured to the reaction mixture inside the glovebox. The final reaction mixture was allowed to stir for 8 h at room temperature. After completion of the reaction, organic part was extracted in 25 mL dichloromethane (DCM) and dried over anhydrous sodium sulphate. The

solvent was removed under reduced pressure and the $^1$H NMR of crude reaction mixture revealed that there is no desired product formation.

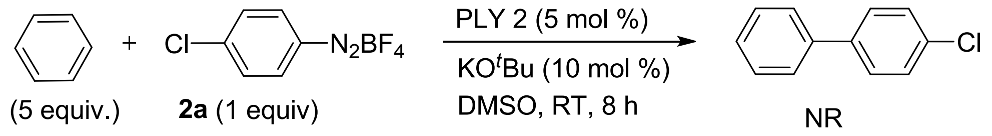


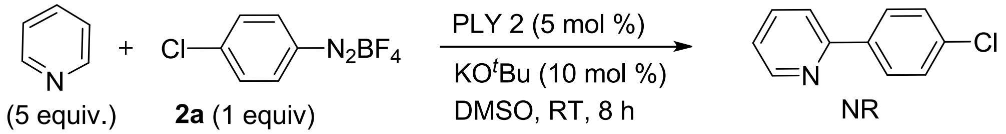


**Modified methodology for arylation of pyridine:**

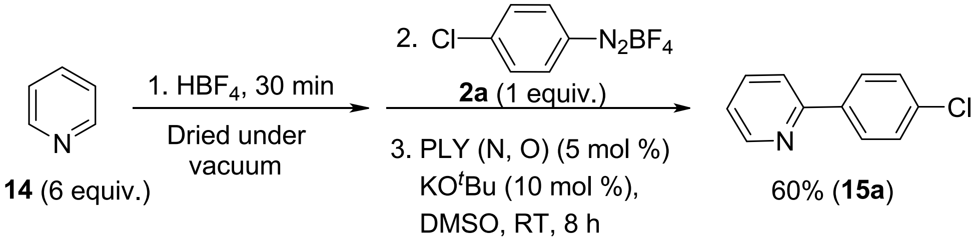


Pyridine **14** (2.88 mmol) and $HBF_4$ (2.88 mmol, 45% in water) were stirred together for 30 min at room temperature in a 50 mL Schlenck tube when a white precipitate was formed. This resulting reaction mixture was dried under vacuum to get a white solid pyridyl salt ($PyH^+$ $BF_4^-$). In a different vial PLY 2 (0.024 mol) and KO$^t$Bu (0.048 mol) were taken in DMSO solvent and stirred for 1 h to obtain a deep green reaction mixture (phenalenyl radical based active catalyst) inside glove box. Schlenk tube containing the white solid ($PyH^+$ $BF_4^-$) was taken inside the nitrogen filled glove box and diazonium salt **2a** (0.48 mmol) was added to it, and subsequently the green colored active catalyst mixture was poured in to it and stirred at room temperature for 8h when the reaction mixture turned brown. After completion of the reaction, product was extracted in 25 mL dichloromethane (DCM) and dried over anhydrous sodium sulphate. The solvent was removed under reduced pressure and crude product was purified by column chromatography on silica gel (100-200 mesh) using hexane/EtOAc mixture to yield the pure

desired product. Yield of the isolated product was recorded. A blank reaction without PLY2 did not proceed (yield below 3%).

**Procedure for Competition Experiment between Aryl Diazonium Salt Coupling Partners 2a and 2e.**

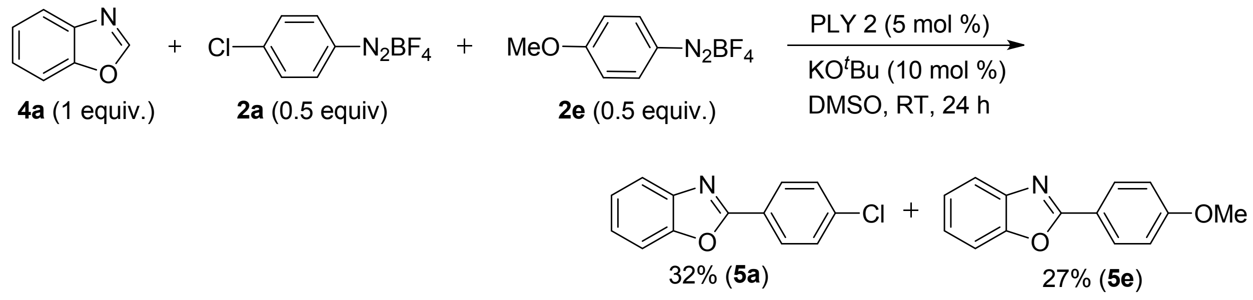


Benzoxazole **4a** (0.48 mmol), diazo coupling partners **2a** and **2e** (0.24 mmol), PLY 2 (5 mg, 5 mol%, 0.024 mmol), KO$^t$Bu (6 mg, 10 mol%, 0.048 mmol) were taken in a 25 mL pressure tube and then DMSO (1 mL) was poured to the reaction mixture inside the glovebox. The final reaction mixture was allowed to stir for 24 h at room temperature. After completion of the reaction, product was extracted in 25 mL dichloromethane (DCM) and dried over anhydrous sodium sulphate. The solvent was removed under reduced pressure and two different products **5a** and **5e** were purified by column chromatography on silica gel (100-200 mesh) using only hexane.

**Procedure for Reactions in Presence of Radical Scavenger TEMPO.**

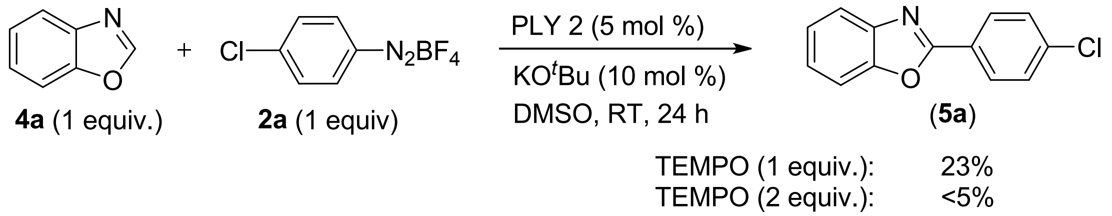


Benzoxazole **4a** (0.24 mmol), diazo coupling partners **2a** (0.24 mmol), PLY 2 (2.5 mg, 5 mol%, 0.012 mmol), KO$^t$Bu (3 mg, 10 mol%, 0.024 mmol) and required amount of TEMPO were taken in a 25 mL pressure tube and then DMSO (1 mL) was poured to the reaction mixture inside the glovebox. The final reaction mixture was allowed to stir for 24 h at room temperature. After completion of the reaction, product was extracted in 25 mL dichloromethane (DCM) and dried

over anhydrous sodium sulphate. The solvent was removed under reduced pressure and product **5a** was purified by column chromatography on silica gel (100-200 mesh) using hexane/EtOAc.

**Procedure for TEMPO-trapped intermediate preparation.**

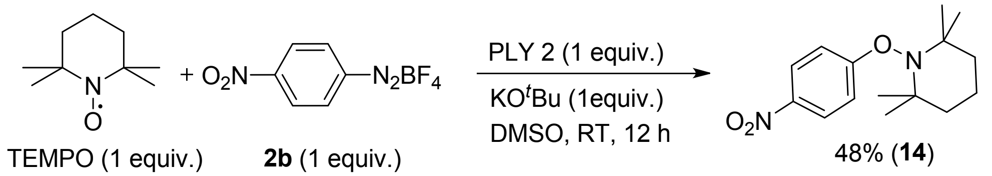


TEMPO (0.24 mmol), diazo coupling partners **2b** (0.24 mmol), PLY 2 (0.24 mmol), KO$^t$Bu (0.24 mmol) and required amount of TEMPO were taken in a 25 ml pressure tube and then DMSO (1 mL) was poured to the reaction mixture inside the glovebox. The final reaction mixture was allowed to stir for 12 h at room temperature. After completion of the reaction, product was extracted in 25 mL dichloromethane (DCM) and dried over anhydrous sodium sulphate. The solvent was removed under reduced pressure and product **14** was purified by column chromatography on silica gel (100-200 mesh) using hexane/EtOAc.

**Stoichiometric Reaction Study in DMSO.**

PLY2 and K$^t$OBu were taken with 1:1 equiv. in DMSO solvent, instantly colour become red. EPR and $^1$H NMR spectra were recorded. After that, another equiv. of KO$^t$Bu was added to the reaction mixture and allowed for 20 min stirring. The sharp color change happens from red to dark green. EPR of this red coloured (within 5 min) dark green colored (after 20 min) reaction mixture was measured in toluene/DMSO (10:1) solution (Figure S1).

**Figure S1.** EPR Spectrum of PLY2 and K$^t$OBu (DMSO, 1:2) in toluene/DMSO.

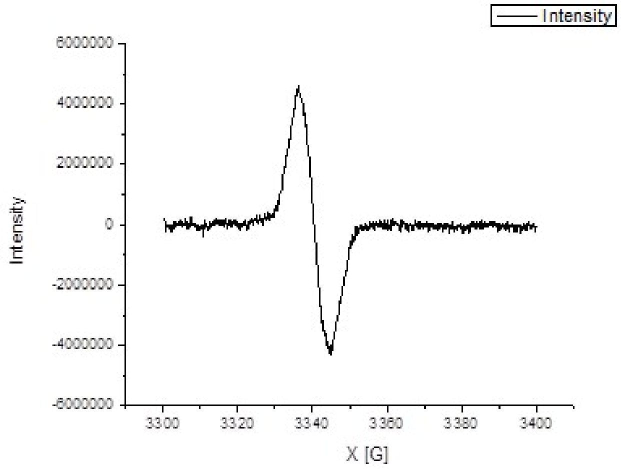


PLY2 and K$^t$OBu (1:2),

After 20 min

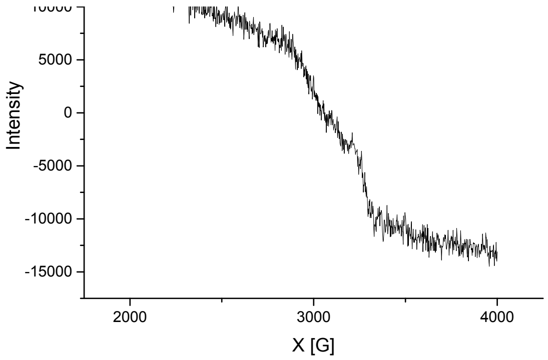


PLY2 and K$^t$OBu (1:1),

Within 5 min

**Figure S2.** $^{1}$H NMR Spectrum of Reaction Mixture Between PLY2 and K$^{t}$OBu as a Function of Time Showing NMR Broadening in DMSO-$d_6$.

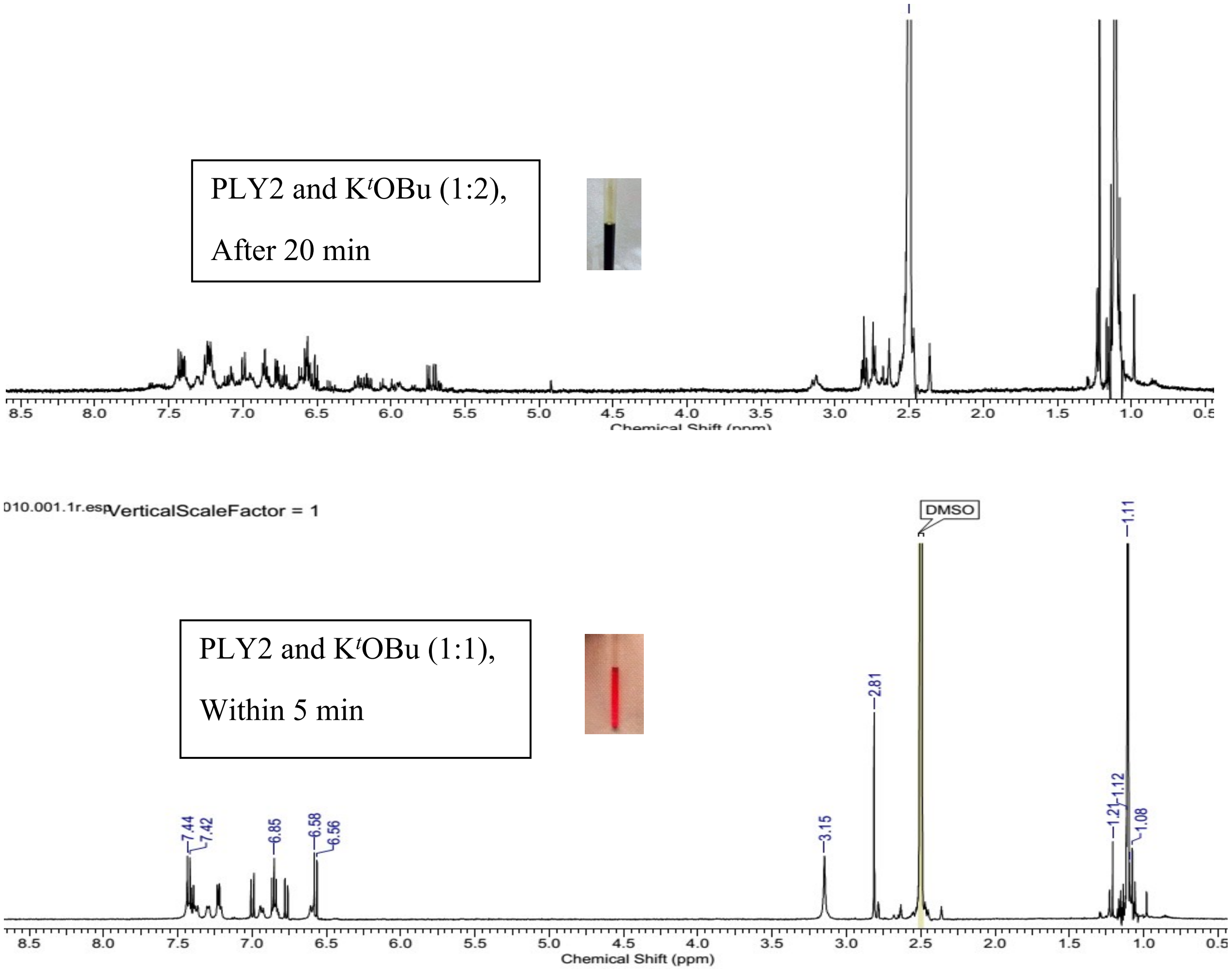


**Isolation of K-Phenalenyl Complex (Complex I).**

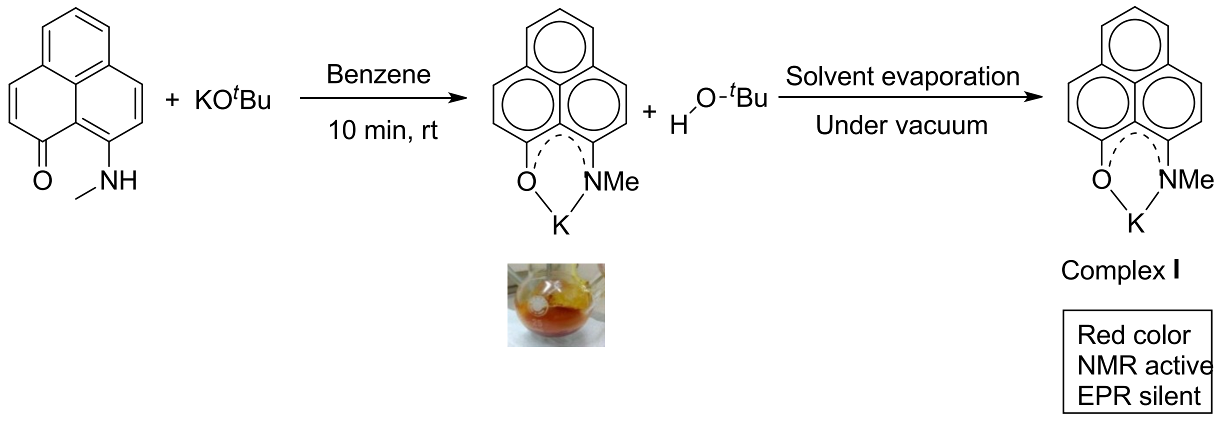

PLY2 and K$^t$OBu were taken in 1:1 ratio using benzene as a solvent. The resulting mixture was allowed to stir for 10 min at room temperature, when a red precipitate was formed. The solvent was evaporated under high vacuum, a red solid product (Complex **I**) was obtained. Complex **I** was characterized by $^1$H NMR spectroscopy (Figure S3). $^1$H NMR spectra of PLY2 and complex **I** were compared (see below) which clearly shows absence of NH proton and overall upfield shift on K ion complexation.

**Figure S3.** Stack Plot of $^1$H NMR Spectra of PLY2 Ligand and Complex **I** in DMSO-$d_6$.

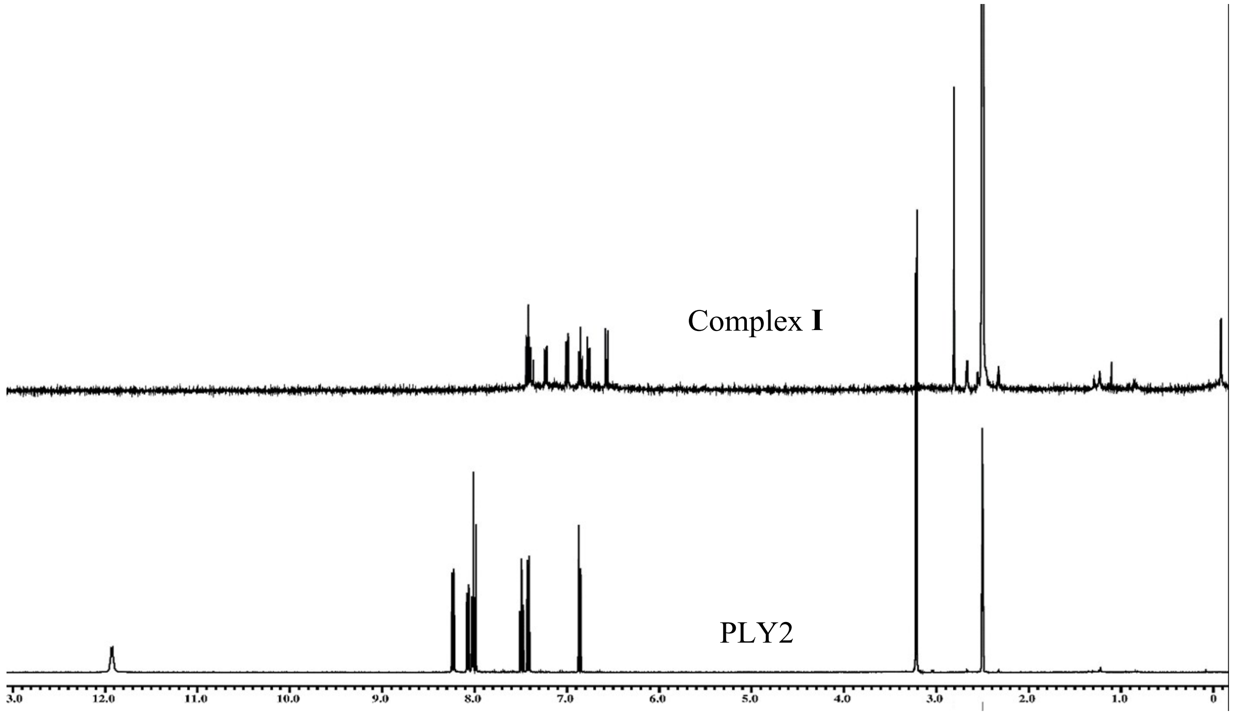

EPR spectrum of complex **I**

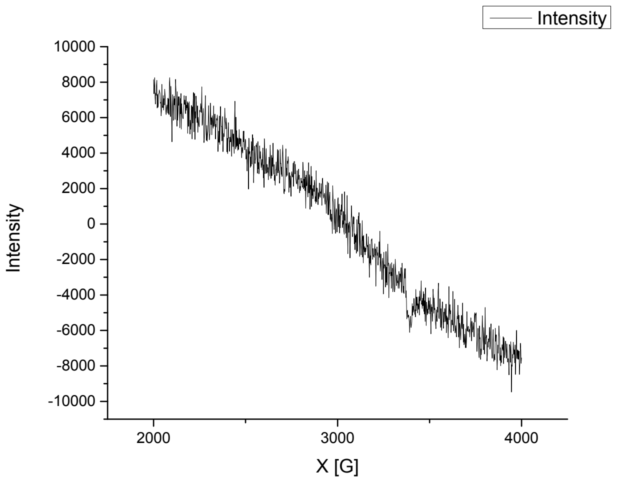


**Stoichiometric reactions with Complex I.**

Complex **I** (5 mol%, 0.012 mmol), KO$^t$Bu / TDAE ((5 mol%, 0.012 mmol) was taken in DMSO and allowed to stir for 20 min and the reaction mixture becomes deep green from red color. In a 25 mL pressure tube **2a** (0.24 mmol) and thiophene/thiazole were taken separately and the previous green reaction mixture was poured in this reaction mixture. The resulted mixture was allowed for stirring for appropriate time (8 h for thiophene, 24 h for thiazole). The product was extracted from ethyl acetate and isolated column chromatography using silica gel (100-200 mesh).

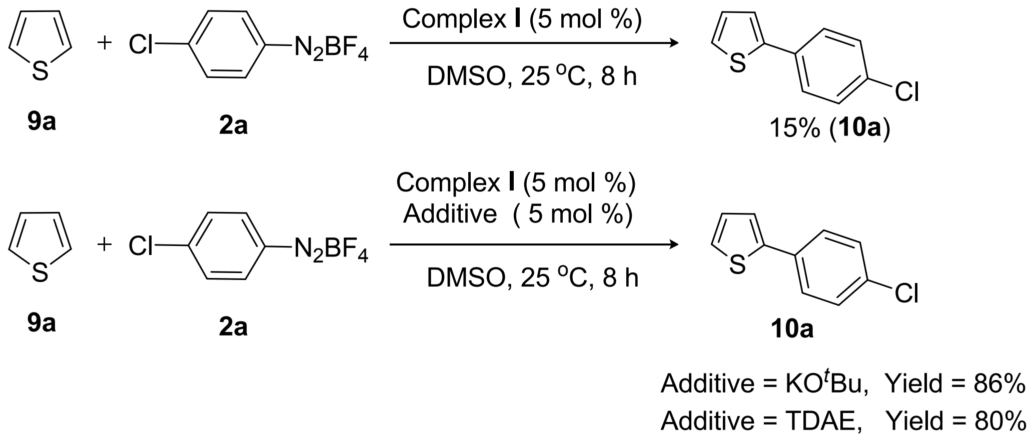

**II. The analytical and spectral characterization data of the products**

**2-(4-Chlorophenyl)thiazole (3a):**[3]

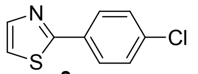


**3a**

Colorless oil, Yield: 64%. The crude product was purified by column chromatography using silica gel (100-200 mesh) with 1% of EtOAc in hexane.

$^1$H NMR (400 MHz, $CDCl_3$, 298 K) $\delta$ (ppm) 7.32-7.34 (m, 1H), 7.42 (dd, $J_1$ = 7 Hz, $J_2$ = 2.2 Hz, 2H), 7.85-7.91 (m, 3H).

$^{13}$C NMR (100 MHz, $CDCl_3$, 298 K) $\delta$ (ppm) 119.1, 127.7, 129.2, 132.1, 135.9, 143.8, 167.1.

**2-(4-Nitrophenyl)thiazole (3b):**[3]

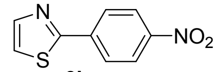


**3b**

Pale yellow oil, Yield: 62%. The crude product was purified by column chromatography using silica gel (100-200 mesh) with 1% of EtOAc in hexane.

$^1$H NMR (400 MHz, $CDCl_3$, 298 K) $\delta$ (ppm) 7.49 (m, 1H), 7.98 (m, 1H), 8.14 (d, $J$ = 9.1 Hz, 2H), 8.31 (d, $J$ = 7.6 Hz, 2H).

$^{13}$C NMR (100 MHz, $CDCl_3$, 298 K) $\delta$ (ppm) 121.0, 124.4, 127.2, 139.0, 144.6, 148.4, 165.4.

**2-(4-Methylphenyl)thiazole (3c):** [3]

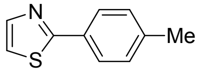


**3c**

Colorless oil, Yield: 64%. The crude product was purified by column chromatography using silica gel (100-200 mesh) with 1% of EtOAc in hexane.

$^1$H NMR (400 MHz, $CDCl_3$, 298 K) $\delta$ (ppm) 2.32 (s, 3H), 7.17-7.18 (d, $J$ = 7.6 Hz, 2H), 7.21 (d, $J$ = 3.0 Hz, 1H), 7.76-7.79 (m, 3H).

$^{13}$C NMR (100 MHz, $CDCl_3$, 298 K) $\delta$ (ppm) 21.4, 118.3, 126.5, 129.6, 130.9, 140.2, 143.5, 168.6.

**2-Phenylthiazole (11d):** [3]

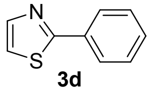

**3d**

Colorless oil, Yield: 57%. The crude product was purified by column chromatography using silica gel (100-200 mesh) with 1% of EtOAc in hexane.

$^1$H NMR (400 MHz, $CDCl_3$, 298 K) $\delta$ (ppm) 7.96-7.97 (m, 2H), 7.86-7.87 (m, 1H), 7.43-7.45 (m, 3H), 7.33 (m, 1H).

$^{13}$C NMR (100 MHz, $CDCl_3$, 298 K) $\delta$ (ppm) 118.8, 126.6, 128.9, 130.0, 133.5, 143.6, 168.4.

**2-(4-Methoxyphenyl)thiazole (3e):**[3]

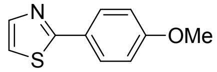


**3e**

Colorless oil, Yield: 60%. The crude product was purified by column chromatography using silica gel (100-200 mesh) with 1% of EtOAc in hexane.

$^1$H NMR (400 MHz, $CDCl_3$, 298 K) $\delta$ (ppm) 7.89-7.93 (m, 2H), 7.80 (m, 1H), 7.25-7.26 (m, 1H), 6.95-6.97 (m, 2H), 3.86 (s, 3H).

$^{13}$C NMR (100 MHz, $CDCl_3$, 298 K) $\delta$ (ppm) 55.3, 114.2, 117.8, 126.5, 128.0, 143.3, 161.0, 168.3.

**2-(4-Chlorophenyl)benzoxazole (5a):** [4]

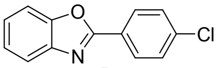


**5a**

Off-white solid, Yield: 71%. The crude product was purified by column chromatography using silica gel (100-200 mesh) with 2% of EtOAc in hexane.

$^1$H NMR (500 MHz, $CDCl_3$, 298 K) $\delta$ (ppm) 8.19 (d, $J$ = 9.16 Hz, 2H), 7.77-7.79 (m, 1H), 7.52-7.57 (m, 1H), 7.48-7.52 (m, 2H), 7.36-7.38 (m, 2H).

$^{13}$C NMR (125 MHz, $CDCl_3$, 298 K) $\delta$ (ppm) 110.6, 120.1, 124.7, 125.3, 125.7, 128.8, 129.3, 137.8, 142.0, 150.7, 162.1.

**2-(4-Methoxyphenyl)benzoxazole (5e):**[4]

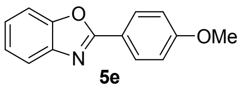


**5e**

Colorless solid, Yield: 55%. The crude product was purified by column chromatography using silica gel (100-200 mesh) with 2% of EtOAc in hexane.

$^{1}$H NMR (500 MHz, $CDCl_3$, 298 K) $\delta$ (ppm) 8.20 (d, $J$ = 8.0 Hz, 2H), 7.72-7.74 (dd, $J_1$ = $J_2$ = 4 Hz, 1H), 7.50-7.54 (m, 1H), 7.32-7.34 (m, 2H), 7.02-7.06 (m, 2H), 3.88 (s, 3H).

$^{13}$C NMR (125 MHz, $CDCl_3$, 298 K) $\delta$ (ppm) 55.4, 110.3, 114.3, 119.6, 119.7, 124.4, 124.6, 129.4, 142.2, 150.6, 162.3, 163.2.

**2-(4-Chlorophenyl)-5-nitrobenzoxazole (6a):**[5]

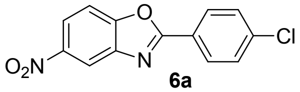


**6a**

Colorless crystalline solid, Yield: 83%. The crude product was purified by column chromatography using silica gel (100-200 mesh) with 2% of EtOAc in hexane.

$^{1}$H NMR (500 MHz, $CDCl_3$, 298 K) $\delta$ (ppm) 8.64 (d, $J$ = 2 Hz, 1H), 8.32-8.34 (m, 1H), 8.21 (d, $J$ = 8.5 Hz, 2H), 7.67 (d, $J$ = 9 Hz, 1H), 7.53 (d, $J$ = 8.5 Hz, 2H).

$^{13}$C NMR (125 MHz, $CDCl_3$, 298 K) $\delta$ (ppm) 110.8, 116.4, 121.3, 124.5, 129.3, 129.6, 139.1, 142.5, 145.6, 154.3, 165.0.

**2-(4-Methylphenyl)-5-nitrobenzoxazole (6c):**[6]

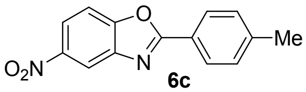


**6c**

Red crystalline solid, Yield: 56%. The crude product was purified by column chromatography using silica gel (100-200 mesh) with 2% of EtOAc in hexane.

$^{1}$H NMR (400 MHz, $CDCl_3$, 298 K) $\delta$ (ppm) 7.36 (d, $J$ = 8.4 Hz), 7.66 (d, $J$ = 8.4 Hz), 8.15 (d, $J$ = 8.4 Hz, 2H), 8.29-8.32 (m, 1H), 8.62 (m, 1H).

$^{13}$C NMR (100 MHz, $CDCl_3$, 298 K) $\delta$ (ppm) 21.7, 110.6, 116.1, 120.9, 132.2, 128.0, 129.9, 142.7, 143.3, 145.4, 154.3, 166.3.

**5-Nitro-2-phenylbenzoxazole (6d):**[5]

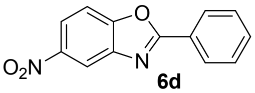


**6d**

Reddish brown crystalline solid, Yield: 60%. The crude product was purified by column chromatography using silica gel (100-200 mesh) with 2% of EtOAc in hexane.

$^{1}$H NMR (500 MHz, $CDCl_3$, 298 K) $\delta$ (ppm) 8.66 (d, $J$ = 2 Hz, 1H), 8.32 (dd, $J_1$ = 9Hz, $J_2$ = 2 Hz, 1H), 8.27 (d, $J$ = 5.5 Hz, 2H), 7.69 (d, $J$ = 8 Hz, 1H).

$^{13}$C NMR (125 MHz, $CDCl_3$, 298 K) $\delta$ (ppm) 110.7, 116.2, 121.1, 125.9, 128.0, 129.1, 132.6, 142.5, 145.4, 154.2, 165.9.

**2-(4-Methoxyphenyl)-5-nitrobenzoxazole (6e):**[5]

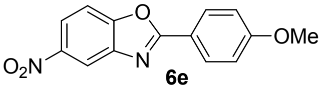


**6e**

Brown solid, Yield: 68%. The crude product was purified by column chromatography using silica gel (100-200 mesh) with 2% of EtOAc in hexane.

$^{1}$H NMR (400 MHz, $CDCl_3$, 298 K) $\delta$ (ppm) 8.58 (d, $J$ = 2.3 Hz, 1H), 8.26-8.28 (m, 1H), 8.19-8.21 (m, 2H), 7.63 (d, $J$ = 9.1 Hz, 1H), 7.05 (d, $J$ = 8.4 Hz, 2H), 3.92 (s, 3H).

$^{13}$C NMR (100 MHz, $CDCl_3$, 298 K) $\delta$ (ppm) 55.5, 110.4, 114.6, 115.7, 118.4, 120.6, 129.9, 142.8, 145.3, 154.3, 163.2, 166.1.

**2-(4-Fluorophenyl)-5-nitrobenzoxazole (6f):**[7]

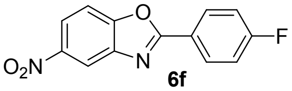


**6f**

Pale yellow solid, Yield: 74%. The crude product was purified by column chromatography using silica gel (100-200 mesh) with 2% of EtOAc in hexane.

$^{1}$H NMR (400 MHz, $CDCl_3$, 298 K) $\delta$ (ppm) 8.63 (m, 1H), 8.26-8.34 (m, 3H), 6.67 (d, $J$ = 12 Hz, 1H), 7.24-7.28 (m, 2H).

$^{13}$C NMR (100 MHz, $CDCl_3$, 298 K) $\delta$ (ppm) 110.8, 116.3, 116.7, 121.2, 122.4, 130.5, 142.6, 145.6, 154.3, 165.1, 166.8.

**2-(4-chlorophenyl)-5-chlorobenzoxazole (7a):**[8]

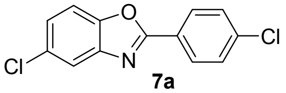

**7a**

White solid, Yield: 83%. The crude product was purified by column chromatography using silica gel (100-200 mesh) with 2% of EtOAc in hexane.

$^{1}$H NMR (400 MHz, $CDCl_3$, 298 K) $\delta$ (ppm) 8.16 (d, $J$ = 9.16 Hz, 2H), 7.66-7.69 (m, 1H), 7.59 (d, $J$ = 3.6 Hz, 1H), 7.51 (d, $J$ = 9.16 Hz, 2H), 7.32 (m, 1H).

$^{13}$C NMR (100 MHz, $CDCl_3$, 298 K) $\delta$ (ppm) 111.3, 120.0, 125.1, 125.6, 128.9, 129.3, 130.2, 138.2, 143.1, 149.3, 163.4.

**2-(4-nitrophenyl)-5-chlorobenzoxazole (7b):**[9]

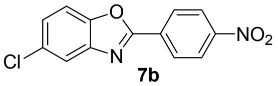

**7b**

Red solid, Yield: 71%. The crude product was purified by column chromatography using silica gel (100-200 mesh) with 2% of EtOAc in hexane.

$^{1}$H NMR (400 MHz, $CDCl_3$, 298 K) $\delta$ (ppm) 8.40-8.42 (m, 4H), 7.73 (m, 1H), 8.65 (m, 1H), 7.39-7.41 (m, 1H).

$^{13}$C NMR (100 MHz, $CDCl_3$, 298 K) $\delta$ (ppm) 111.7, 120.6, 124.2, 126.7, 128.6, 130.8, 132.3, 142.9, 149.6, 149.7, 161.9.

**2-(4-Chlorophenyl)-5-methylbenzoxazole (8a):**[4]

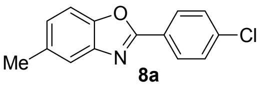

**8a**

White solid, Yield: 47%. The crude product was purified by column chromatography using silica gel (100-200 mesh) with 2% of EtOAc in hexane.

$^{1}$H NMR (400 MHz, $CDCl_3$, 298 K) $\delta$ (ppm) 2.48 (S, 3H), 7.16 (d, $J$ = 4.6 Hz, 1H), 7.43-7.45 (m, 1H), 7.49 (m, 2H), 7.54 (s, 1H), 8.16 (dd, $J_1$ = 8.4 Hz, $J_2$ = 2.3 Hz, 2H).

$^{13}$C NMR (100 MHz, $CDCl_3$, 298 K) $\delta$ (ppm) 21.5, 110.0, 120.0, 125.8, 126.5, 128.7, 129.2, 134.6, 137.6, 142.1, 149.0, 162.1.

**5-Methyl-2-(4-nitrophenyl)benzoxazole (8b):**[4]

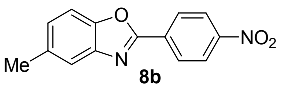

**8b**

Yellow solid, Yield: 47%. The crude product was purified by column chromatography using silica gel (100-200 mesh) with 2% of EtOAc in hexane.

$^1$H NMR (400 MHz, $CDCl_3$, 298 K) $\delta$ (ppm) 2.48 (S, 3H), 7.21-7.25 (m, 1H), 7.35-7.37 (m, 1H), 7.44-7.46 (m, 1H), 8.35-8.38 (m, 4H).

$^{13}$C NMR (100 MHz, $CDCl_3$, 298 K) $\delta$ (ppm) 21.6, 110.4, 120.6, 124.3, 127.6, 128.4, 133.1, 135.4, 142.3, 149.4, 160.8, 162.7.

**2-(4-Chlorophenyl)thiophene (10a):**[10]

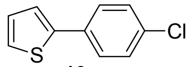

**10a**

Off-white colored solid. Yield: 82%. The crude product was purified by column chromatography using silica gel (100-200 mesh) with only hexane.

$^1$H NMR (400 MHz, $CDCl_3$, 298 K) $\delta$ (ppm) 7.07-7.09 (m, 1H), 7.27-7.30 (m, 2H), 7.30-7.36 (m, 2H), 7.54 (d, $J$ = 8 Hz, 2H)

$^{13}$C NMR (100 MHz, $CDCl_3$, 298 K) $\delta$ (ppm) 123.4, 125.1, 127.0, 128.1, 129.0, 132.9, 133.1, 143.0

**2-(4-Nitrophenyl)thiophene (10b):**[10]

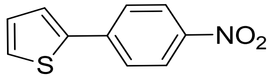

**10b**

Reddish yellow solid. Yield: 84%. The crude product was purified by column chromatography using silica gel (100-200 mesh) with 2% of EtOAc in hexane.

$^1$H NMR (400 MHz, $CDCl_3$, 298 K) $\delta$ (ppm) 7.15 (dd, $J$ = 5.3 Hz, 1H), 7.44 (d, $J$ = 5.3 Hz, 1H), 7.48 (d, $J$ = 2.28 Hz, 1H), 7.74 (d, $J$ = 9.16 Hz, 2H), 8.24 (d, $J$ = 9.16 Hz, 2H).

$^{13}$C NMR (100 MHz, $CDCl_3$, 298 K) $\delta$ (ppm) 124.4, 125.7, 126.0, 127.6, 128.7, 140.6, 141.6, 146.6.

**2-Phenylthiophene (10d):**[10]

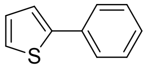

**10d**

Colorless solid, Yield: 65%. The crude product was purified by column chromatography using silica gel (100-200 mesh) with hexane.

$^{1}$H NMR (400 MHz, $CDCl_3$, 298 K) $\delta$ (ppm) 7.07-7.09 (m, 1H), 7.28-7.32 (m, 1H), 7.36-7.46 (m, 2H), 7.59-7.61 (m, 2H).

$^{13}$C NMR (100 MHz, $CDCl_3$, 298 K) $\delta$ (ppm) 123.0, 124.7, 125.9, 127.4, 128.0, 128.8, 134.4, 144.4.

**2-(4-Methoxyphenyl)thiophene (10e):**[11]

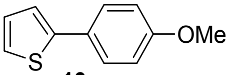

**10e**

Pale yellow solid, Yield: 55%. The crude product was purified by column chromatography using silica gel (100-200 mesh) with hexane.

$^{1}$H NMR (400 MHz, $CDCl_3$, 298 K) $\delta$ (ppm) 3.84 (s, 3H), 6.92 (d, $J$ = 8 Hz, 2H), 7.04-7.06 (m, 1H), 7.19-7.22 (m, 2H), 7.54 (d, $J$ = 8 Hz, 2H).

$^{13}$C NMR (100 MHz, $CDCl_3$, 298 K) $\delta$ (ppm) 55.3, 114.2, 122.0, 123.8, 127.2, 127.9, 132.7, 144.3, 159.1.

**2-(4-Cyanophenyl)thiophene (10g):**[10]

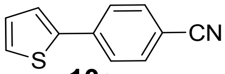

**10g**

Yellow solid. Yield: 83%. The crude product was purified by column chromatography using silica gel (100-200 mesh) with 2% of EtOAc in hexane.

$^{1}$H NMR (400 MHz, $CDCl_3$, 298 K) $\delta$ (ppm) 7.30 (dd, $J_1$ = 5.1 Hz $J_2$ = 3.7 Hz, 1H), 7.40 (dd, $J_1$ = 5.2 Hz, $J_2$ = 1.1 Hz, 1H), 7.42 (dd, $J_1$ = 3.7 Hz, $J_2$ = 1 Hz, 1H), 7.65(d, $J$ = 8.7 Hz, 2H), 7.70 (d, $J$ = 8.7 Hz, 2H).

$^{13}$C NMR (125 MHz, $CDCl_3$, 298 K) $\delta$ (ppm) 110.2, 118.5, 124.9, 125.8, 126.8, 128.3, 132.4, 138.4, 141.7.

**2-(2-Nitrophenyl)thiophene (10h):**[12]

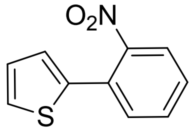


**10h**

Yellow solid. Yield: 75%. The crude product was purified by column chromatography using silica gel (100-200 mesh) with 2% of EtOAc in hexane.

$^1$H NMR (400 MHz, $CDCl_3$, 298 K) $\delta$ (ppm) 7.07-7.09 (m, 2H), 7.41-7.48 (m, 2H), 7.56-7.58 (m, 2H), 7.74 (d, $J$ = 7.6 Hz, 1H).

$^{13}$C NMR (125 MHz, $CDCl_3$, 298 K) $\delta$ (ppm) 114.1, 123.8, 127.1, 127.2, 127.7, 128.4, 131.8, 132.2, 137.1, 150.0.

**2-(4-Chlorophenyl)furan (11a):**[13]

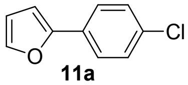


**11a**

Colorless solid, Yield: 91%. The crude product was purified by column chromatography using silica gel (100-200 mesh) with hexane.

$^1$H NMR (400 MHz, $CDCl_3$, 298 K) $\delta$ (ppm) 6.47-6.48 (m, 1H), 6.63-6.64 (m, 1H), 7.35 (d, $J$ = 8.4 Hz, 2H), 7.57-7.58 (m, 1H), 7.60 (d, $J$ = 8.4 Hz, 2H).

$^{13}$C NMR (100 MHz, $CDCl_3$, 298 K) $\delta$ (ppm) 105.4, 111.7, 125.0, 128.8, 129.3, 133.0, 142.3, 152.9.

**2-(4-Nitrophenyl)furan (11b):** [13]

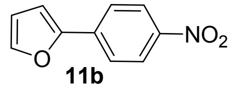


**11b**

Yellow solid, Yield: 88%. The crude product was purified by column chromatography using silica gel (100-200 mesh) with 2% of EtOAc in hexane.

$^1$H NMR (500 MHz, $CDCl_3$, 298 K) $\delta$ (ppm) 6.54 (dd, $J_1$ = 3.5 Hz, $J_2$ = 2Hz, 1H), 6.87 (d, $J$ = 3.5 Hz, 1H), 7.56 (S, 1H), 7.77 (d, $J$ = 9 Hz, 2H), 8.24 (d, $J$ = 9 Hz, 2H).

$^{13}$C NMR (125 MHz, $CDCl_3$, 298 K) $\delta$ (ppm) 108.9, 112.4, 123.9, 124.3, 136.4, 144.1, 146.4, 151.7.

**2-(p-Tolyl)furan (11c):** [13]

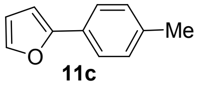

**11c**

White Solid, Yield: 52%. The crude product was purified by column chromatography using silica gel (100-200 mesh) with hexane.

$^{1}$H NMR (400 MHz, $CDCl_3$, 298 K) $\delta$ (ppm) 2.37 (s, 3H), 6.46-6.47 (m, 1H), 6.59 (d, $J$ = 4 Hz, 1H), 7.20 (d, $J$ = 8 Hz, 2H), 7.58 (d, $J$ = 8 Hz, 2H).

$^{13}$C NMR (100 MHz, $CDCl_3$, 298 K) $\delta$ (ppm) 21.2, 104.2, 111.5, 123.7, 128.2, 129.3, 137.1, 141.6, 154.2.

**2-Phenylfuran (11d):** [13]

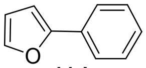

**11d**

Pale yellow solid, Yield: 68%. The crude product was purified by column chromatography using silica gel (100-200 mesh) with hexane.

$^{1}$H NMR (400 MHz, $CDCl_3$, 298 K) $\delta$ (ppm) 6.47 (m, 1H), 6.65 (d, $J$ = 3.3 Hz, 1H), 7.28-7.31 (m, 1H), 7.37-7.44 (m, 1H), 7.48-7.50 (m, 2H), 7.68 (d, $J$ = 8 Hz, 2H).

$^{13}$C NMR (100 MHz, $CDCl_3$, 298 K) $\delta$ (ppm) 104.9, 111.6, 123.7, 127.3, 128.6, 130.9, 142.0, 153.3.

**2-(4-Methoxyphenyl)furan (11e):** [13]

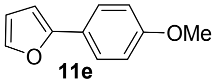

**11e**

Colorless solid, Yield: 64%. The crude product was purified by column chromatography using silica gel (100-200 mesh) with hexane.

$^{1}$H NMR (400 MHz, $CDCl_3$, 298 K) $\delta$ (ppm) 3.83 (s, 3H), 6.44 – 6.45 (m, 1H), 6.51 (d, $J$ = 4 Hz, 1H), 6.92 (d, $J$ = 8 Hz, 2H), 7.42 (m, 1H), 7.60 (d, $J$ = 8 Hz, 2H).

$^{13}$C NMR (100 MHz, $CDCl_3$, 298 K) $\delta$ (ppm) 55.3, 103.3, 111.5, 114.1, 124.0, 125.2, 141.4, 154.0, 159.0.

**2-(4-Cyanophenyl)furan(11g):** [13]

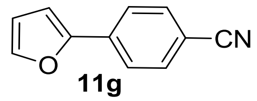

**11g**

Brown solid, Yield: 80%. The crude product was purified by column chromatography using silica gel (100-200 mesh) with 2% of EtOAc in hexane.

$^1$H NMR (400 MHz, $CDCl_3$, 298 K) $\delta$ (ppm) 6.52 (dd, $J_1$ = 3.5 Hz, $J_2$ = 2 Hz, 1H), 6.81 (d, $J$ = 3.5 Hz, 1H), 7.53 (d, $J$ = 1.5 Hz, 1H), 7.66 (d, $J$ = 7 Hz, 2H), 7.74 (d, 2H, $J$ = 7 Hz).

$^{13}$C NMR (125 MHz, $CDCl_3$, 298 K) $\delta$ (ppm) 151.9, 143.6, 134.6, 132.5, 123.9, 118.9, 112.2, 110.2, 108.1.

**2-(2-Nitrophenyl)furan (11h):** [13]

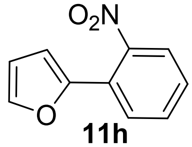

**11h**

Reddish yellow solid, Yield: 73%. The crude product was purified by column chromatography using silica gel (100-200 mesh) with 2% of EtOAc in hexane.

$^1$H NMR (400 MHz, $CDCl_3$, 298 K) $\delta$ (ppm) 6.50 (dd, $J_1$ = 3.8 Hz, $J_2$ = 2.3 Hz, 1H), 6.68 (d, $J$ = 3Hz, 1H), 7.41 (t, $J$ = 6.8 Hz, 1H), 7.51(s, 1H), 7.57 (t, $J$ = 7.6 Hz, 1H), 7.67-7.73 (m, 2H).

$^{13}$C NMR (125MHz, $CDCl_3$, 298 K) $\delta$ (ppm) 109.7, 111.8, 123.8, 124.1, 128.2, 128.8, 131.8, 143.7, 148.3.

**2-(4-Chlorophenyl)pyridine (15a):**[14]

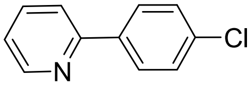

**15a**

Pale yellow, Yield: 60%. The crude product was purified by column chromatography using silica gel (100-200 mesh) with 10% ethyl acetate in hexane.

$^1$H NMR (400 MHz, $CDCl_3$, 298 K) $\delta$ (ppm) 7.22-7.25(m, 1H), 7.44 (d, J = 6.1 Hz, 2H), 7.69 (d, J = 7.6 Hz, 1H), 7.73-7.75 (m, 1H), 7.93 (d, J = 6.1 Hz, 2H), 8.68 (d, J = 4.6 Hz, 1H).

$^{13}$C NMR (100 MHz, $CDCl_3$, 298 K) $\delta$ (ppm) 120.3, 122.3, 128.1, 128.9, 135.1, 136.8, 137.8, 149.7, 156.2.

**2-(4-Nitrophenyl)pyridine (15b):**[15]

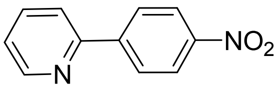

**15b**

Pale yellow, Yield: 64%. The crude product was purified by column chromatography using silica gel (100-200 mesh) with 10% ethyl acetate in hexane.

$^{1}$H NMR (400 MHz, $CDCl_3$, 298 K) $\delta$ (ppm) 7.33-7.35 (m, 1H), 7.82-7.83 (m, 2H), 8.17 (d,J = 9.1 Hz), 8.32 (d, J = 9.1 Hz, 2H), 8.75 (d, J = 4.6 Hz, 1H).

$^{13}$C NMR (100 MHz, $CDCl_3$, 298 K) $\delta$ (ppm) 120.4, 122.7, 123.2, 126.9, 136.4, 144.5, 147.4, 149.3, 154.1.

**2-(*p*-Tolyl)pyridine (15c):**[14]

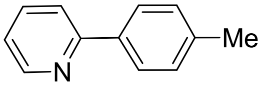

**15c**

Pale yellow, Yield: 33%. The crude product was purified by column chromatography using silica gel (100-200 mesh) with 10% ethyl acetate in hexane.

$^{1}$H NMR (400 MHz, $CDCl_3$, 298 K) $\delta$ (ppm) 2.41 (s, 3H), 7.20 (m, 1H), 7.27 (t, J = 7.9 Hz, 2H,), 7.71-7.73 (m, 2H), 7.89 (d, J = 8.4 Hz, 2H), 8.68 (bs, 1H).

$^{13}$C NMR (100 MHz, $CDCl_3$, 298 K) $\delta$ (ppm) 21.3, 120.3, 121.8, 126.7, 129.5, 136.6, 136.7, 138.9, 149.5, 157.5.

**2-Phenylpyridine (15d):**[15]

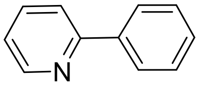

**15d**

Pale yellow, Yield: 53%. The crude product was purified by column chromatography using silica gel (100-200 mesh) with 10% ethyl acetate in hexane.

$^1H$ NMR (400 MHz, $CDCl_3$, 298 K) $\delta$ (ppm) 8.69 (d, J =4.6 Hz, 1H), 7.98 (d, J = 6.9 Hz, 2H), 7.73 – 7.76 (m, 2H), 7.48 (t, J = 7.2 Hz, 2H), 7.41 – 7.44 (m, 1H), 7.22 – 7.25 (m, 1H).

$^{13}C$ NMR (100 MHz, $CDCl_3$, 298 K) $\delta$ (ppm) 120.6, 122.1, 126.9, 128.7, 128.9, 136.7, 139.3, 149.6, 157.5.

**2-(4-Methoxyphenyl)pyridine (15e):**[14]

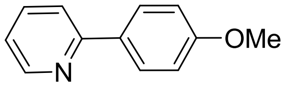


**15e**

Pale yellow, Yield: 47%. The crude product was purified by column chromatography using silica gel (100-200 mesh) with 10% ethyl acetate in hexane.

$^1H$ NMR (400 MHz, $CDCl_3$, 298 K) $\delta$ (ppm) 7.07 (d, J = 8.4 Hz, 2H), 7.24-7.26 (m, 1H), 7.73-7.79 (m, 1H), 8.02 (d, J = 8.4 Hz, 2H), 8.72 (d, J = 4.6 Hz, 1H).

$^{13}C$ NMR (100 MHz, $CDCl_3$, 298 K) $\delta$ (ppm) 55.3, 114.1, 119.8, 121.4, 128.1, 132.0, 136.6, 149.5, 157.1, 160.4.

**2-(4-Fluorophenyl)pyridine (15f):**[16]

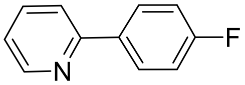


**15f**

Pale yellow, Yield: 56%. The crude product was purified by column chromatography using silica gel (100-200 mesh) with 10% ethyl acetate in hexane.

$^1H$ NMR (400 MHz, $CDCl_3$, 298 K) $\delta$ (ppm) 7.15 (t, J = 6.8 Hz, 2H), 7.20-7.24 (m, 1H), 7.67 (d, J = 8.4 Hz, 1H), 7.74 (t, J = 7.6 Hz, 1H), 7.65-7.69 (m, 2H), 8.66 (d, J = 4.6 Hz, 1H).

$^{13}C$ NMR (100 MHz, $CDCl_3$, 298 K) $\delta$ (ppm) 115.5, 115.7, 120.2, 122.0, 128.6, 128.7, 135.5, 136.8, 149.6, 156.4, 162.2, 164.7.

**2-(3,5-Dimethylphenyl)pyridine (15I):**[14]

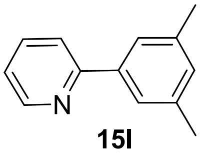


**15l**

Pale yellow, Yield: 35%. The crude product was purified by column chromatography using silica gel (100-200 mesh) with 10% ethyl acetate in hexane.

$^{1}$H NMR (400 MHz, CDCl$_3$, 298 K) $\delta$ (ppm) 7.68 (m, 1H), 7.67-7.75 (m, 2H), 7.57 (s, 2H), 7.22-7.26 (m, 1H), 7.07 (s, 1H), 2.38 (s, 6H).

$^{13}$C NMR (100 MHz, CDCl$_3$, 298 K) $\delta$ (ppm) 21.4, 121.2, 122.1, 125.0, 130.7, 137.1, 138.4, 139.2, 149.3, 157.9.

**TEMPO-trapped intermediate 16:**[12]

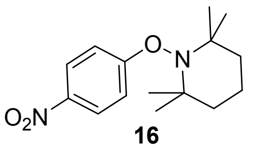


**16**

White solid, Yield: 48%. The crude product was purified by column chromatography using silica gel (100-200 mesh) with 1% of EtOAc in hexane.

$^{1}$H NMR (400 MHz, CDCl$_3$, 298 K) $\delta$ (ppm) 0.98 (s, 6H), 1.24 (s, 6H), 1.42-1.44 (m, 1H), 1.60-1.64 (m, 5H), 7.20-7.27 (m, 2H), 8.13 (d, $J$ = 8.4 Hz, 2H).

$^{13}$C NMR (100 MHz, CDCl$_3$, 298 K) $\delta$ (ppm) 16.8, 32.2, 39.6, 60.8, 114.1, 125.5, 141.1, 168.6.

III. $^{1}$H NMR and $^{13}$C NMR spectra of the products.

**Figure S4.** $^{1}$H NMR ($CDCl_3$) spectrum of 2-(4-chlorophenyl)thiazole (**3a**):

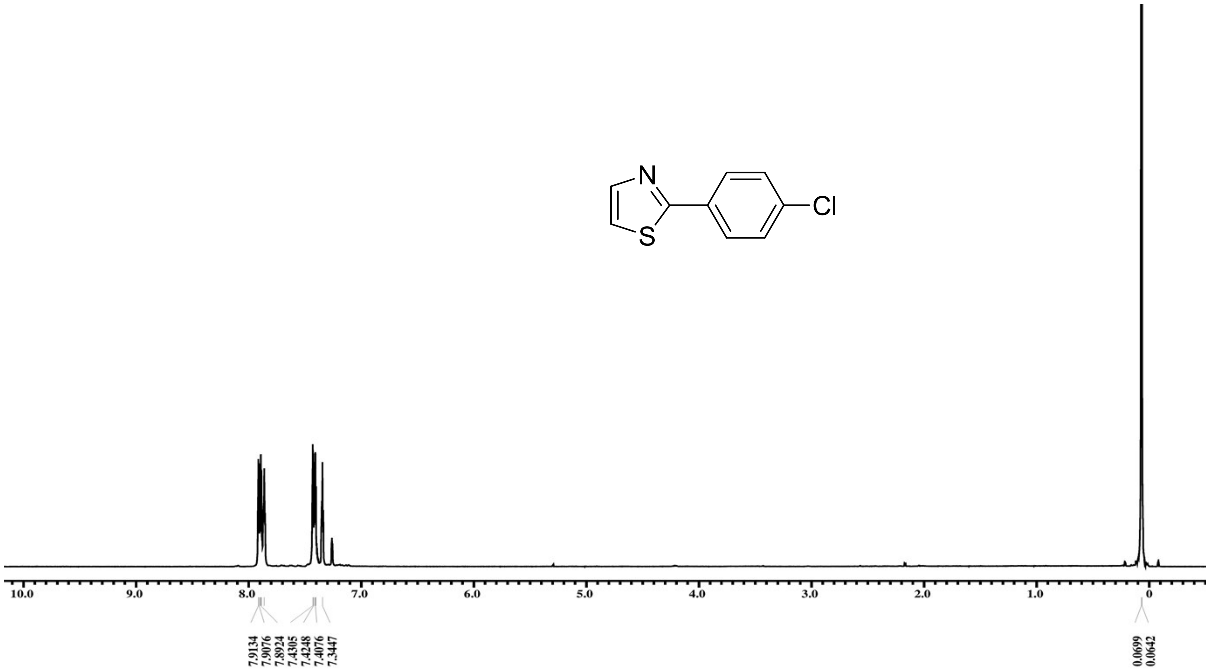


**Figure S5.** $^{13}$C NMR ($CDCl_3$) spectrum of 2-(4-chlorophenyl)thiazole (**3a**):

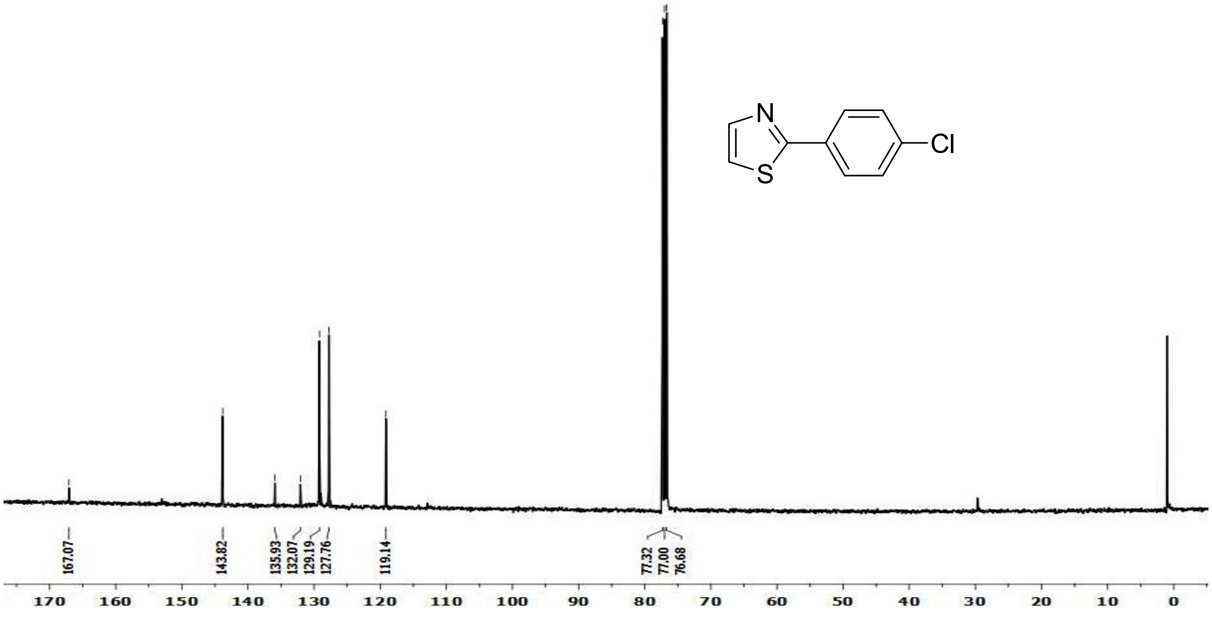

**Figure S6.** $^{1}H$ NMR ($CDCl_3$) spectrum of 2-(4-nitrophenyl)thiazole (**3b**):

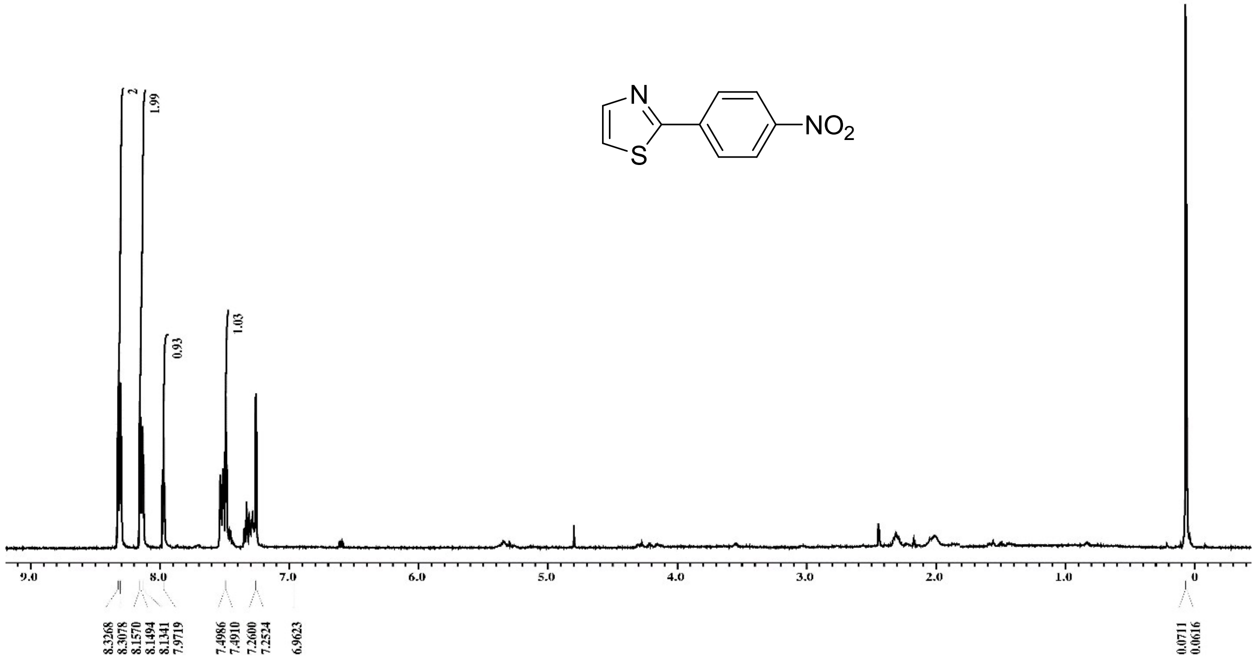


**Figure S7.** $^{13}C$ NMR ($CDCl_3$) spectrum of 2-(4-nitrophenyl)thiazole (**3b**):

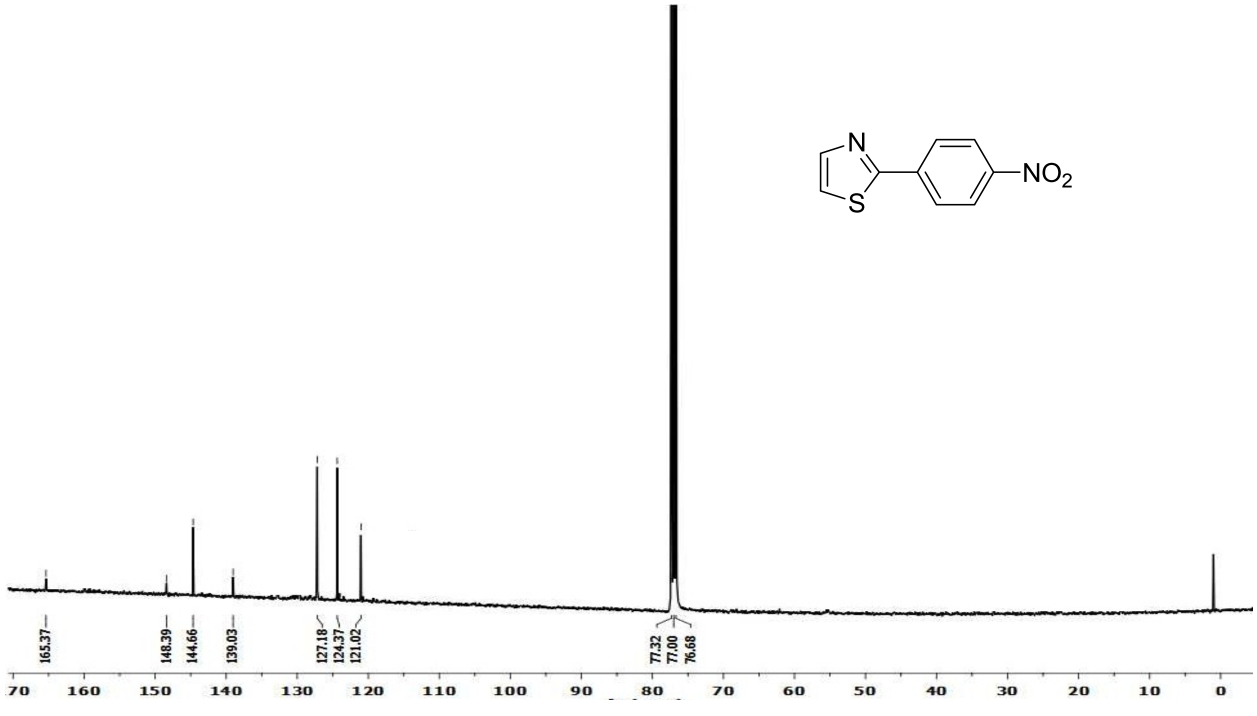

**Figure S8.** $^{1}$H NMR ($CDCl_3$) spectrum of 2-(4-methylphenyl)thiazole (**3c**):

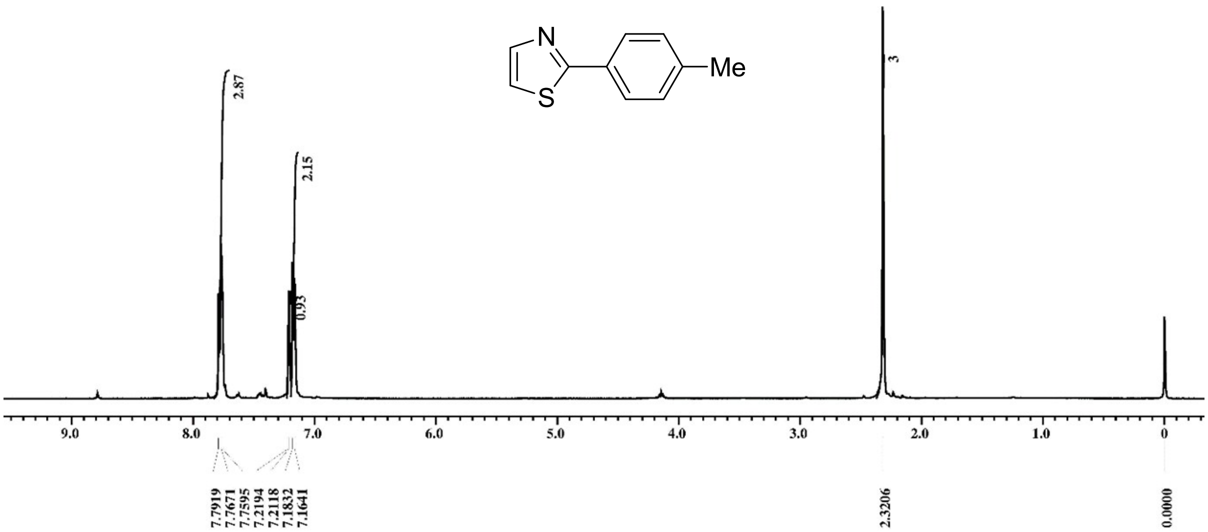


**Figure S9.** $^{13}$C NMR ($CDCl_3$) spectrum of 2-(4-methylphenyl)thiazole (**3c**):

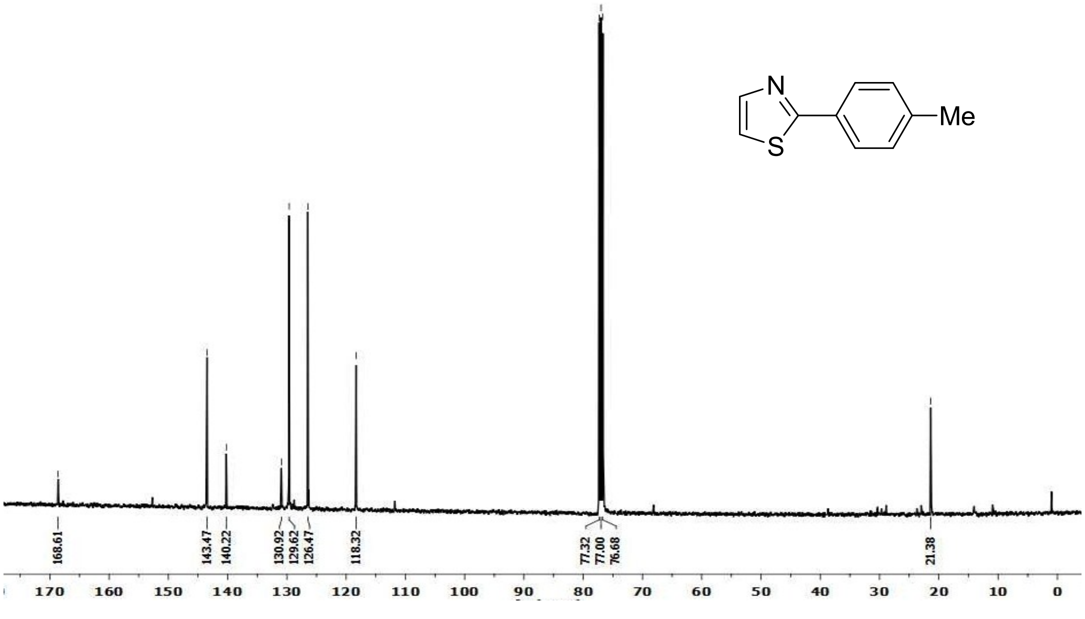

**Figure S10.** $^{1}$H NMR ($CDCl_3$) spectrum of 2-phenylthiazole (**3d**):

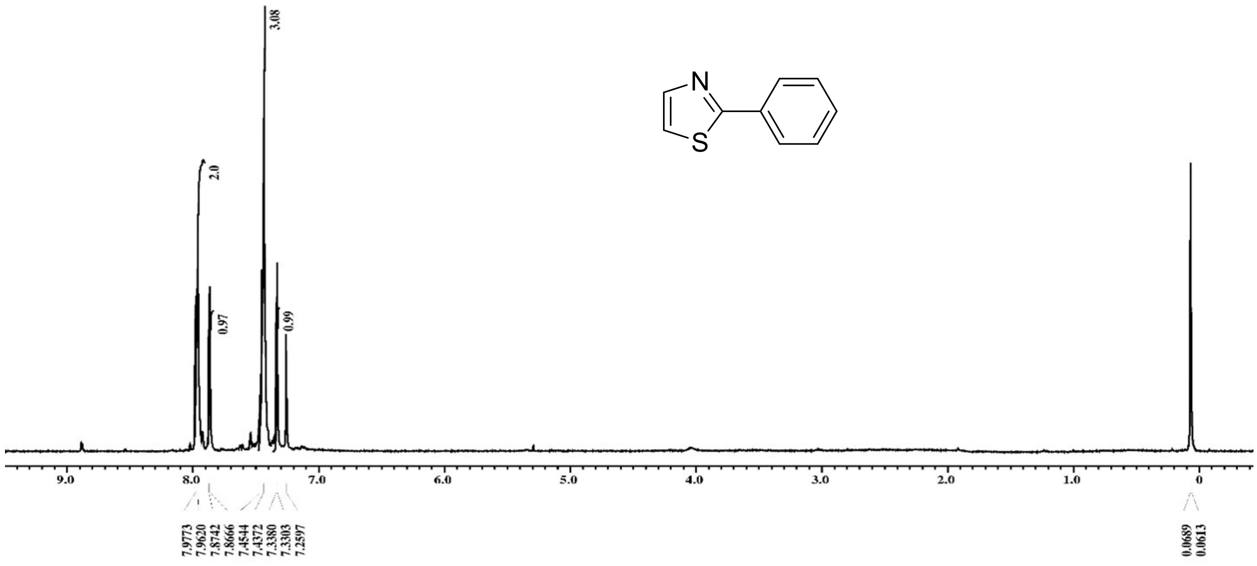


**Figure S11.** $^{13}$C NMR ($CDCl_3$) spectrum of 2-phenylthiazole (**3d**):

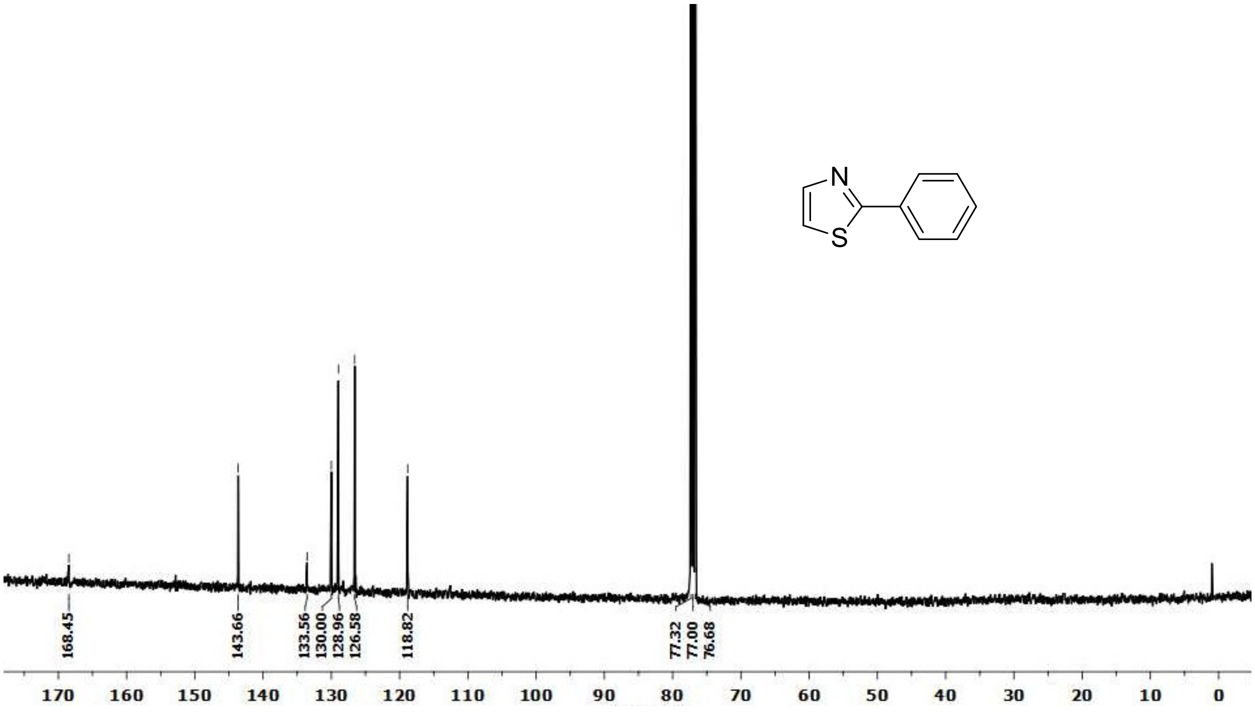

**Figure S12.** $^{1}$H NMR ($CDCl_3$) spectrum of 2-(4-methoxyphenyl)thiazole (**3e**):

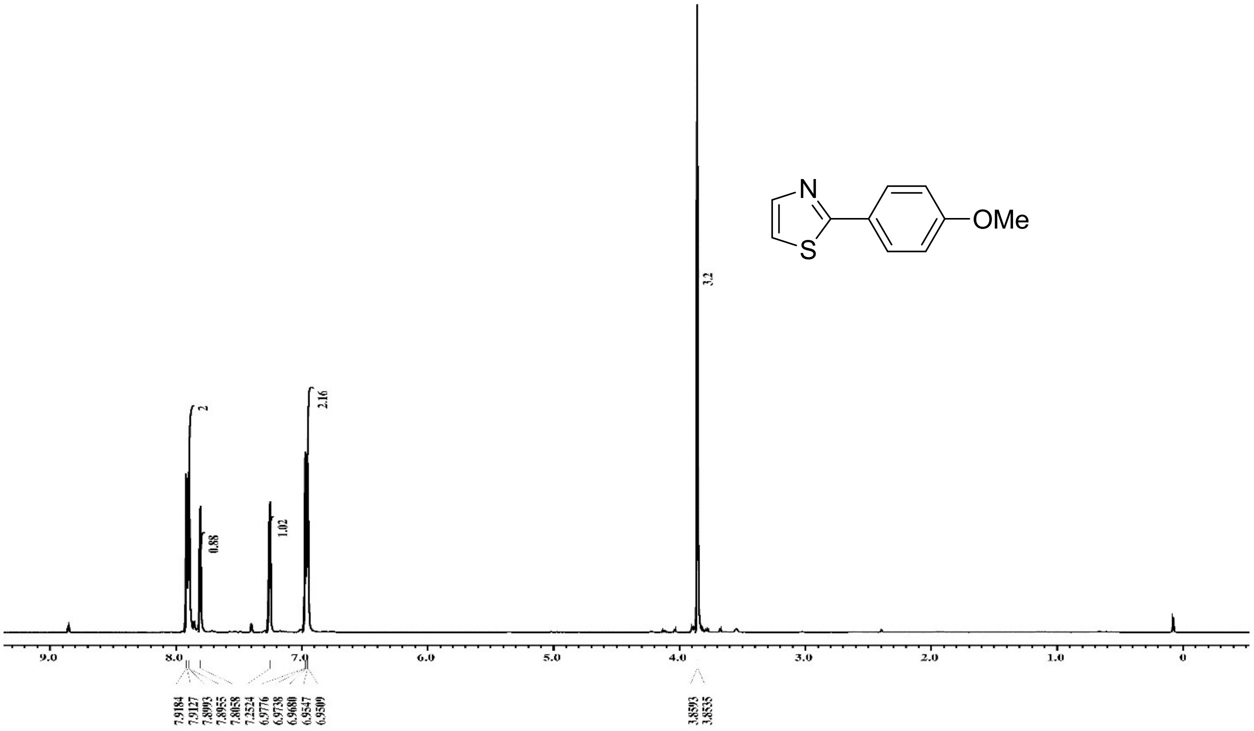


**Figure S13.** $^{13}$C NMR ($CDCl_3$) spectrum of 2-(4-methoxyphenyl)thiazole (**3e**):

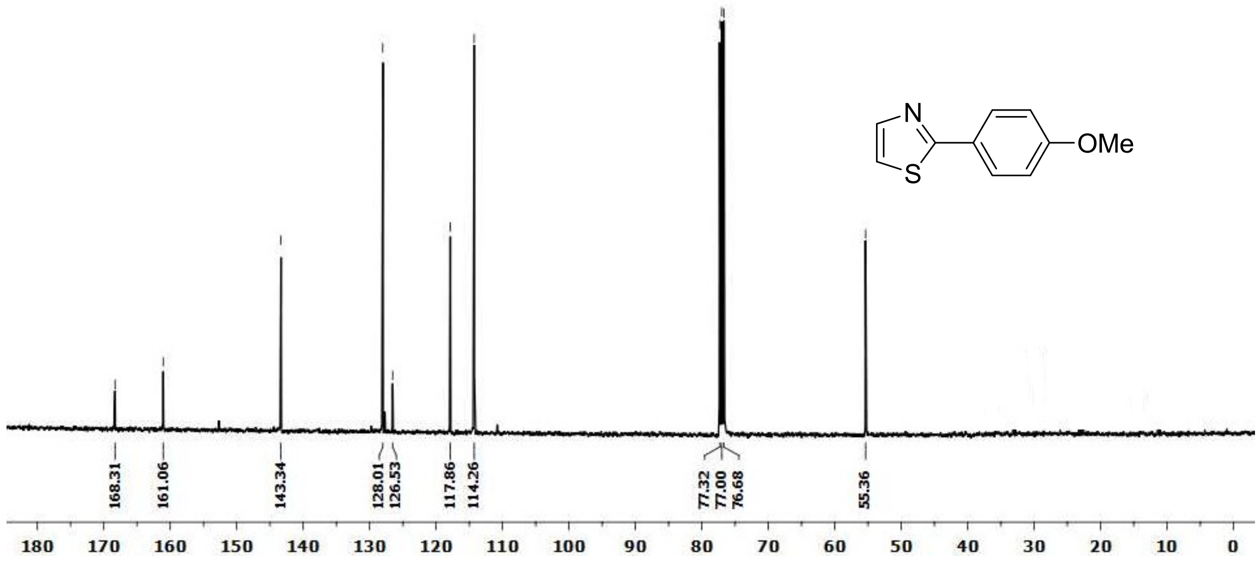

**Figure S14.** $^{1}H$ NMR ($CDCl_3$) spectrum of 2-(4-chlorophenyl)benzoxazole (**5a**):

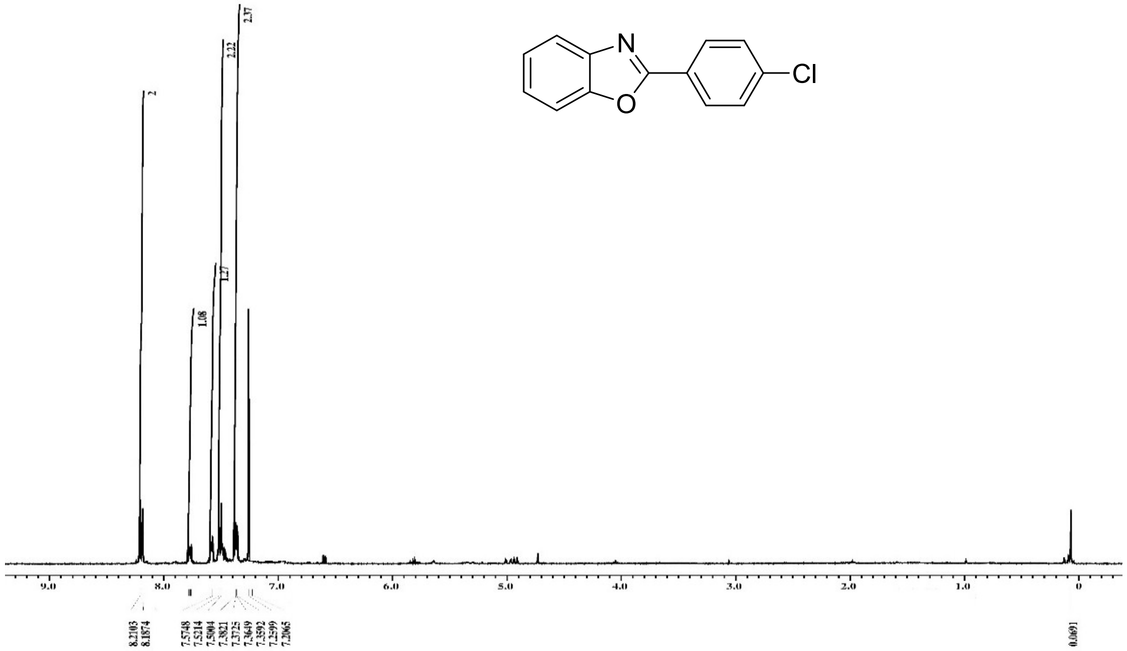


**Figure S15.** $^{13}C$ NMR ($CDCl_3$) spectrum of 2-(4-chlorophenyl)benzoxazole (**5a**):

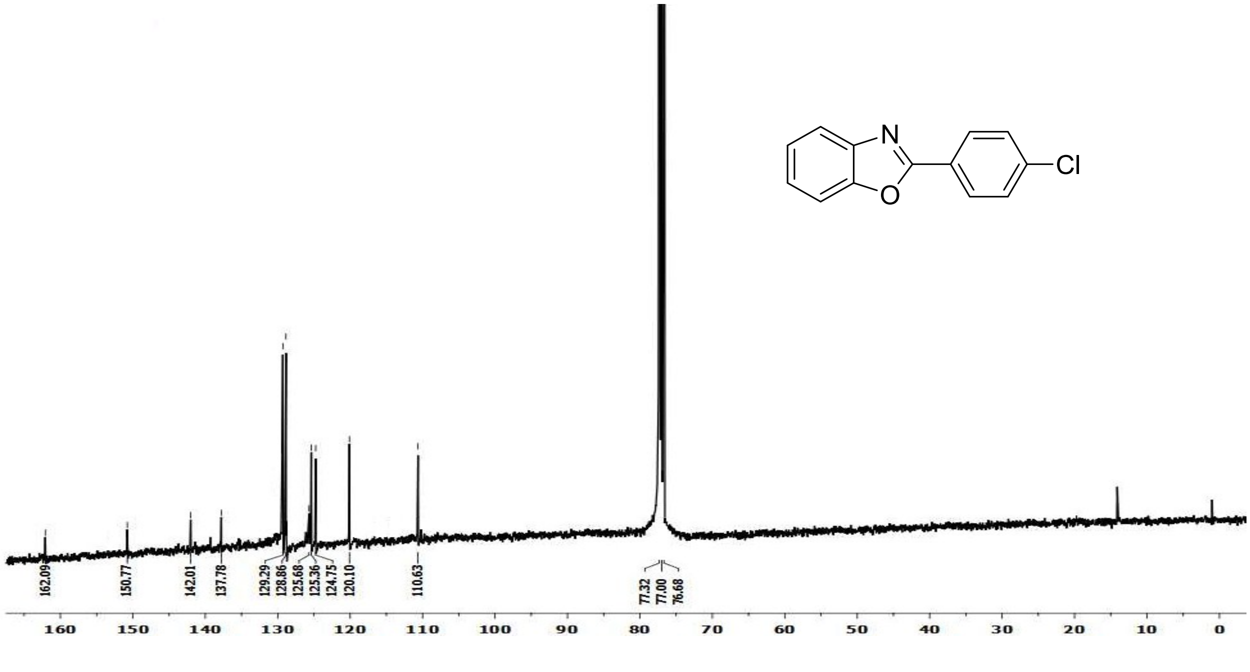

**Figure S16.** $^{1}$H NMR ($CDCl_3$) spectrum of 2-(4-methoxyphenyl)benzoxazole (**5e**):

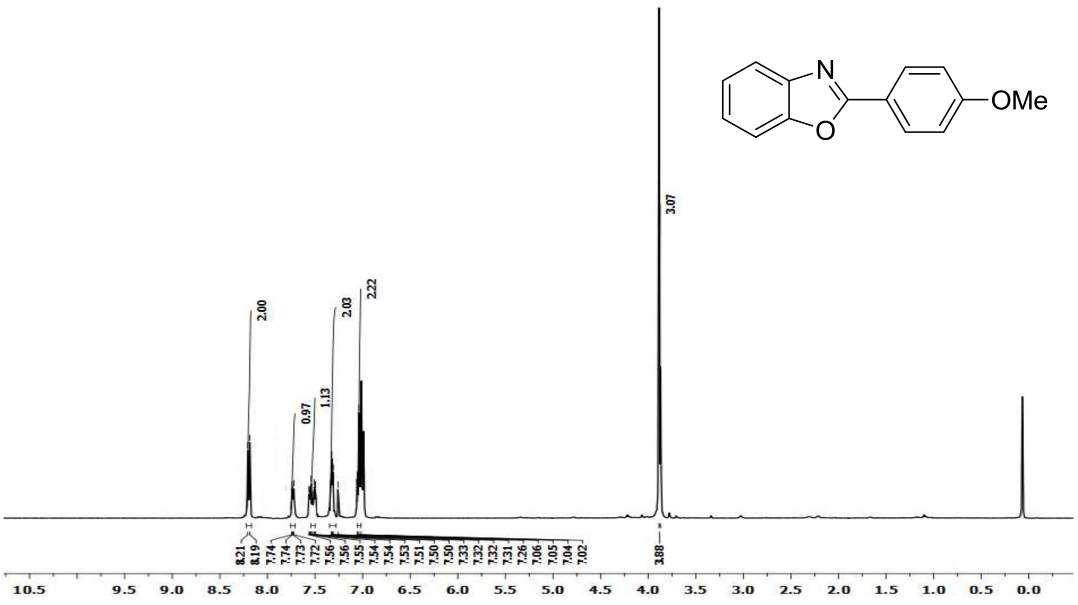


**Figure S17.** $^{13}$C NMR ($CDCl_3$) spectrum of 2-(4-methoxyphenyl)benzoxazole (**5e**):

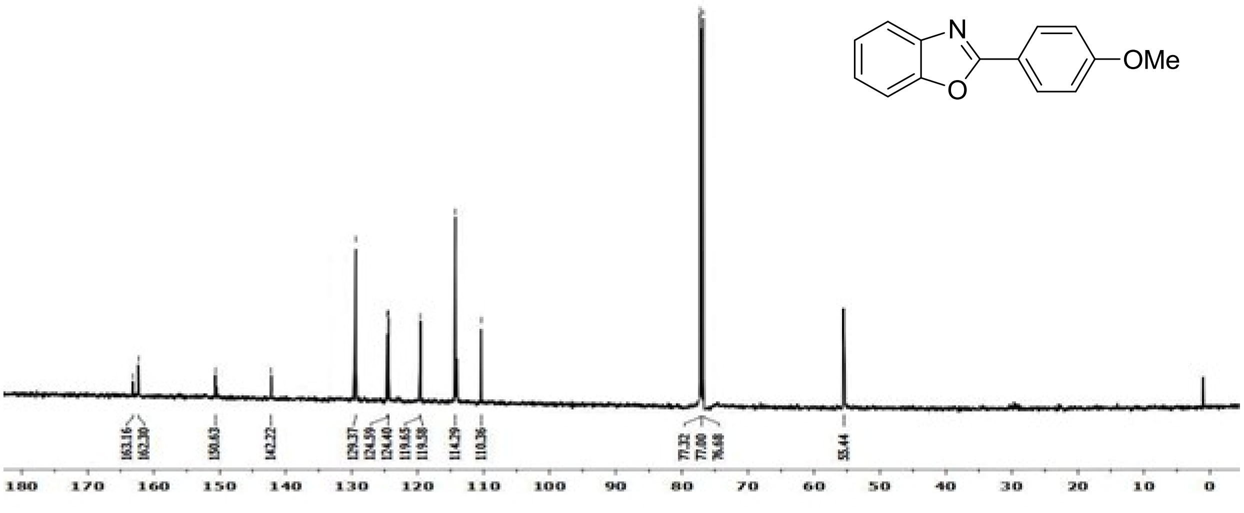

**Figure S18.** $^{1}H$ NMR ($CDCl_3$) spectrum of 2-(4-chlorophenyl)-5-nitrobenzoxazole (**6a**):

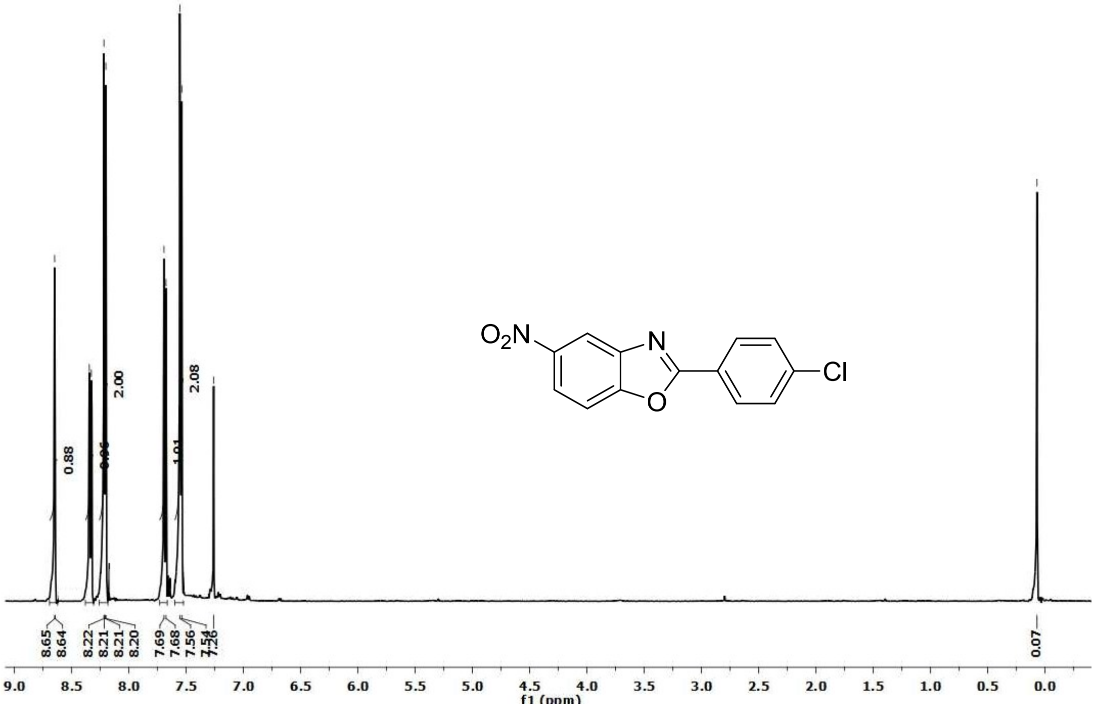


**Figure S19.** $^{13}C$ NMR ($CDCl_3$) spectrum of 2-(4-chlorophenyl)-5-nitrobenzoxazole (6**a**):

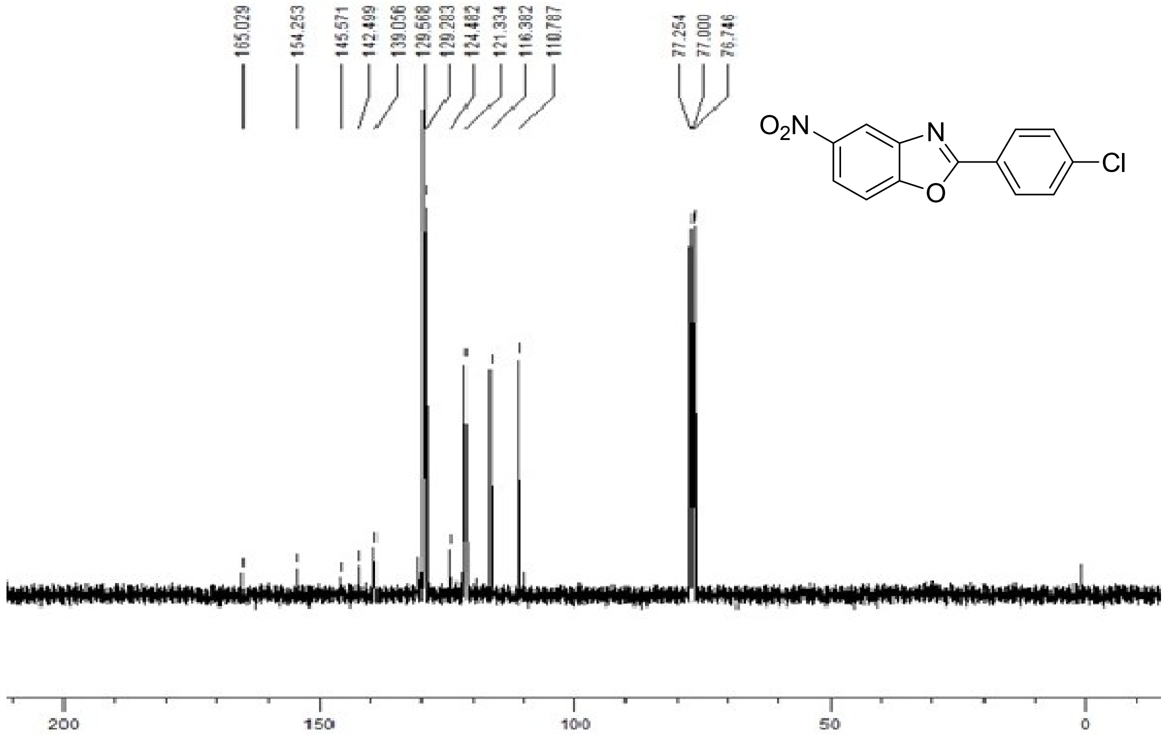

**Figure S20.** $^{1}$H NMR ($CDCl_3$) spectrum of 2-(4-methylphenyl)-5-nitrobenzoxazole (**6c**):

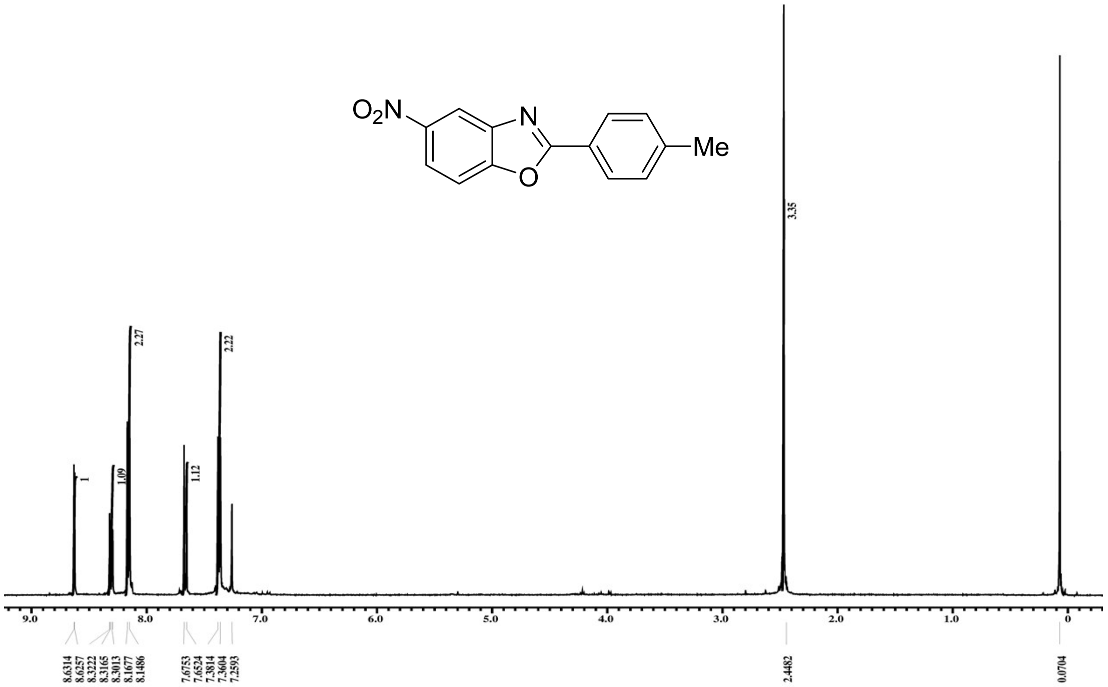


**Figure S21.** $^{13}$C NMR ($CDCl_3$) spectrum of 2-(4-methylphenyl)-5-nitrobenzoxazole (**6c**):

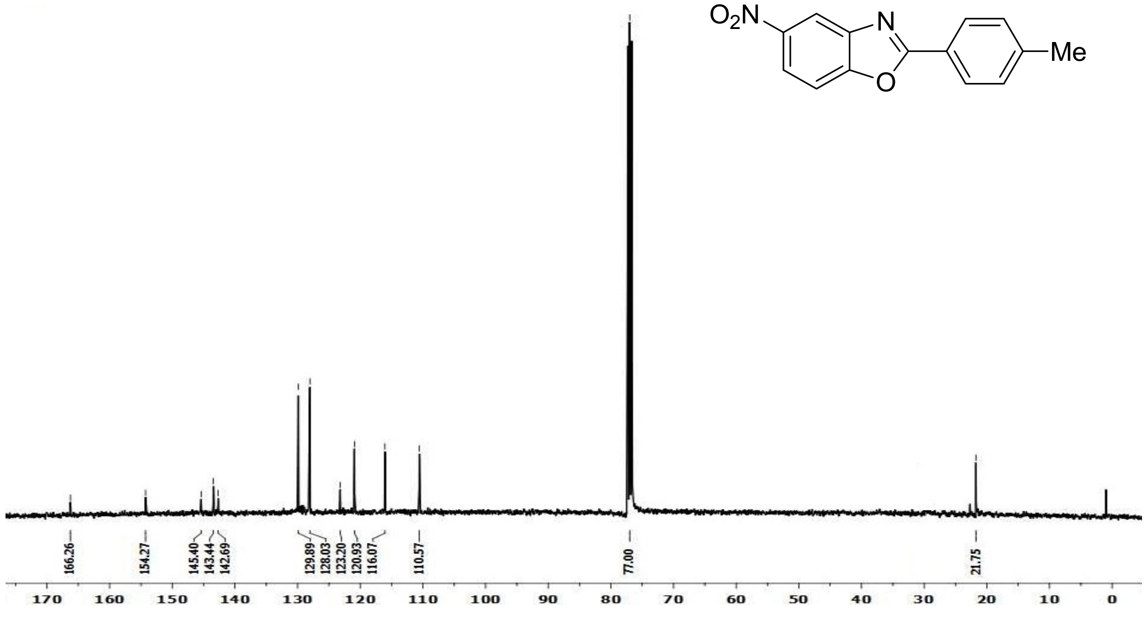

**Figure S22.** $^{1}$H NMR ($CDCl_3$) spectrum of 2-phenyl-5-nitrobenzoxazole (**6d**):

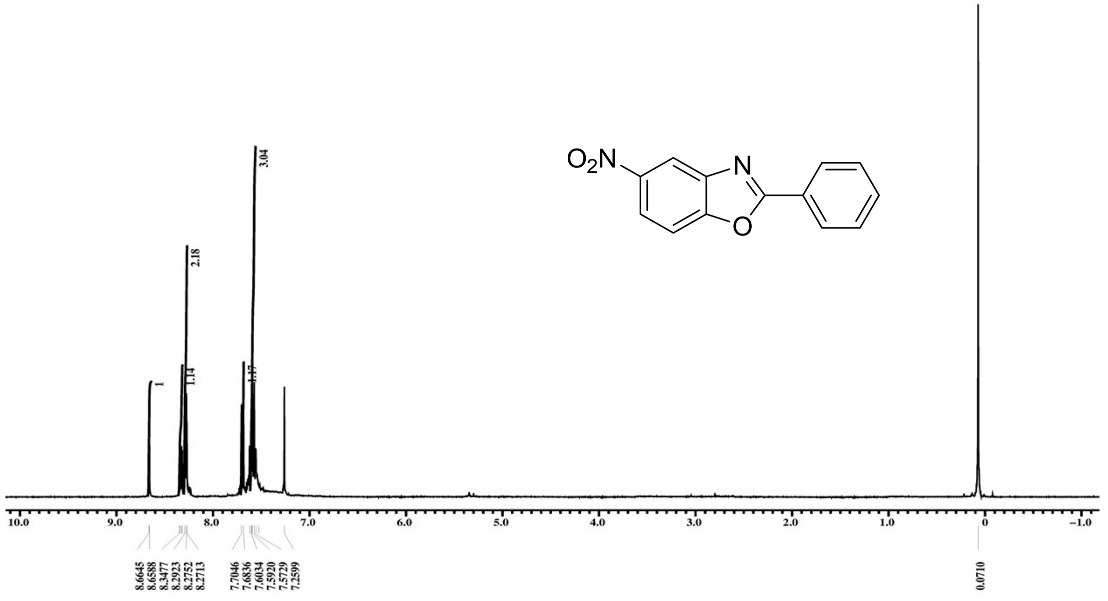


**Figure S23.** $^{13}$C NMR ($CDCl_3$) spectrum of 2-phenyl-5-nitrobenzoxazole (**6d**):

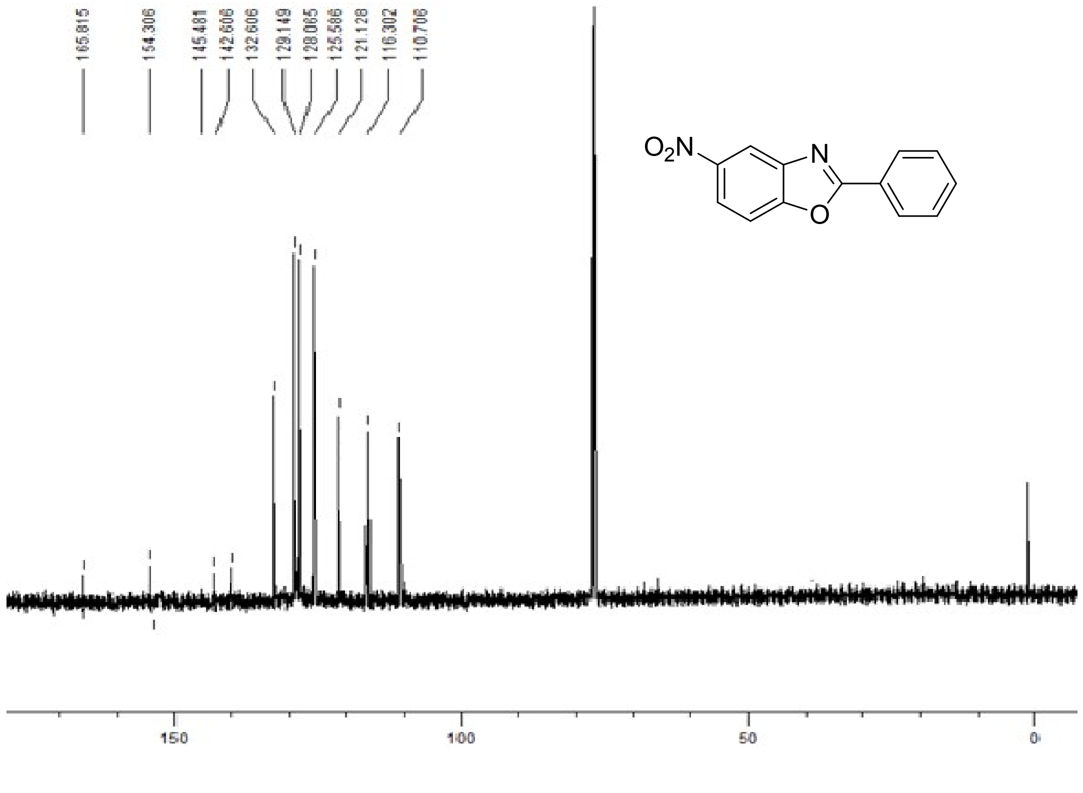

**Figure S24.** $^{1}$H NMR ($CDCl_3$) spectrum of 2-(4-methoxyphenyl)-5-nitrobenzoxazole (**6e**):

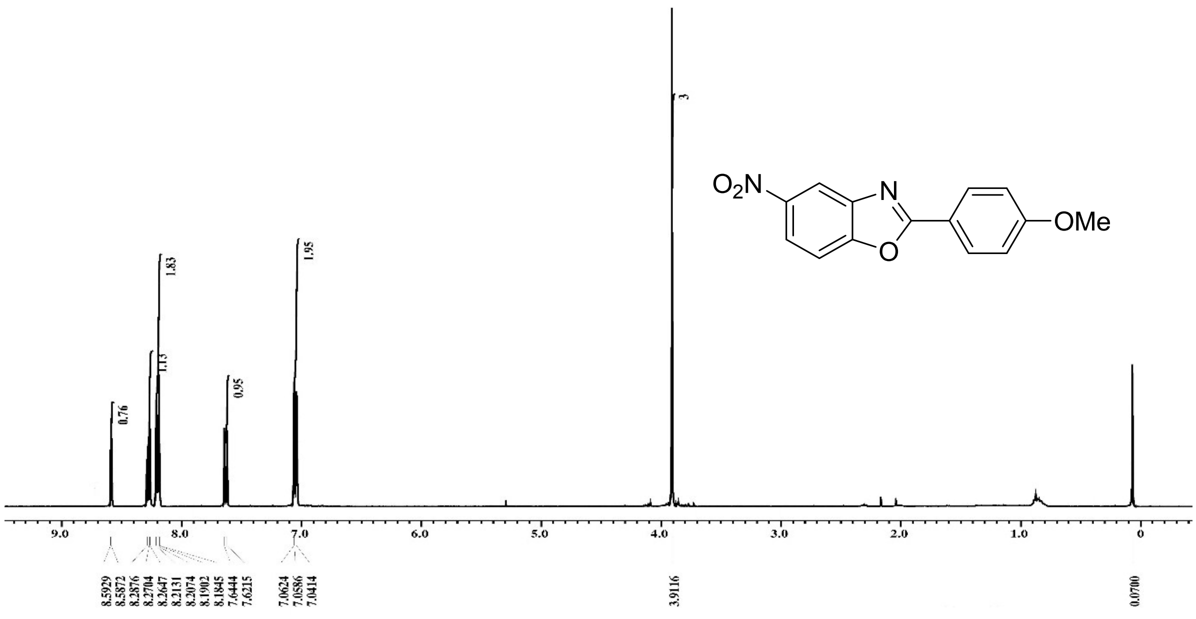


**Figure S25.** $^{13}$C NMR ($CDCl_3$) spectrum of 2-(4-methoxyphenyl)-5-nitrobenzoxazole (**6e**):

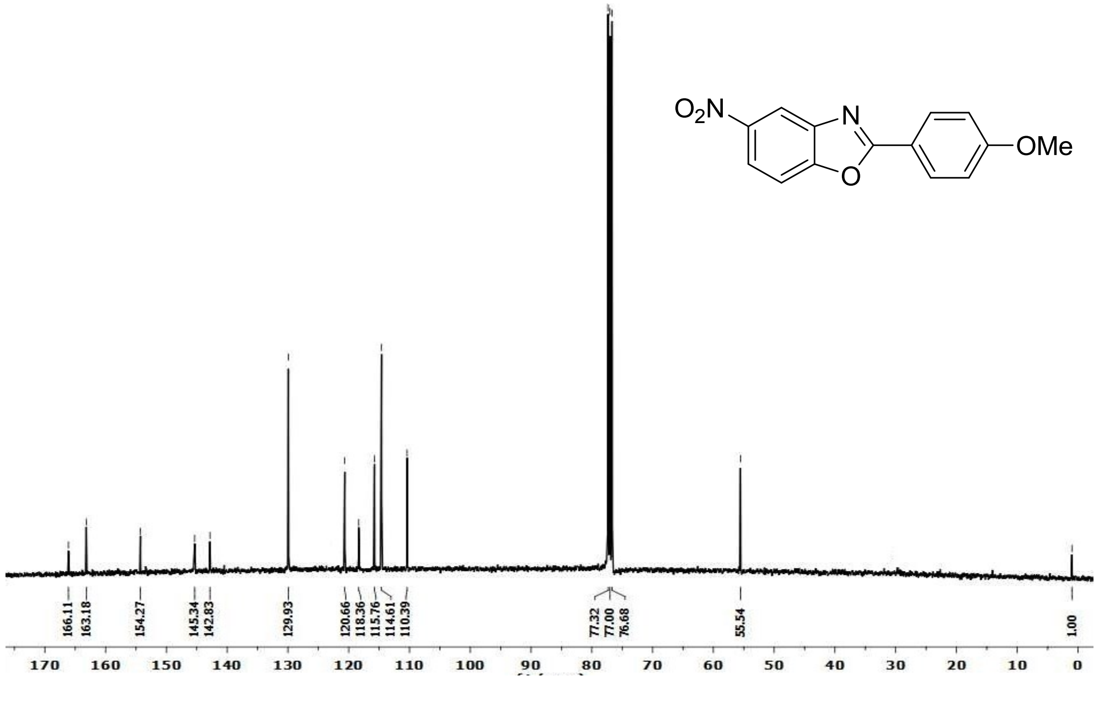

**Figure S26.** $^{1}H$ NMR ($CDCl_3$) spectrum of 2-(4-fluorophenyl)-5-nitrobenzoxazole (**6f**):

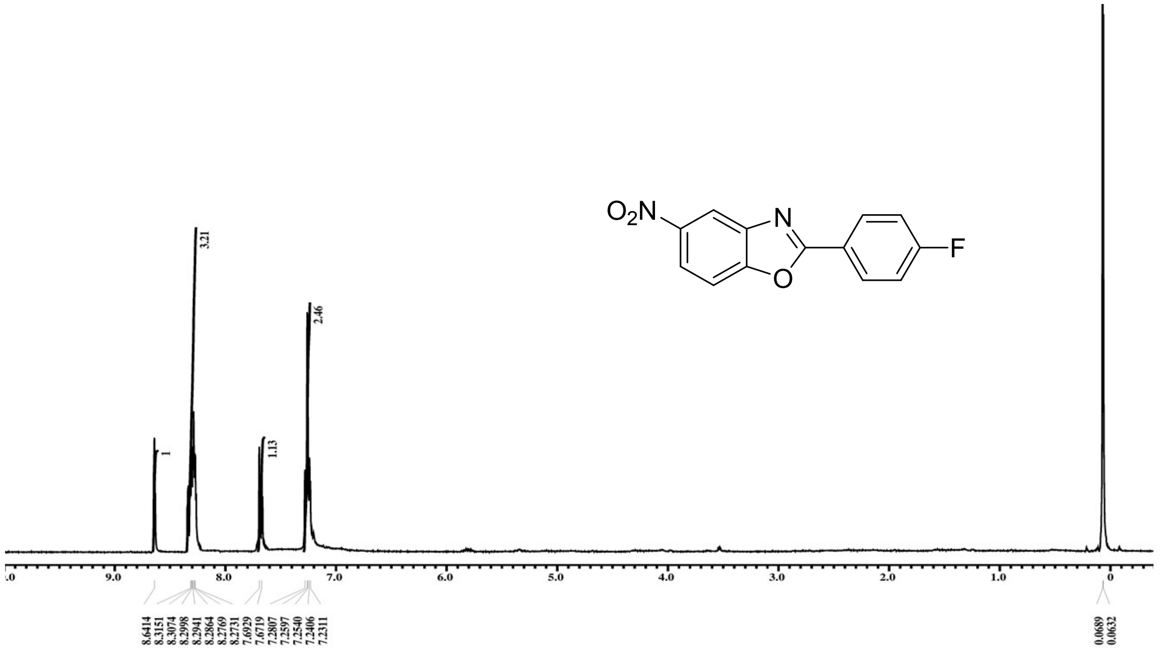


**Figure S27.** $^{13}C$ NMR ($CDCl_3$) spectrum of 2-(4-fluorophenyl)-5-nitrobenzoxazole (**6f**):

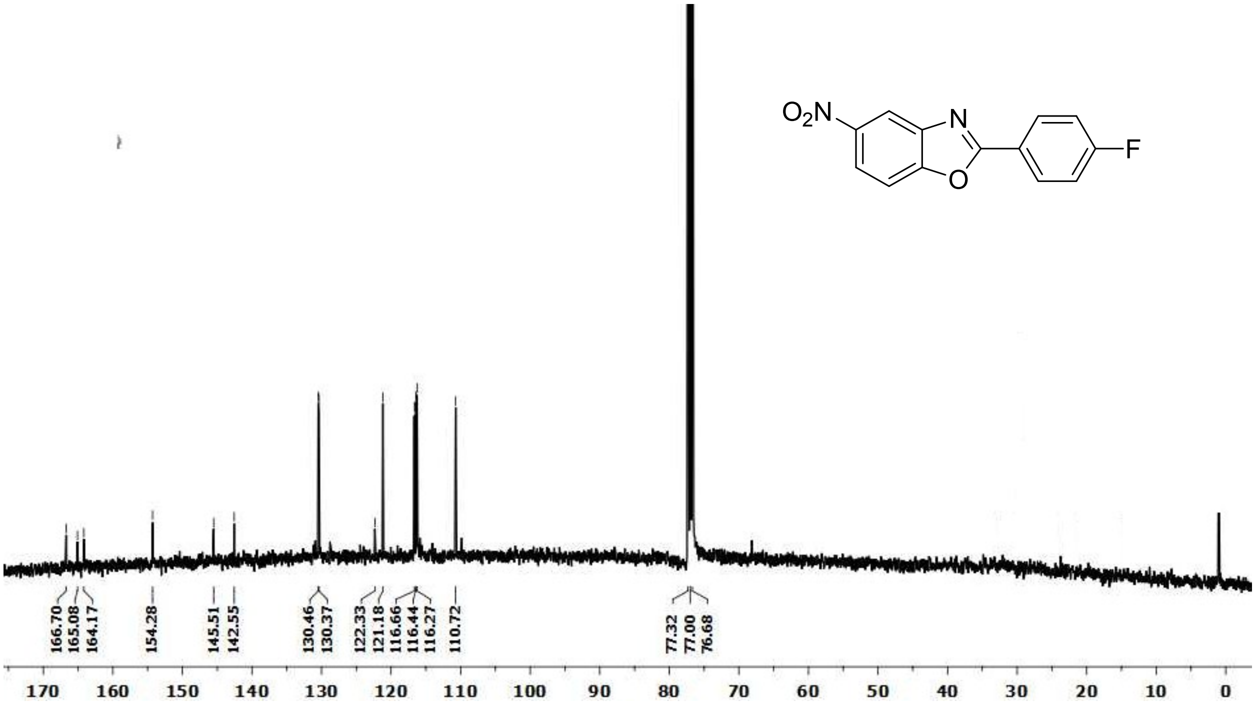

**Figure S28.** $^{1}$H NMR ($CDCl_3$) spectrum of 2-(4-chlorophenyl)-5-chlorobenzoxazole (**7a**):

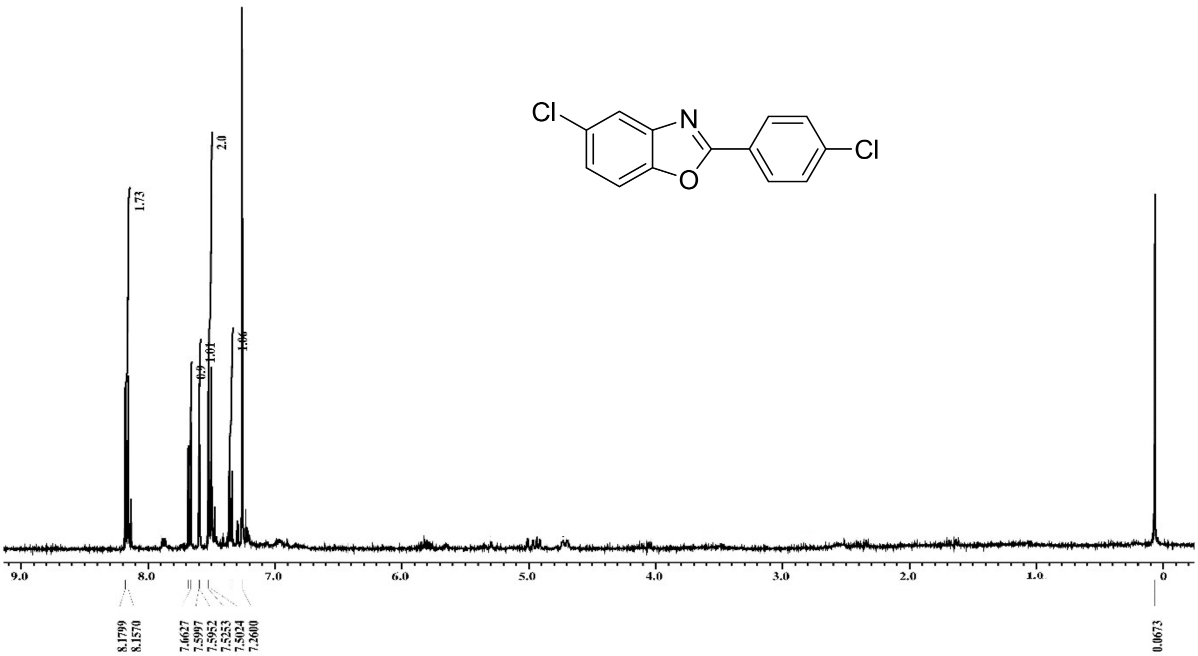


**Figure S29.** $^{13}$C NMR ($CDCl_3$) spectrum of 2-(4-chlorophenyl)-5-chlorobenzoxazole (**7a**):

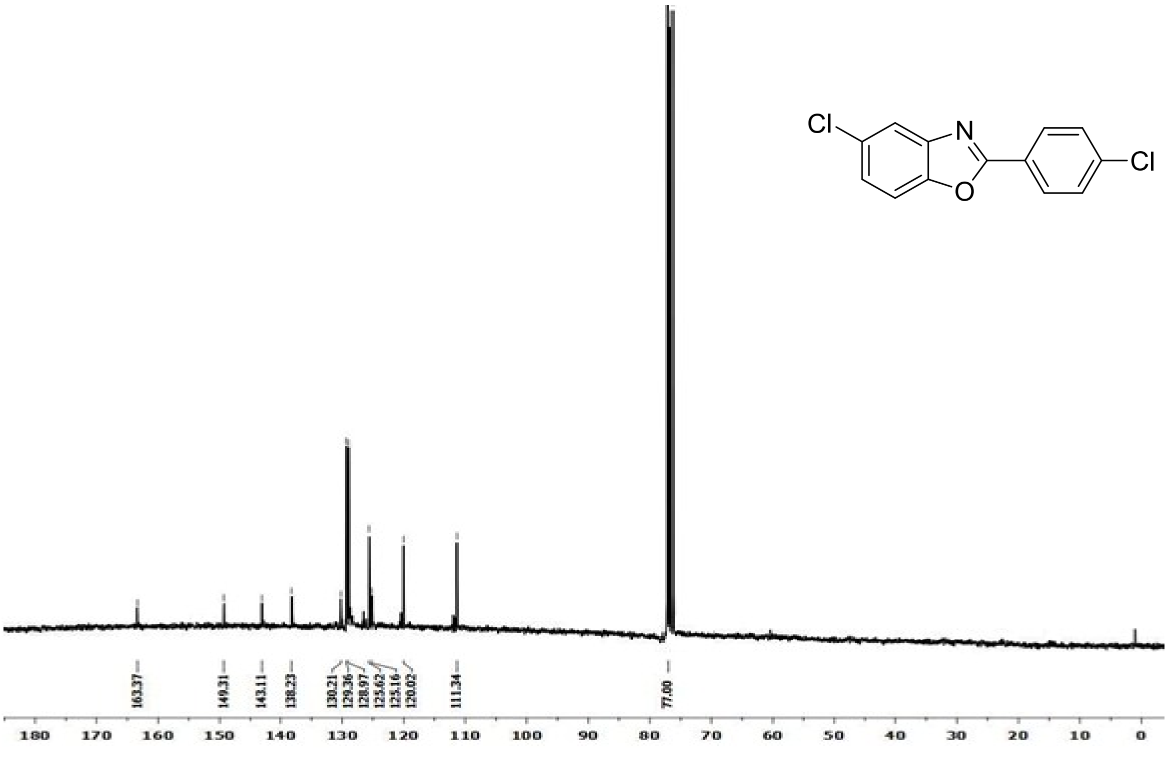

**Figure S30.** $^{1}$H NMR ($CDCl_3$) spectrum of 2-(4-nitrophenyl)-5-chlorobenzoxazole (**7b):**

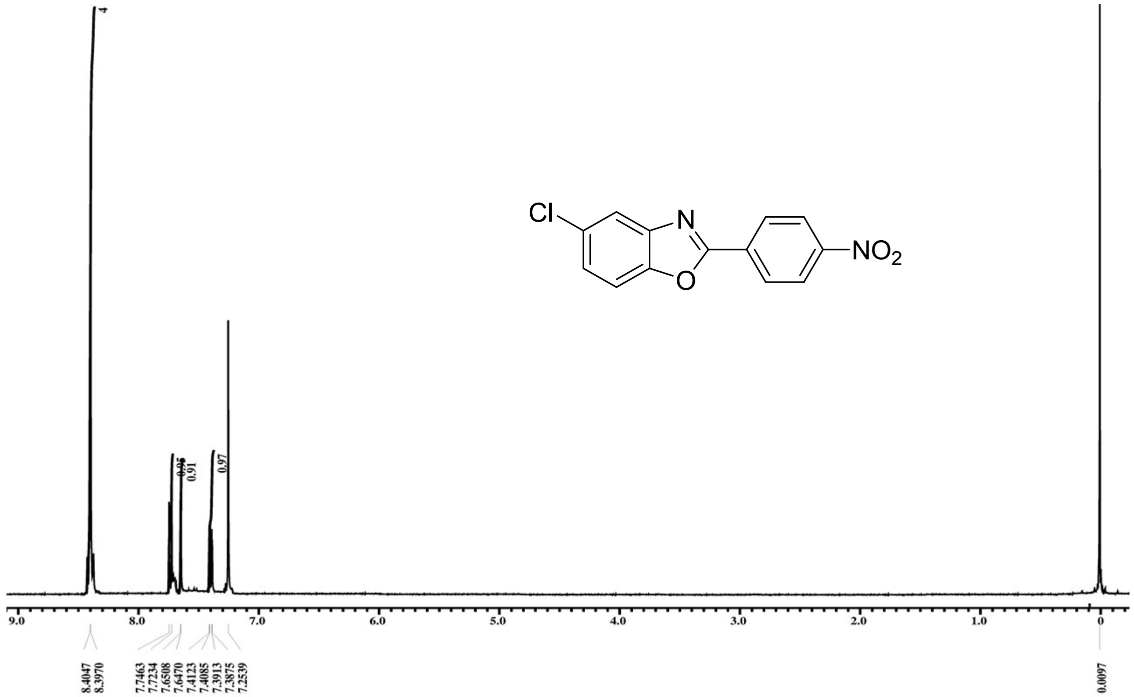


**Figure S31.** $^{13}$C NMR ($CDCl_3$) spectrum of 2-(4-nitrophenyl)-5-chlorobenzoxazole (**7b**):

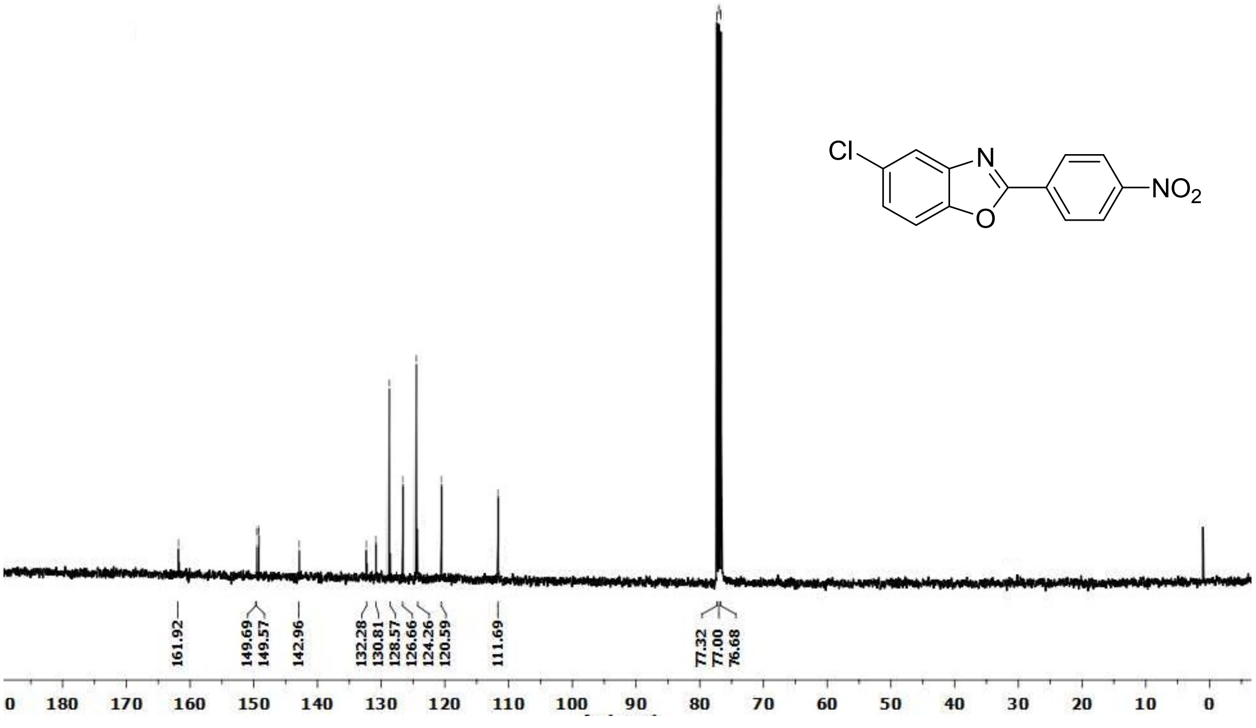

**Figure S32.** $^{1}$H NMR ($CDCl_3$) spectrum of 2-(4-chlorophenyl)-5-methylbenzoxazole (**8a**):

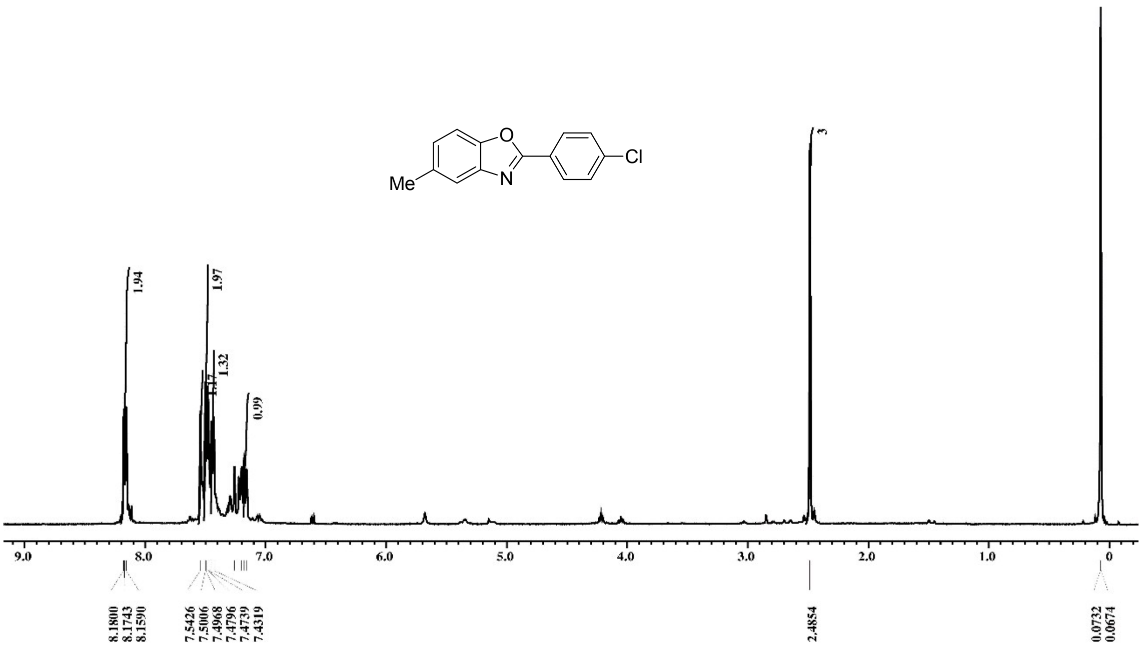


**Figure S33.** $^{13}$C NMR ($CDCl_3$) spectrum of 2-(4-chlorophenyl)-5-methylbenzoxazole (**8a**):

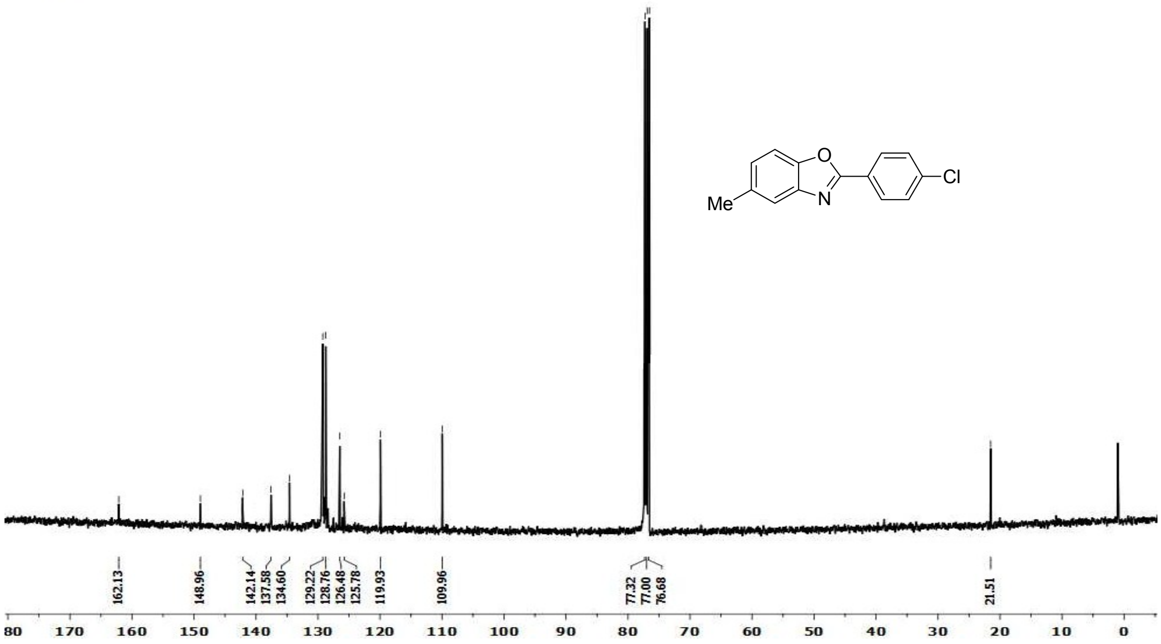

**Figure S34.** $^{1}$H NMR ($CDCl_3$) spectrum of 5-methyl-2-(4-nitrophenyl)benzoxazole (**8b**):

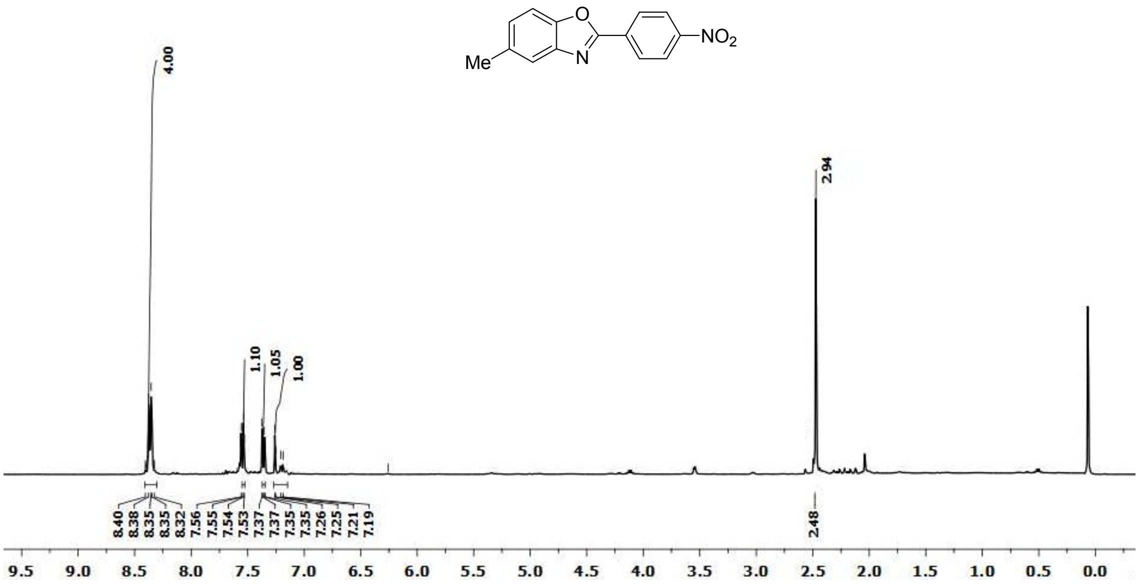


**Figure S35.** $^{13}$C NMR ($CDCl_3$) spectrum of 5-methyl-2-(4-nitrophenyl)benzoxazole (**8b**):

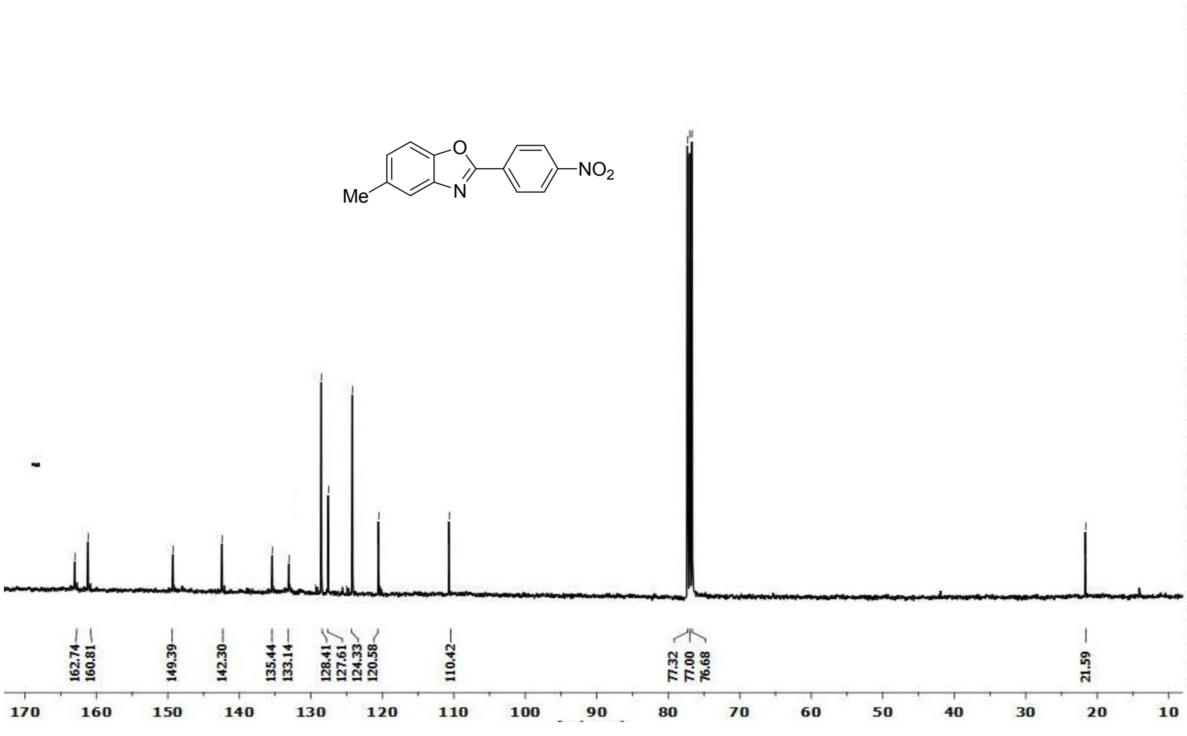

**Figure S36.** $^{1}H$ NMR ($CDCl_3$) spectrum of 2-(4-chlorophenyl)thiophene (**10a**):

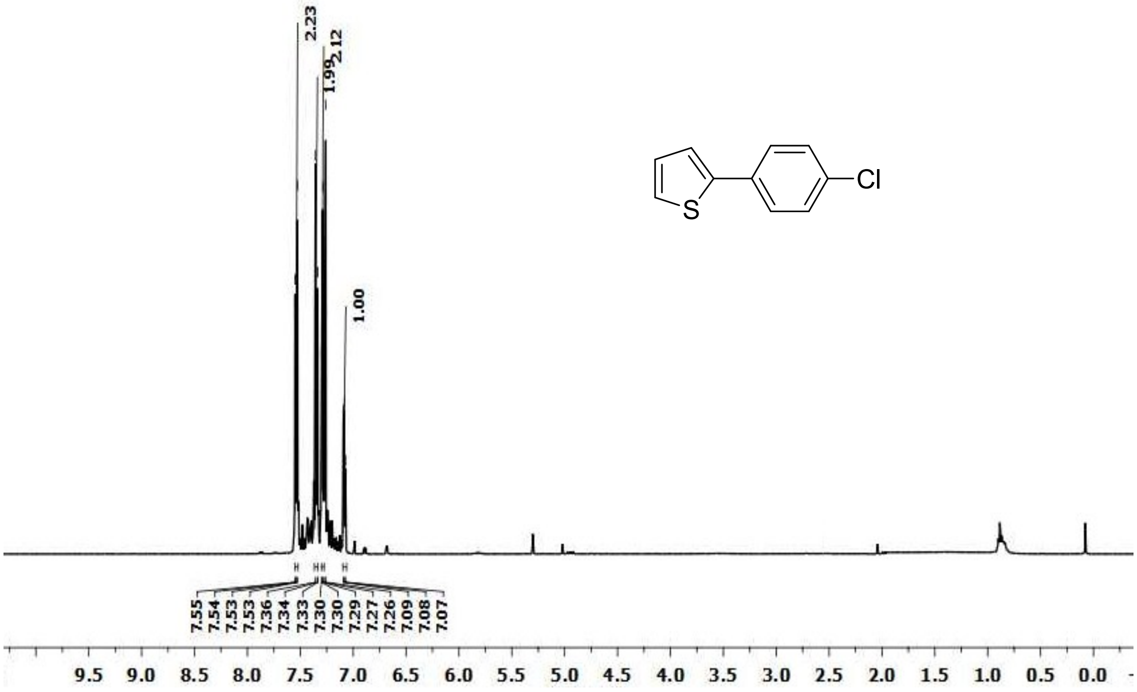


**Figure S37.** $^{13}C$ NMR ($CDCl_3$) spectrum of 2-(4-chlorophenyl)thiophene (**10a**):

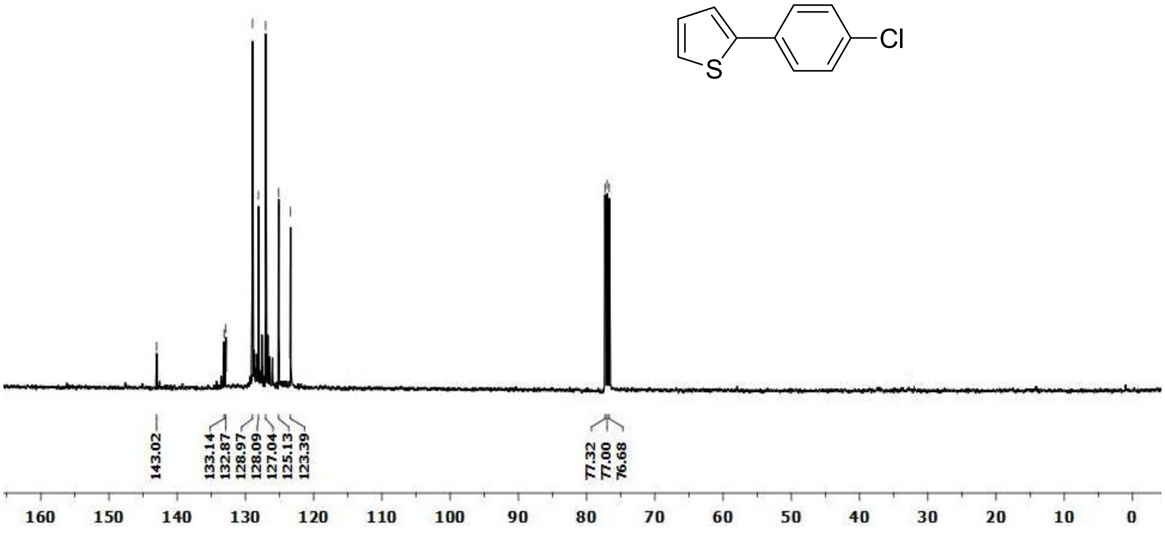

**Figure S38.** $^{1}H$ NMR ($CDCl_3$) spectrum of 2-(4-nitrophenyl)thiophene (**10b**):

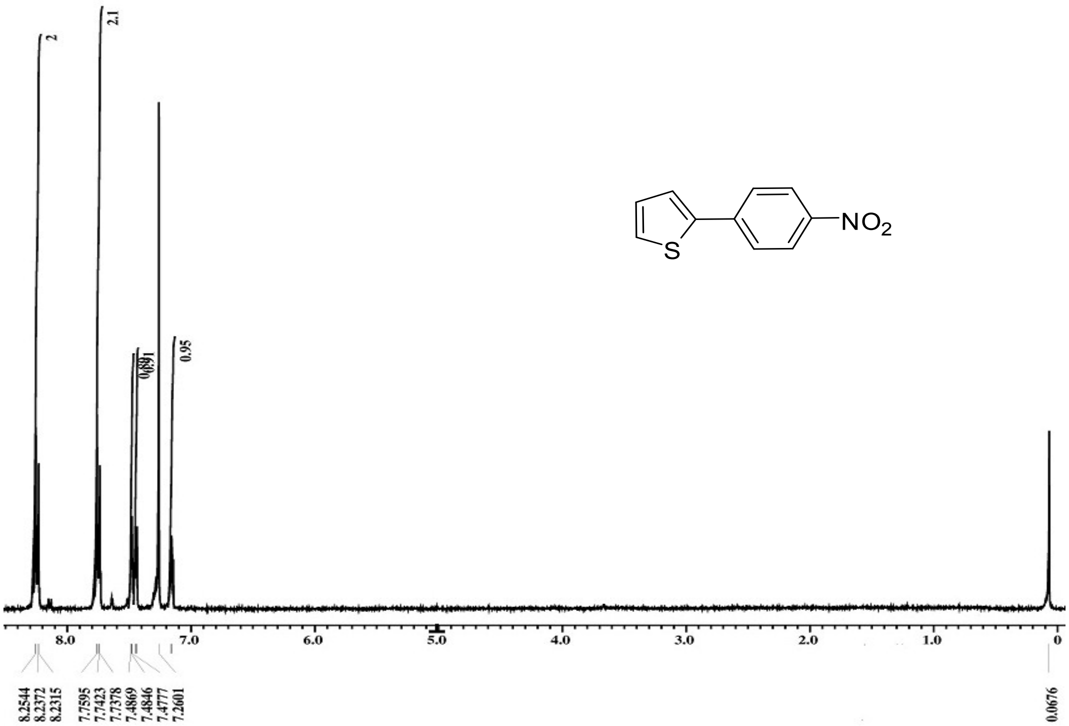


**Figure S39.** $^{13}C$ NMR ($CDCl_3$) spectrum of 2-(4-nitrophenyl)thiophene (**10b**):

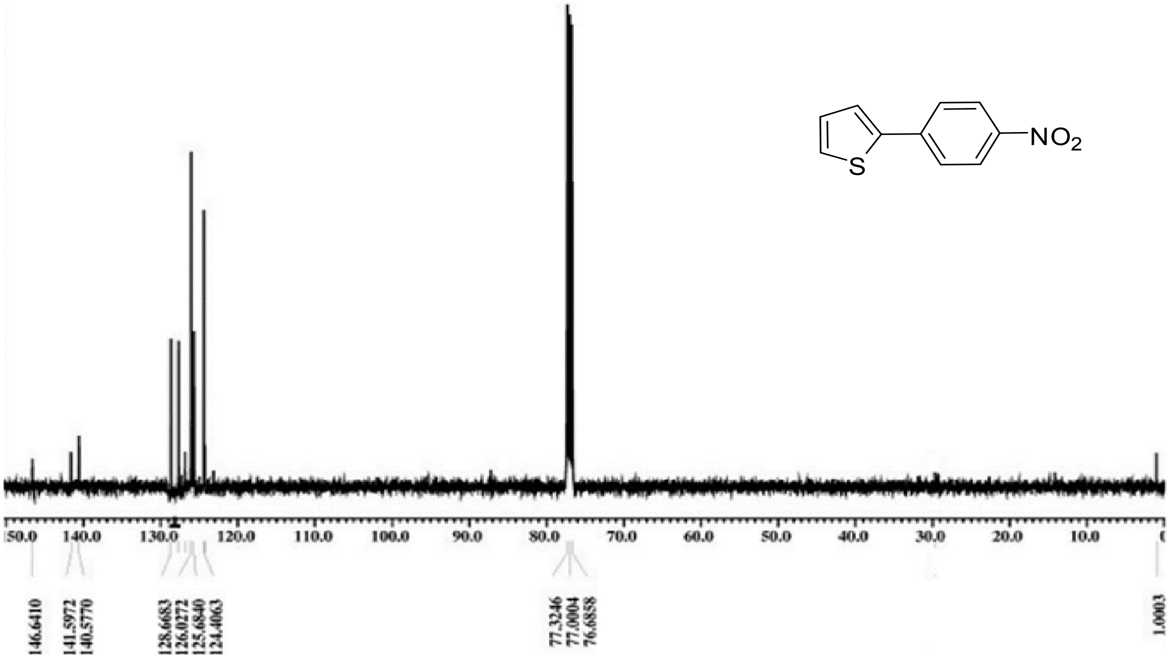

**Figure S40.** $^{1}$H NMR ($CDCl_3$) spectrum of 2-phenylthiophene (**10d**):

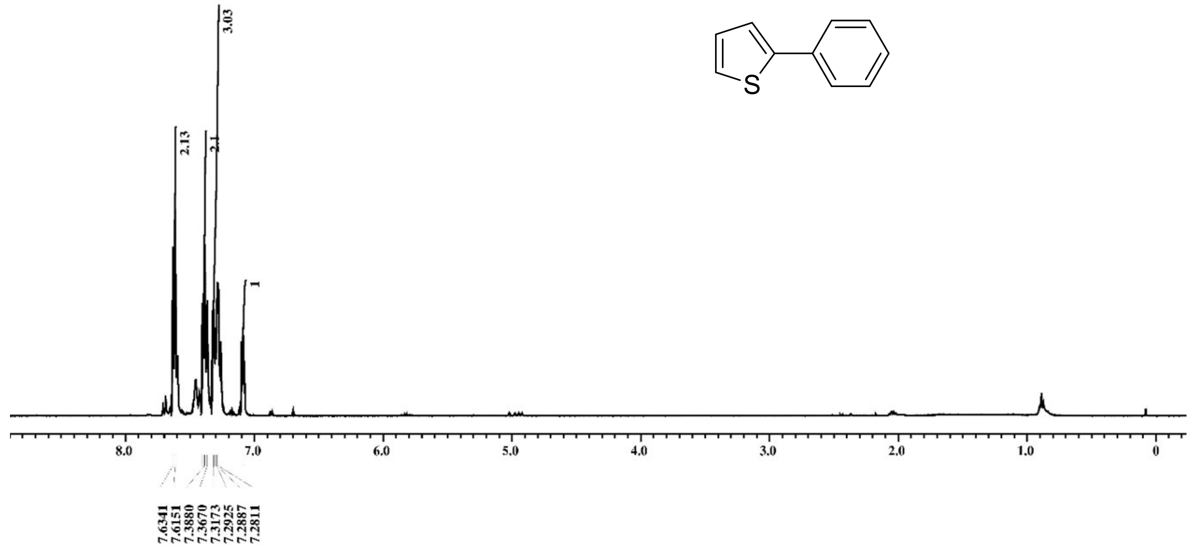


**Figure S41.** $^{13}$C NMR ($CDCl_3$) spectrum of 2-phenylthiophene (**10d**):

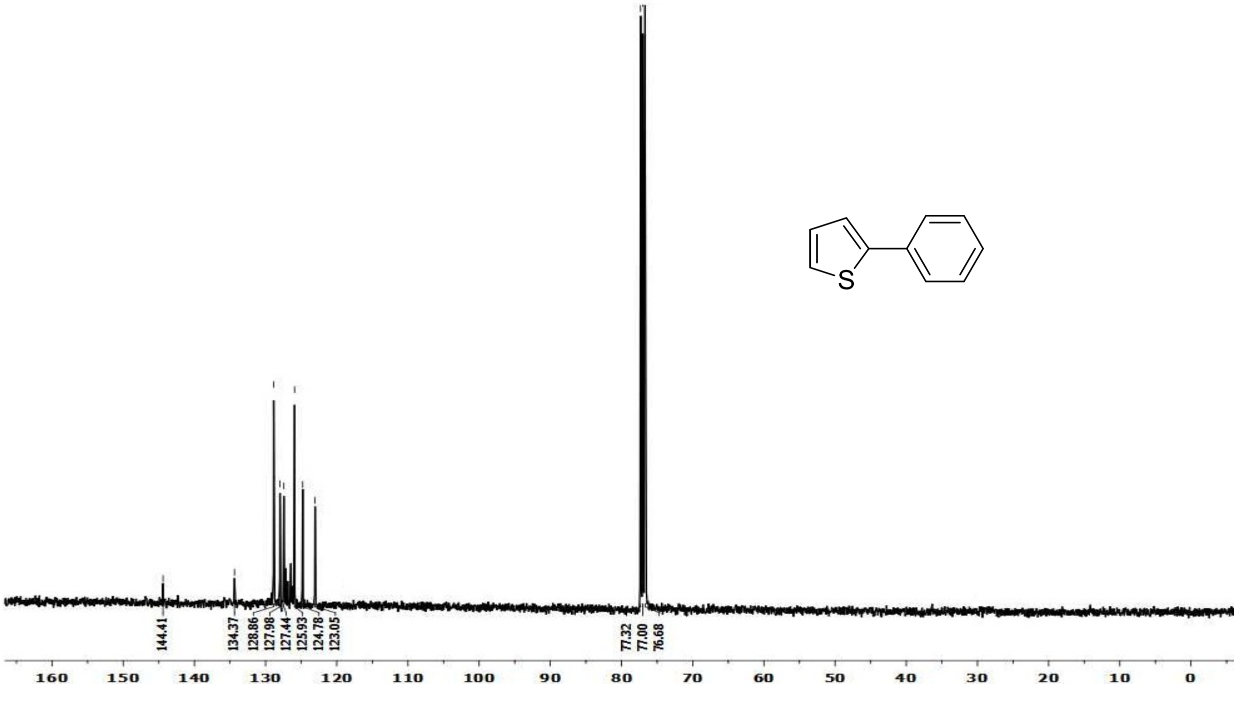

**Figure S42.** $^{1}$H NMR ($CDCl_3$) spectrum of 2-(4-methoxyphenyl)thiophene (**10e**):

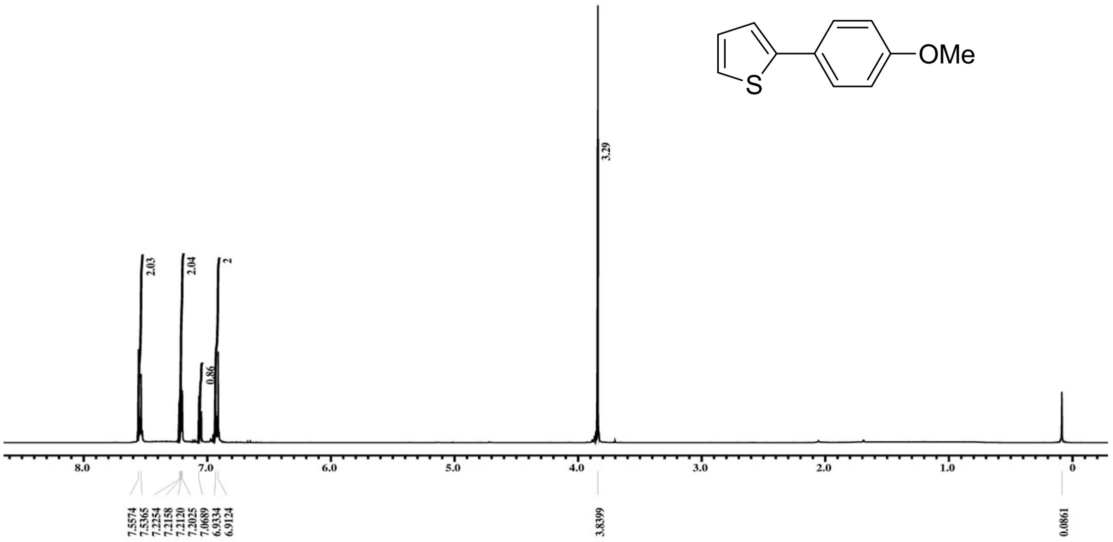


**Figure S43.** $^{13}$C NMR ($CDCl_3$) spectrum of 2-(4-methoxyphenyl)thiophene (**10e**):

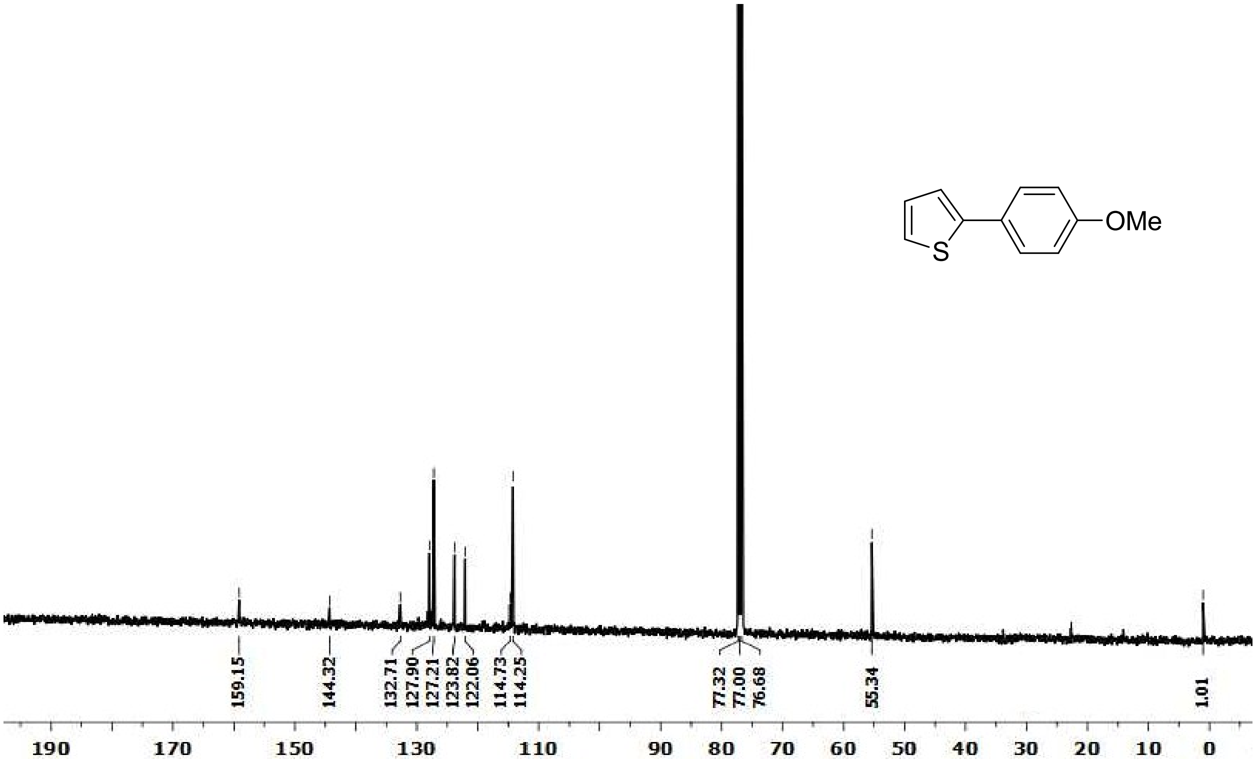

**Figure S44.** $^{1}H$ NMR ($CDCl_3$) spectrum of 2-(4-cyanophenyl)thiophene (**10g**):

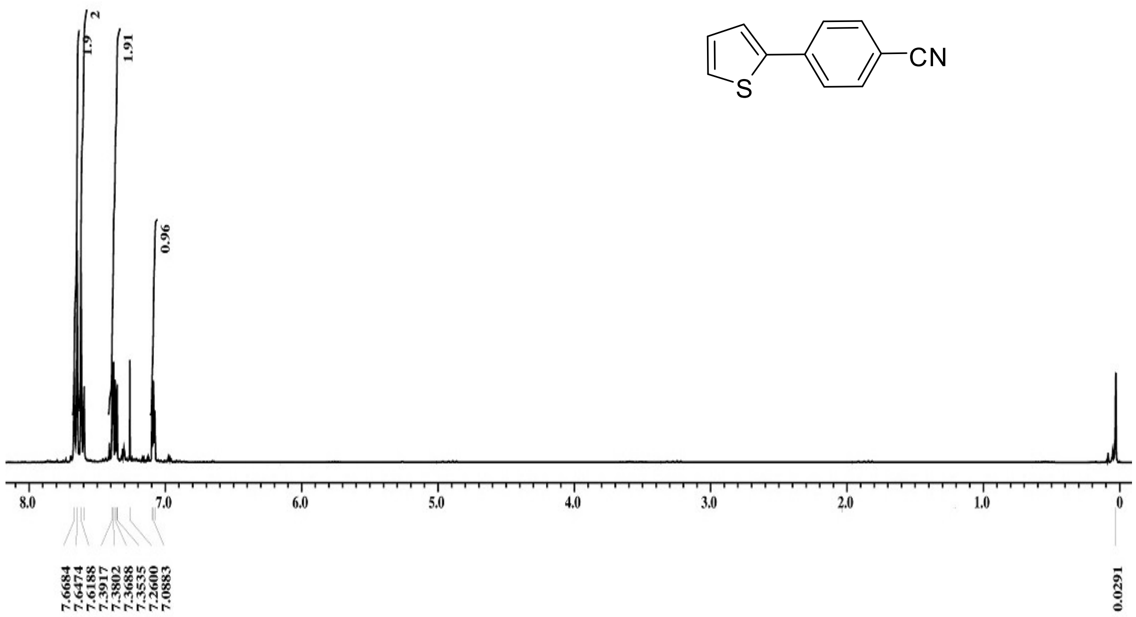


**Figure S45.** $^{13}C$ NMR ($CDCl_3$) spectrum of 2-(4-cyanophenyl)thiophene (**10g**):

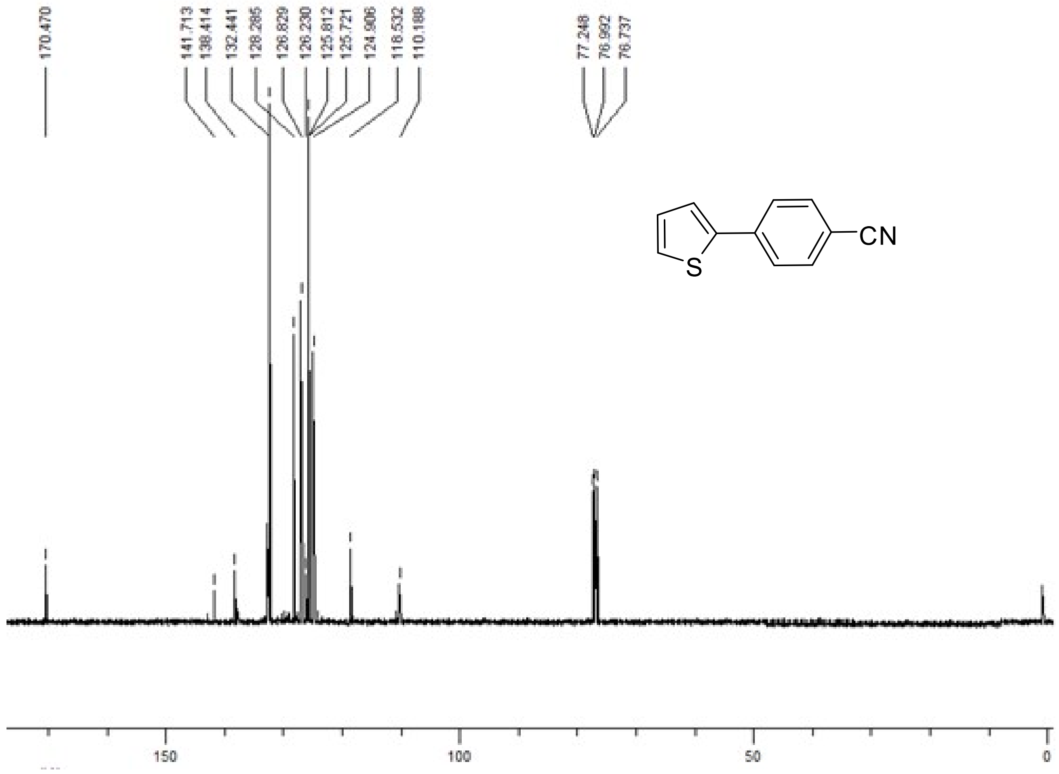

**Figure S46.** $^{1}$H NMR ($CDCl_3$) spectrum of 2-(2-nitrophenyl)thiophene (**10h**):

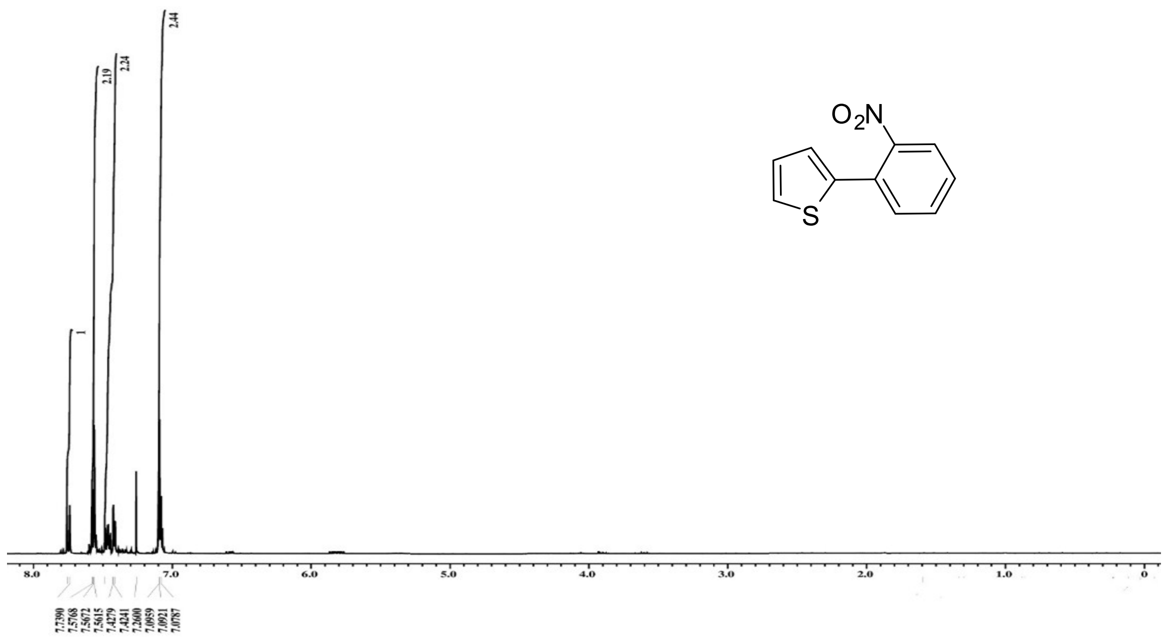


**Figure S47.** $^{13}$C NMR ($CDCl_3$) spectrum of 2-(2-nitrophenyl)thiophene (**10h**):

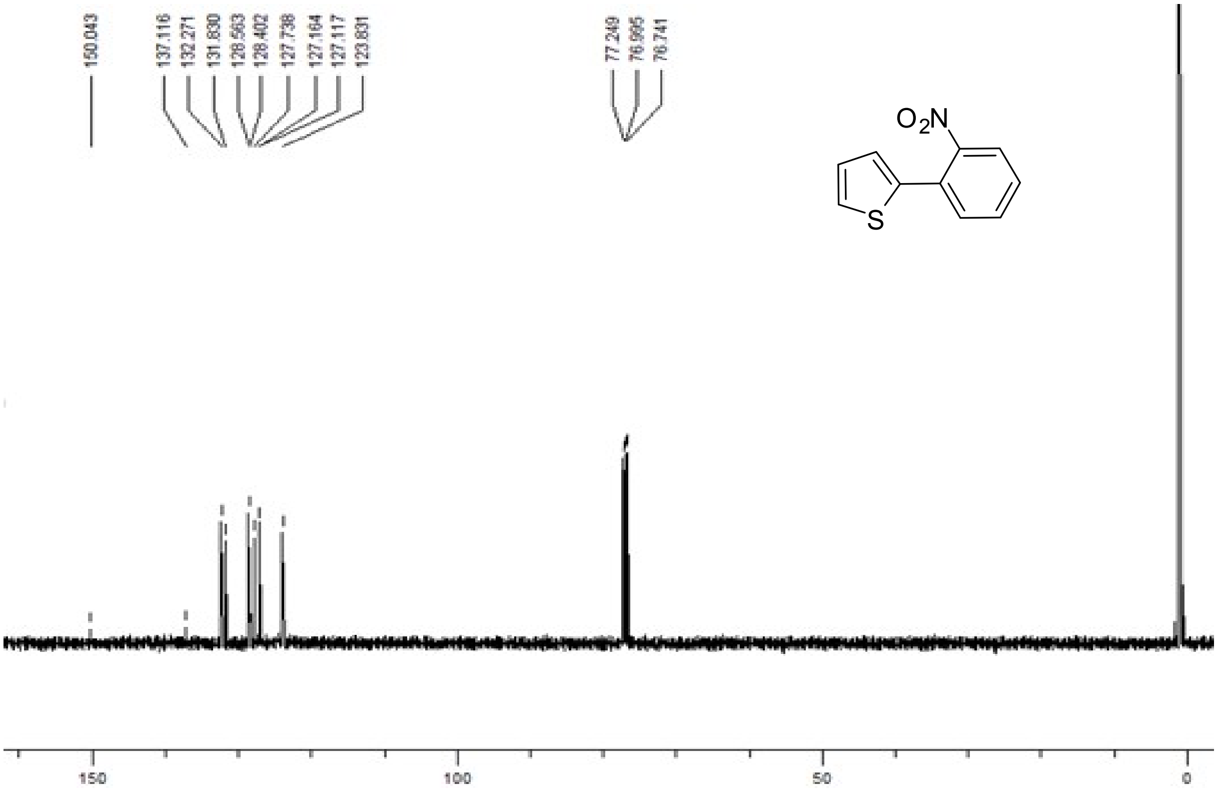

**Figure S48.** $^{1}H$ NMR ($CDCl_3$) spectrum of 2-(4-chlorophenyl)furan (**11a**):

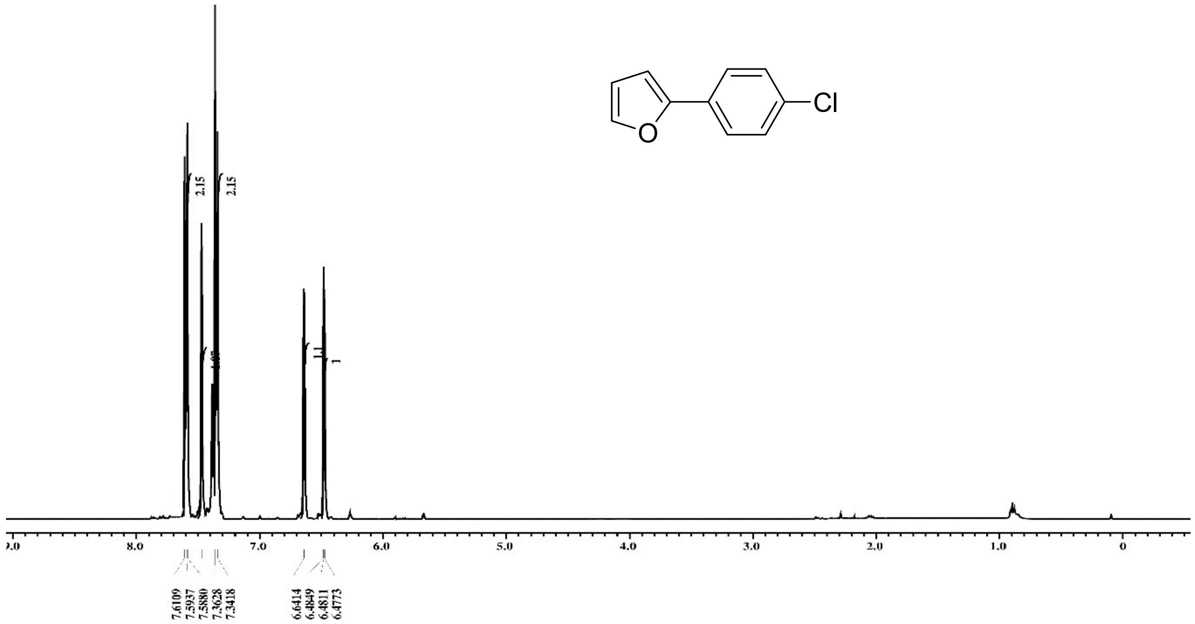


**Figure S49.** $^{13}C$ NMR ($CDCl_3$) spectrum of 2-(4-chlorophenyl)furan (**11a**):

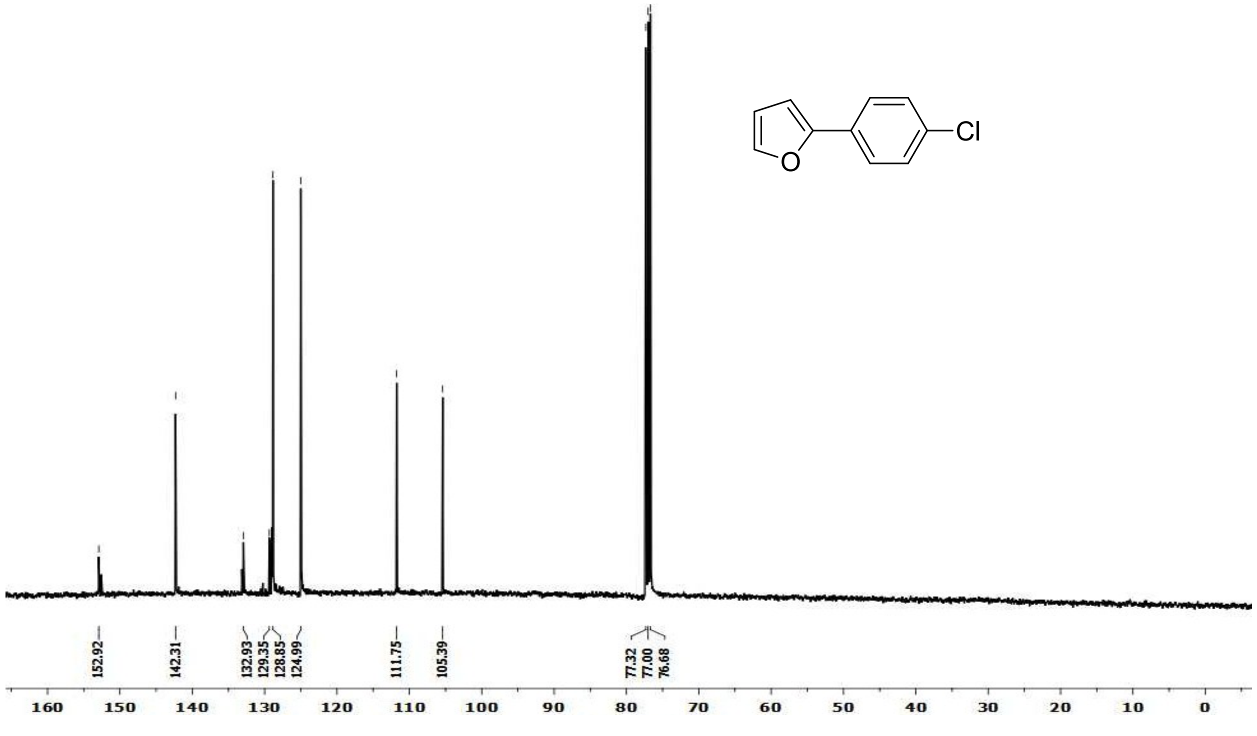

**Figure S50.** $^{1}H$ NMR ($CDCl_3$) spectrum of 2-(4-nitrophenyl)furan (**11b**):

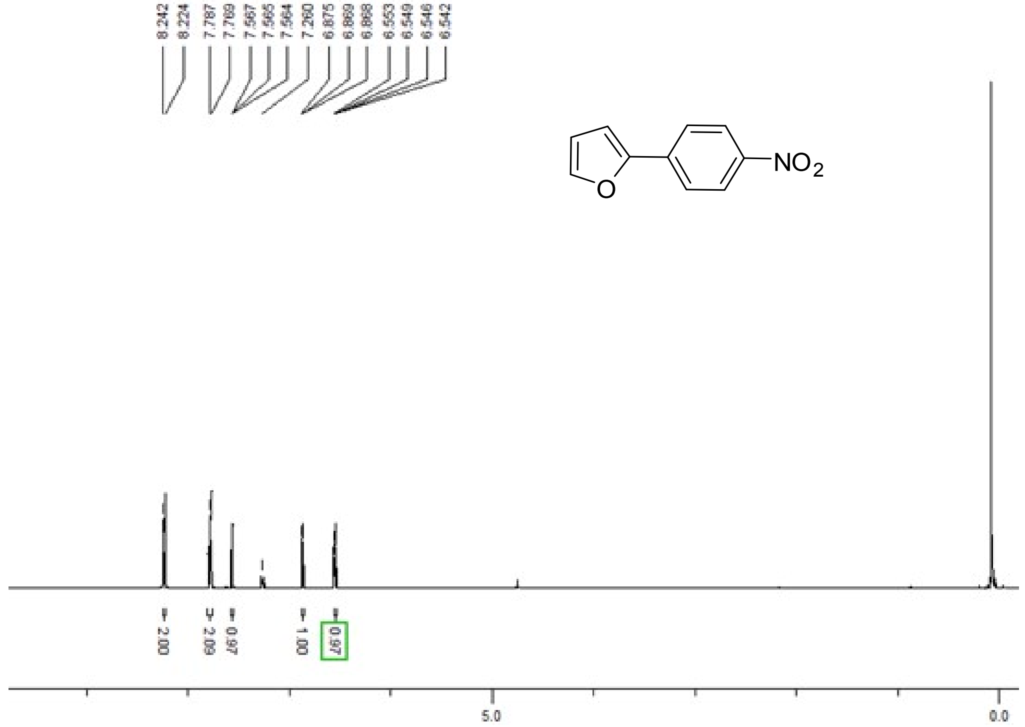


**Figure S51.** $^{13}C$ NMR ($CDCl_3$) spectrum of 2-(4-nitrophenyl)furan (**11b**):

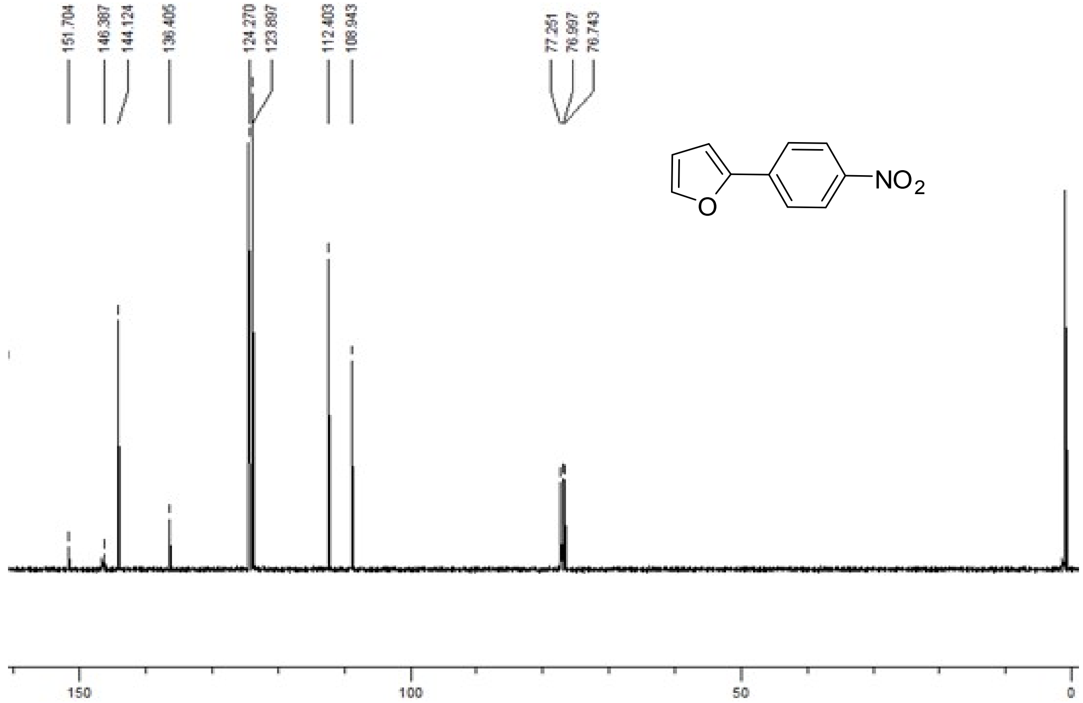

**Figure S52.** $^{1}$H NMR ($CDCl_3$) spectrum of 2-(p-tolyl)furan (**11c**):

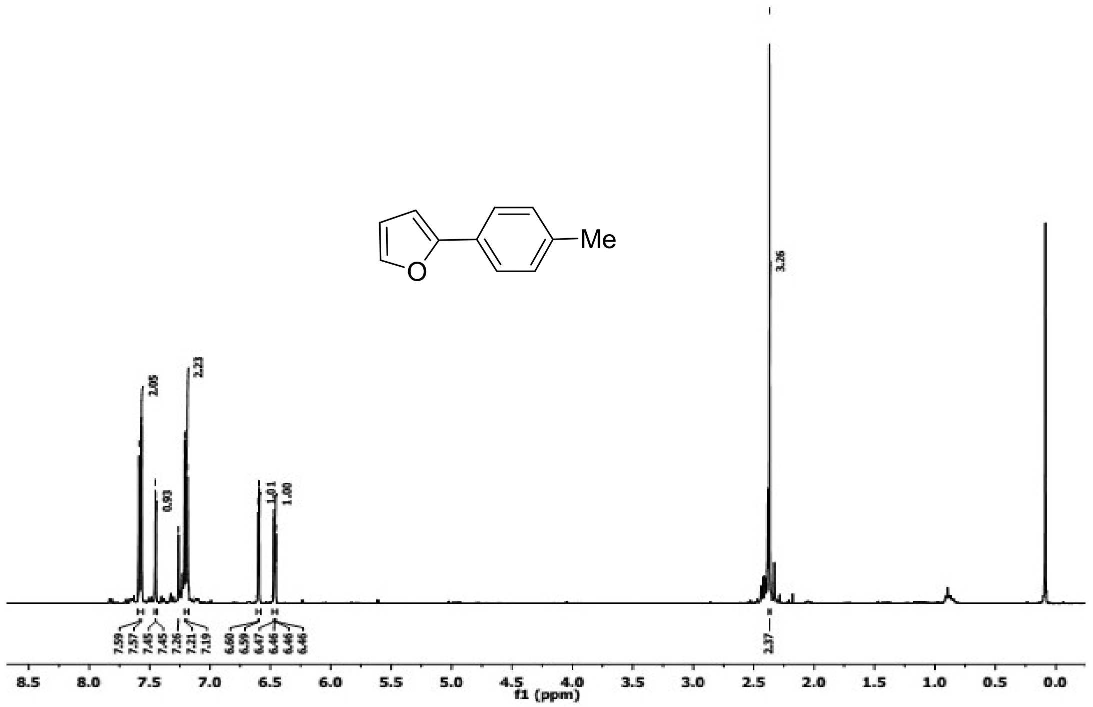

**Figure S53.** $^{13}$C NMR ($CDCl_3$) spectrum of 2-(p-tolyl)furan (**11c**):

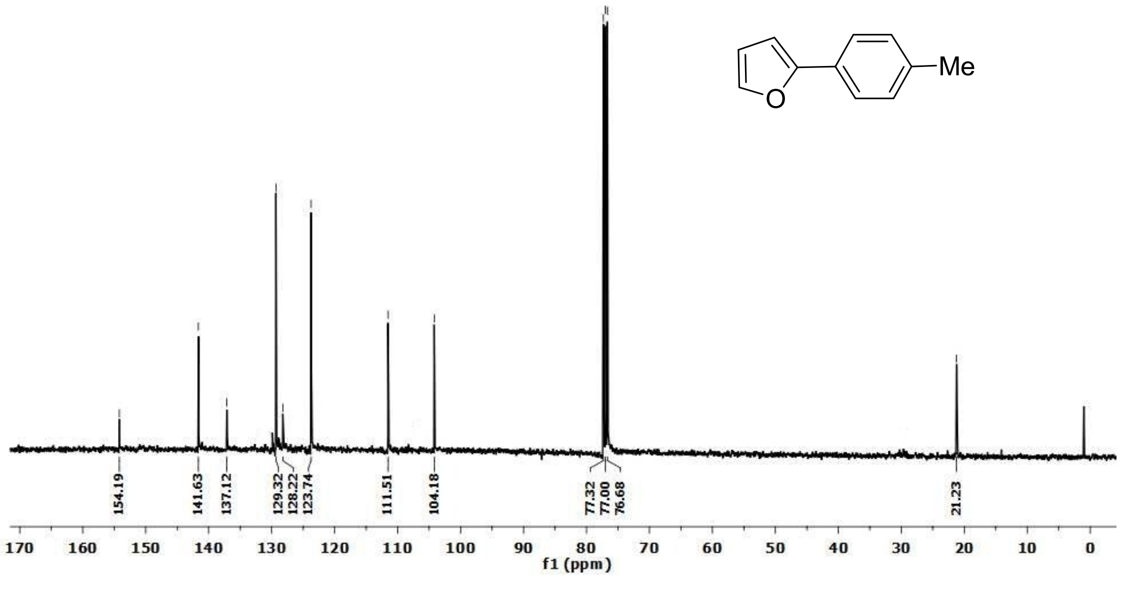

**Figure S54.** $^{1}H$ NMR ($CDCl_3$) spectrum of 2-phenylfuran (**11d**):

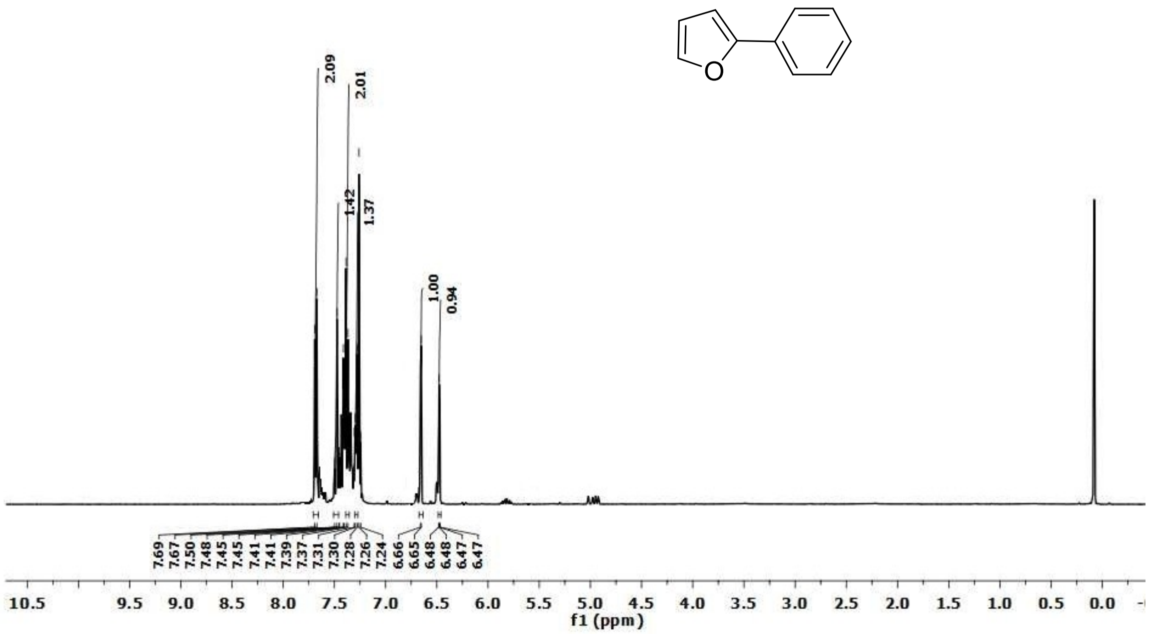

**Figure S55.** $^{13}C$ NMR ($CDCl_3$) spectrum of 2-phenylfuran (**11d**):

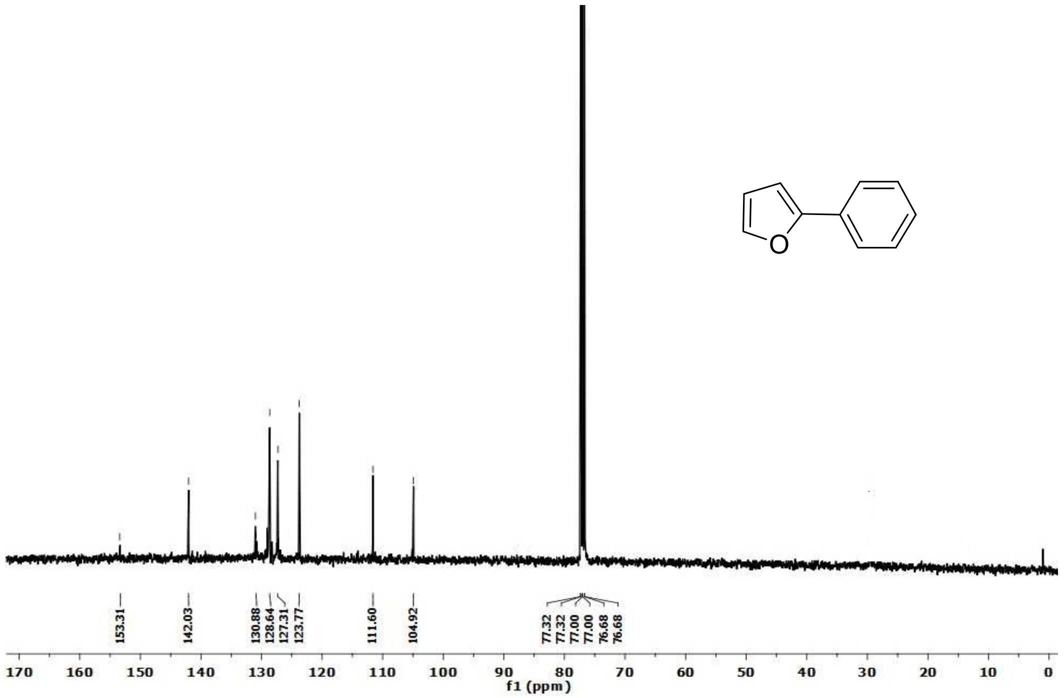

**Figure S56.** $^{1}H$ NMR ($CDCl_3$) spectrum of 2-(4-methoxyphenyl)furan (**11e**):

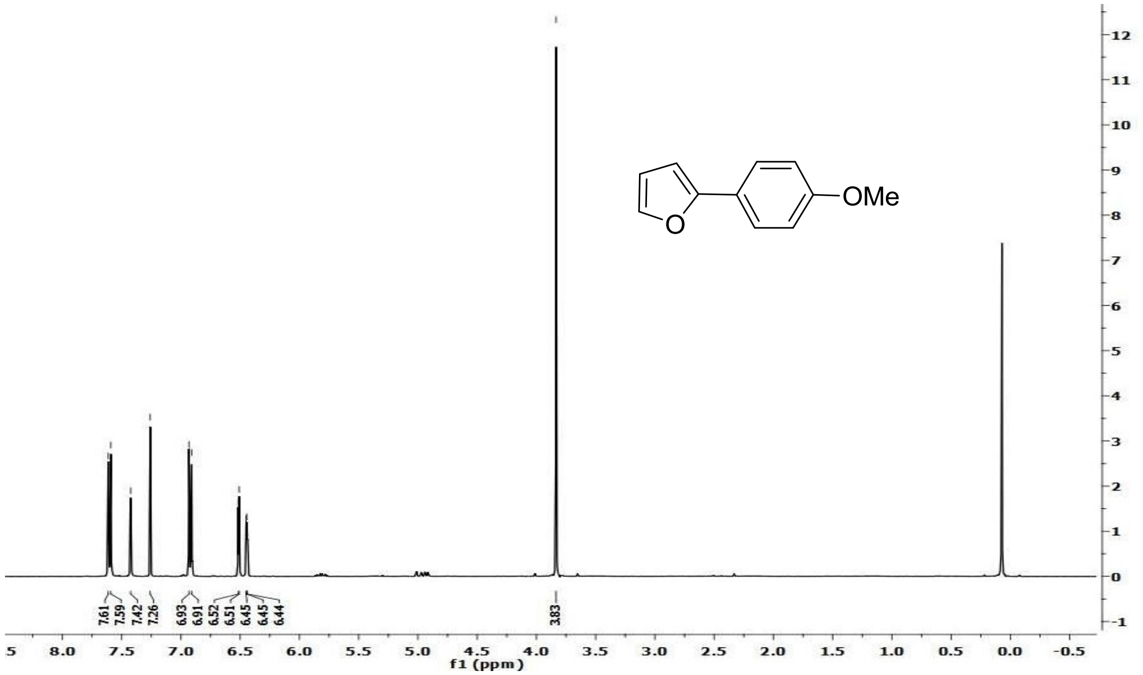


**Figure S57.** $^{13}C$ NMR ($CDCl_3$) spectrum of 2-(4-methoxyphenyl)furan (**11e**):

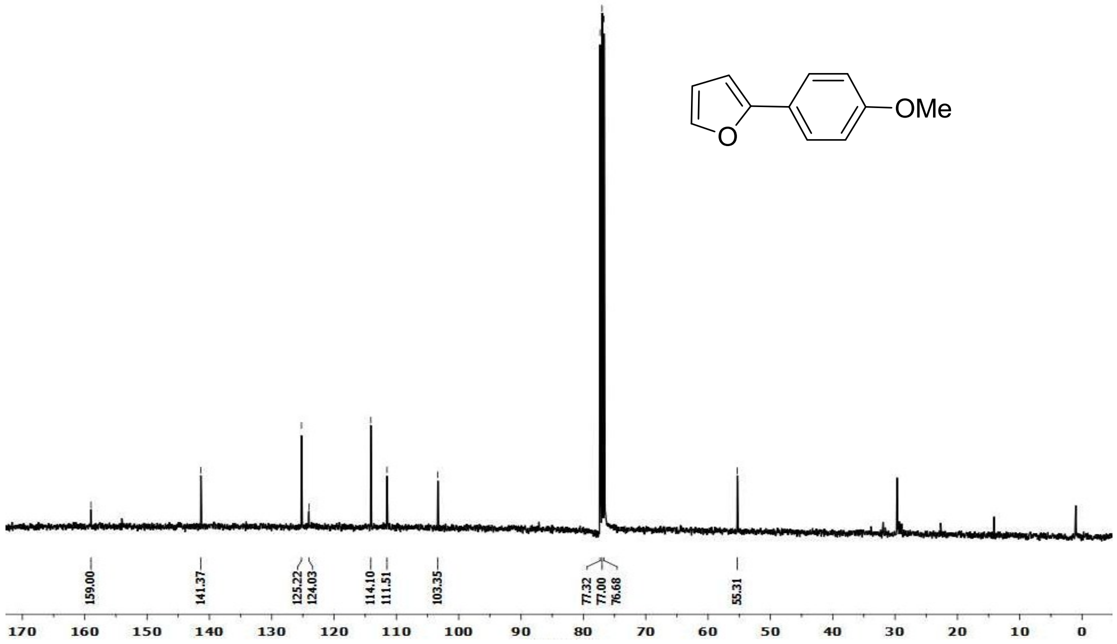

**Figure S58.** $^{1}$H NMR ($CDCl_3$) spectrum of 2-(4-cyanophenyl)furan (**11g**):

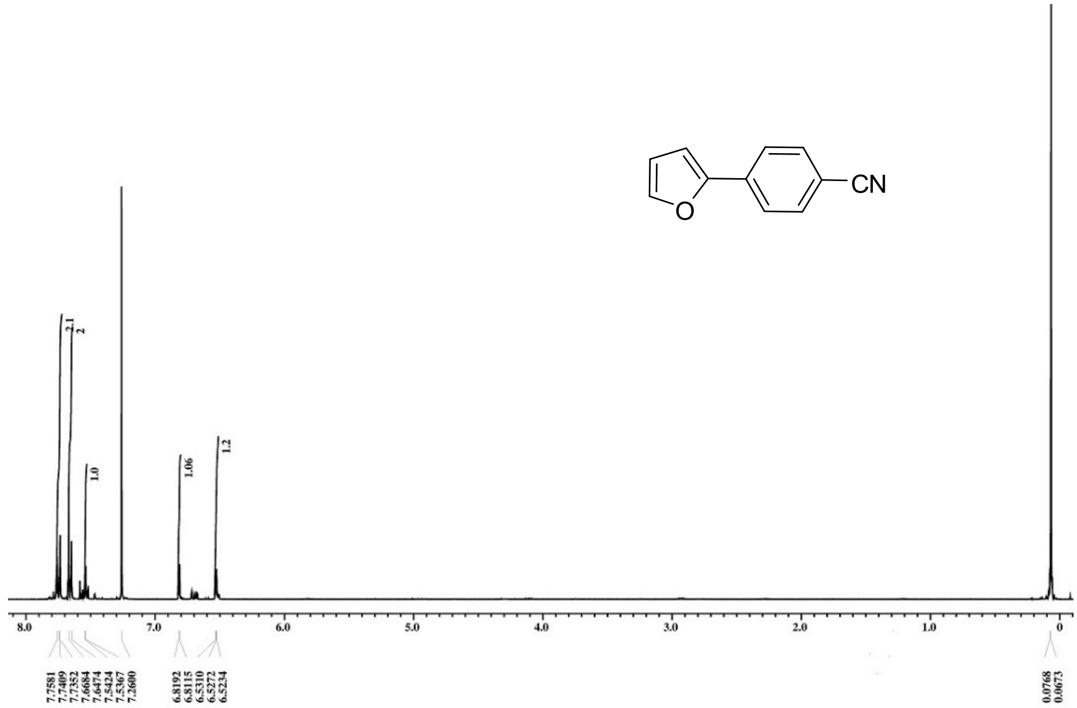


**Figure S59.** $^{13}$C NMR ($CDCl_3$) spectrum of 2-(4-cyanophenyl)furan (**11g**):

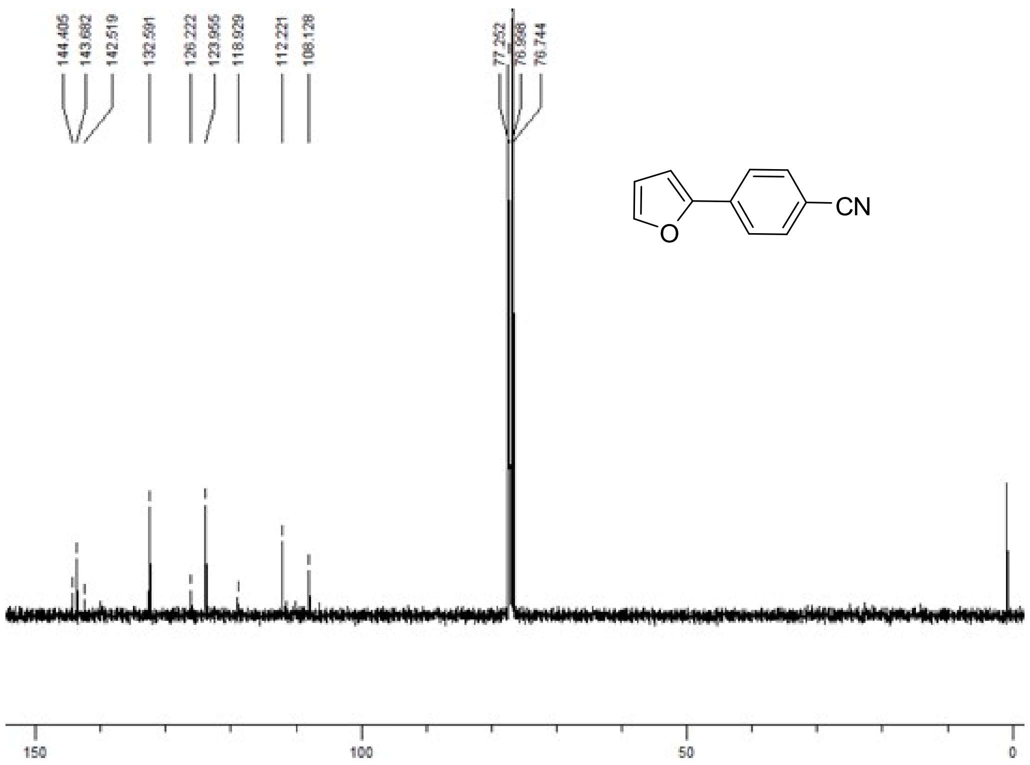

**Figure S60.** $^{1}$H NMR ($CDCl_3$) of 2-(2-nitrophenyl)furan (**11h**):

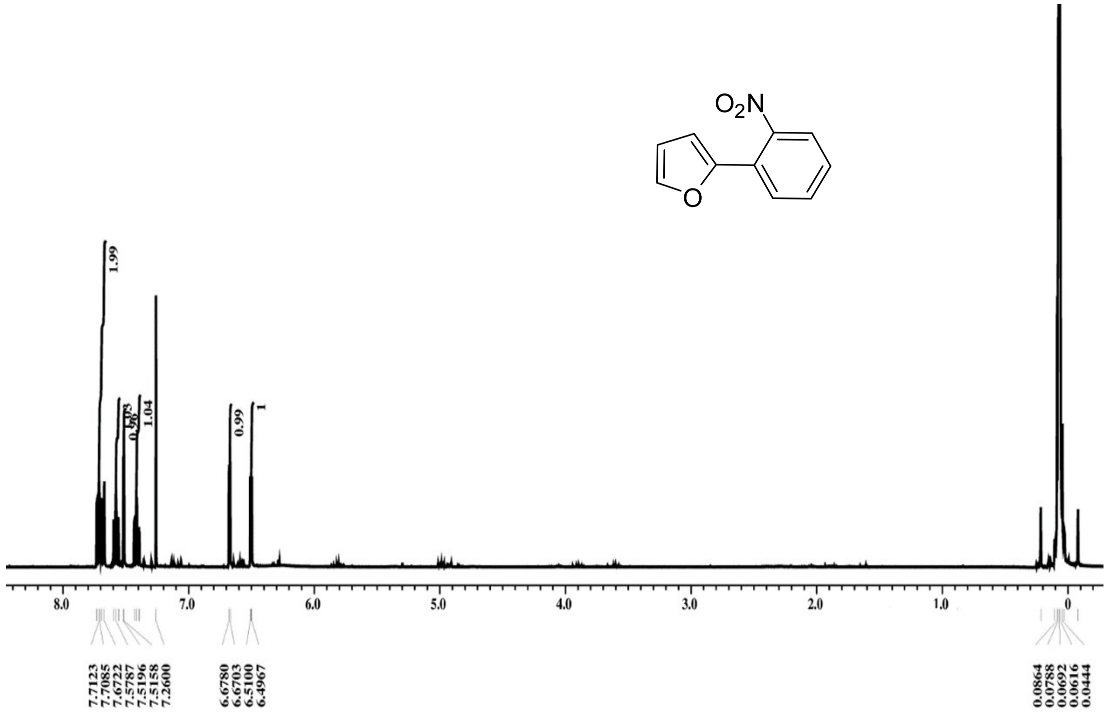


**Figure S61.** $^{13}$C NMR ($CDCl_3$) spectrum of 2-(2-nitrophenyl)furan (**11h**):

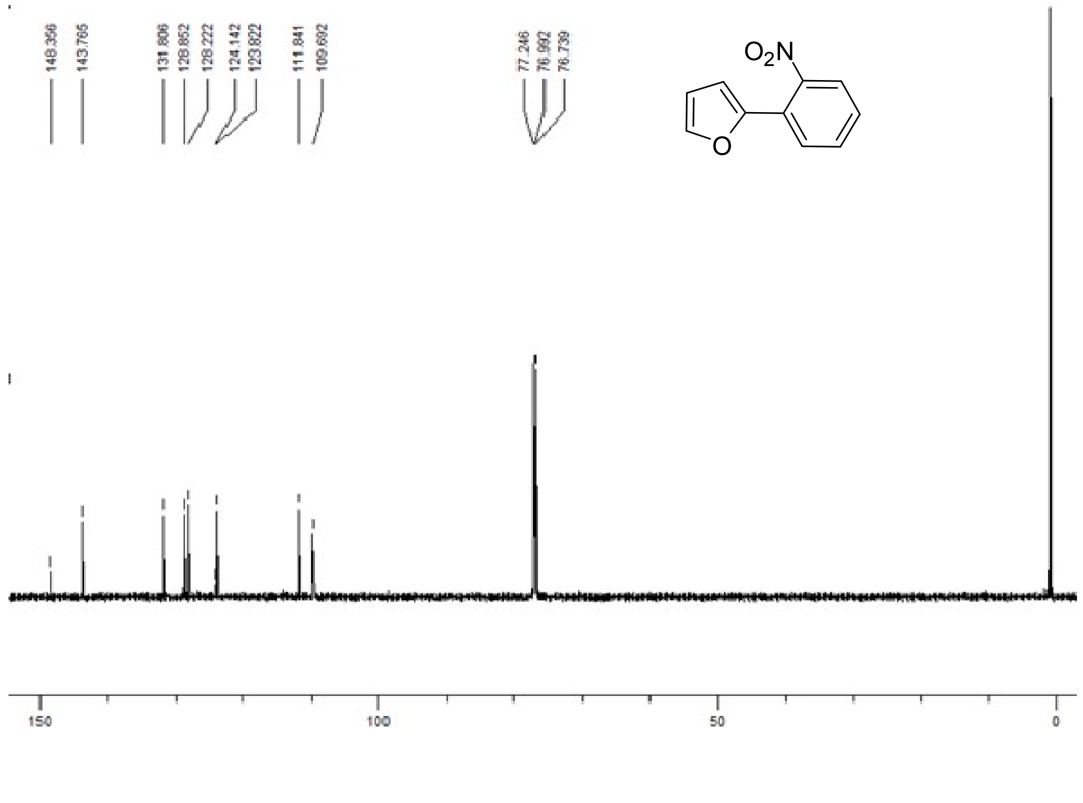

**Figure S62.** $^{1}H$ NMR ($CDCl_3$) spectrum of 2-(4-chlorophenyl)pyridine (**15a**):

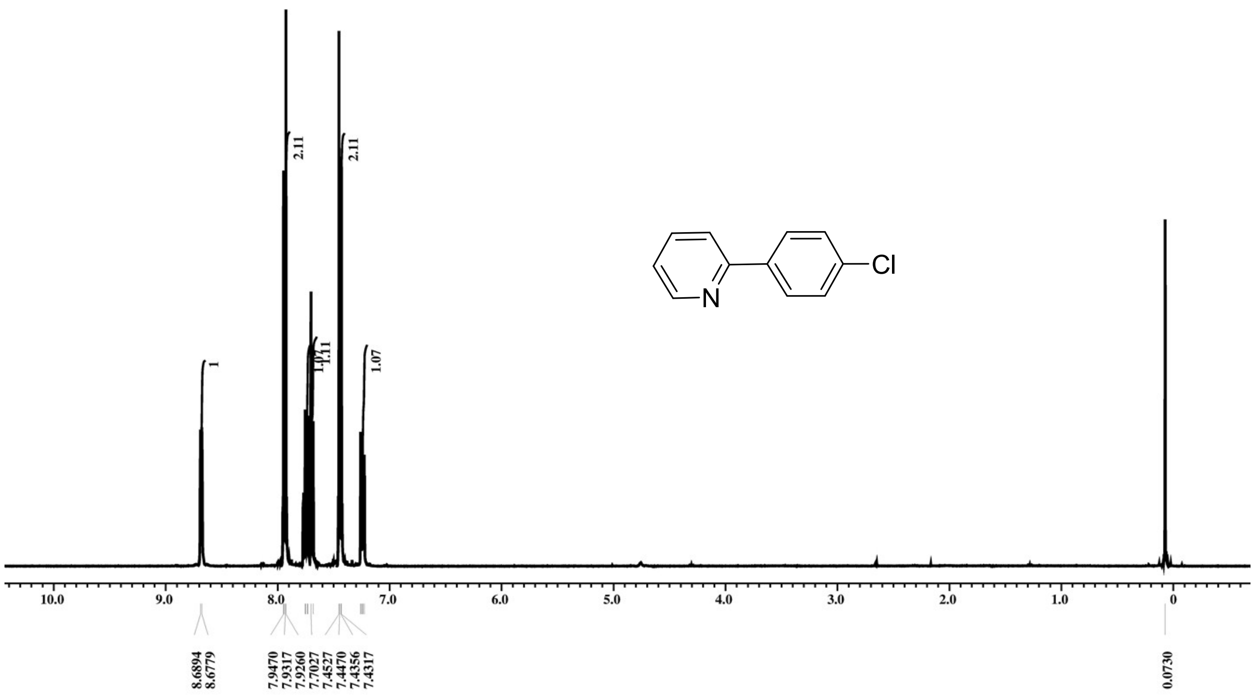


**Figure S63.** $^{13}C$ NMR ($CDCl_3$) spectrum of 2-(4-chlorophenyl)pyridine (**15a**):

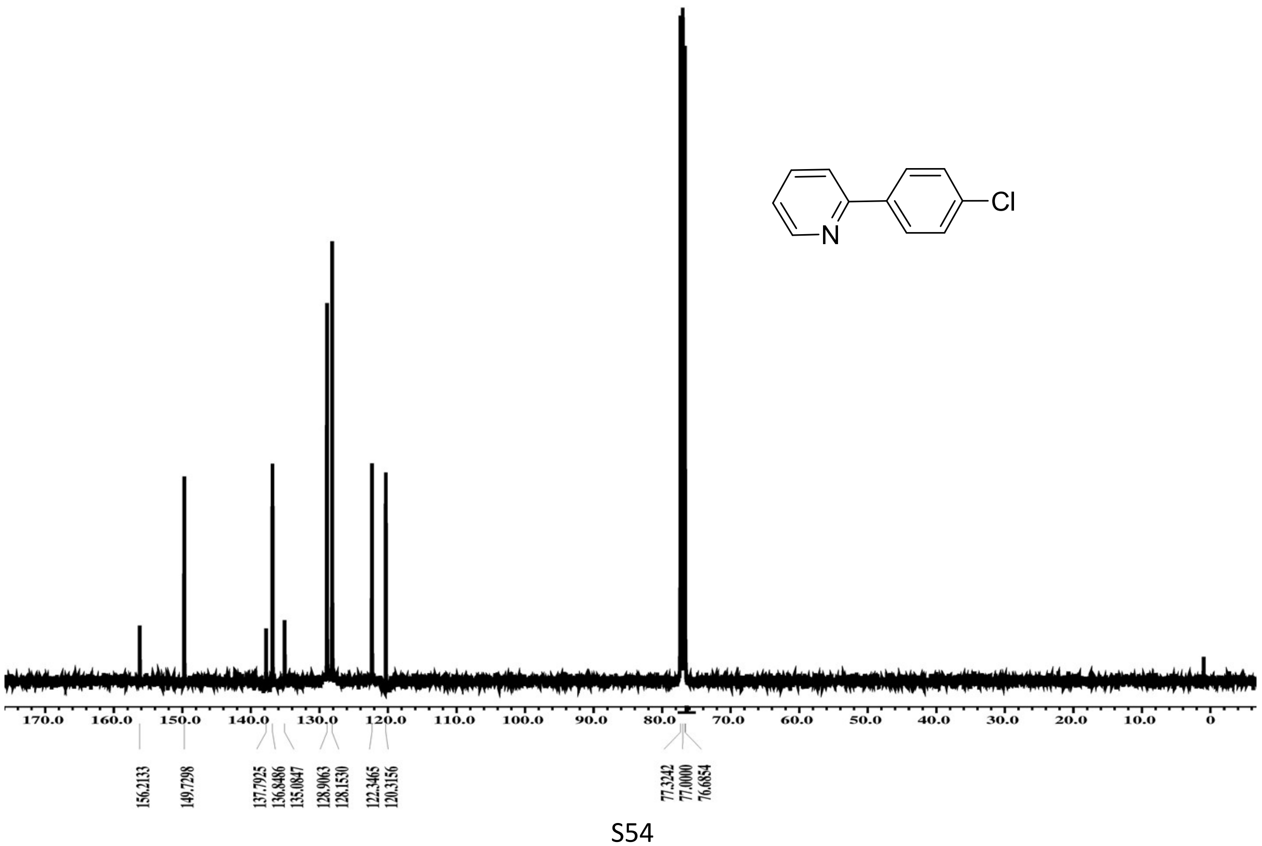

**Figure S64.** $^{1}$H NMR ($CDCl_3$) spectrum of 2-(4-nitrophenyl)pyridine (**15b**):

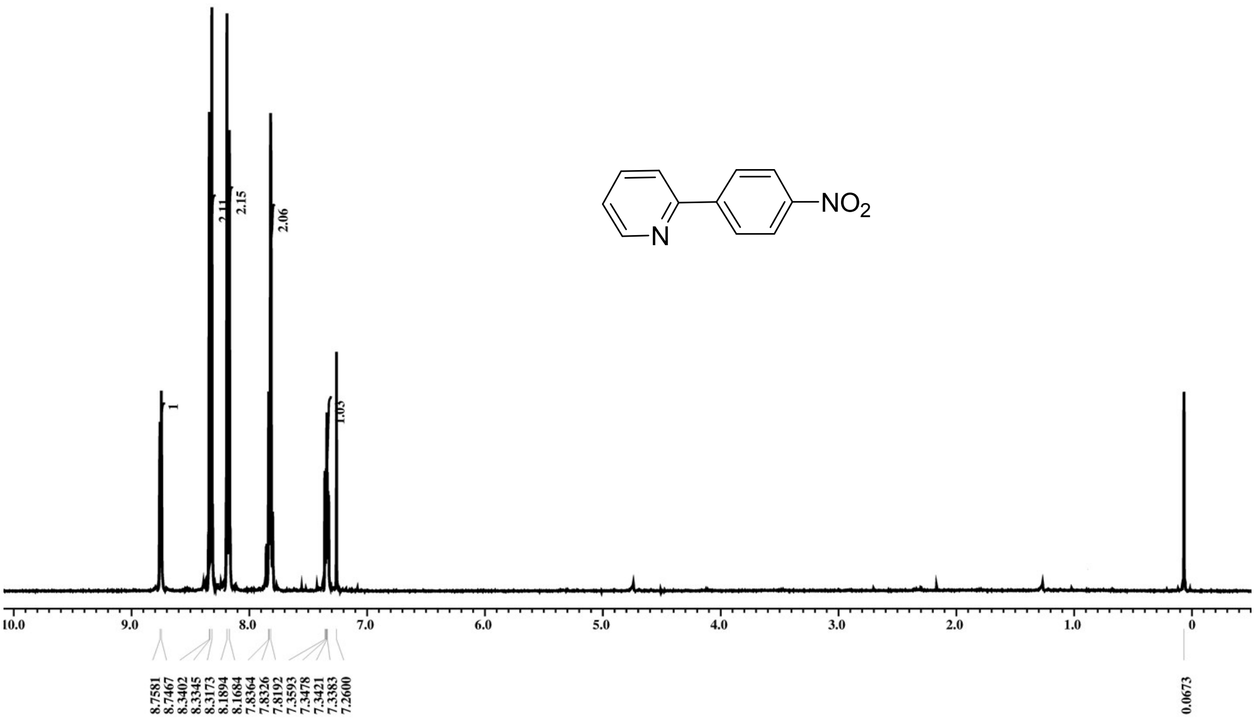


**Figure S65.** $^{13}$C NMR ($CDCl_3$) spectrum of 2-(4-nitrophenyl)pyridine (**15b**):

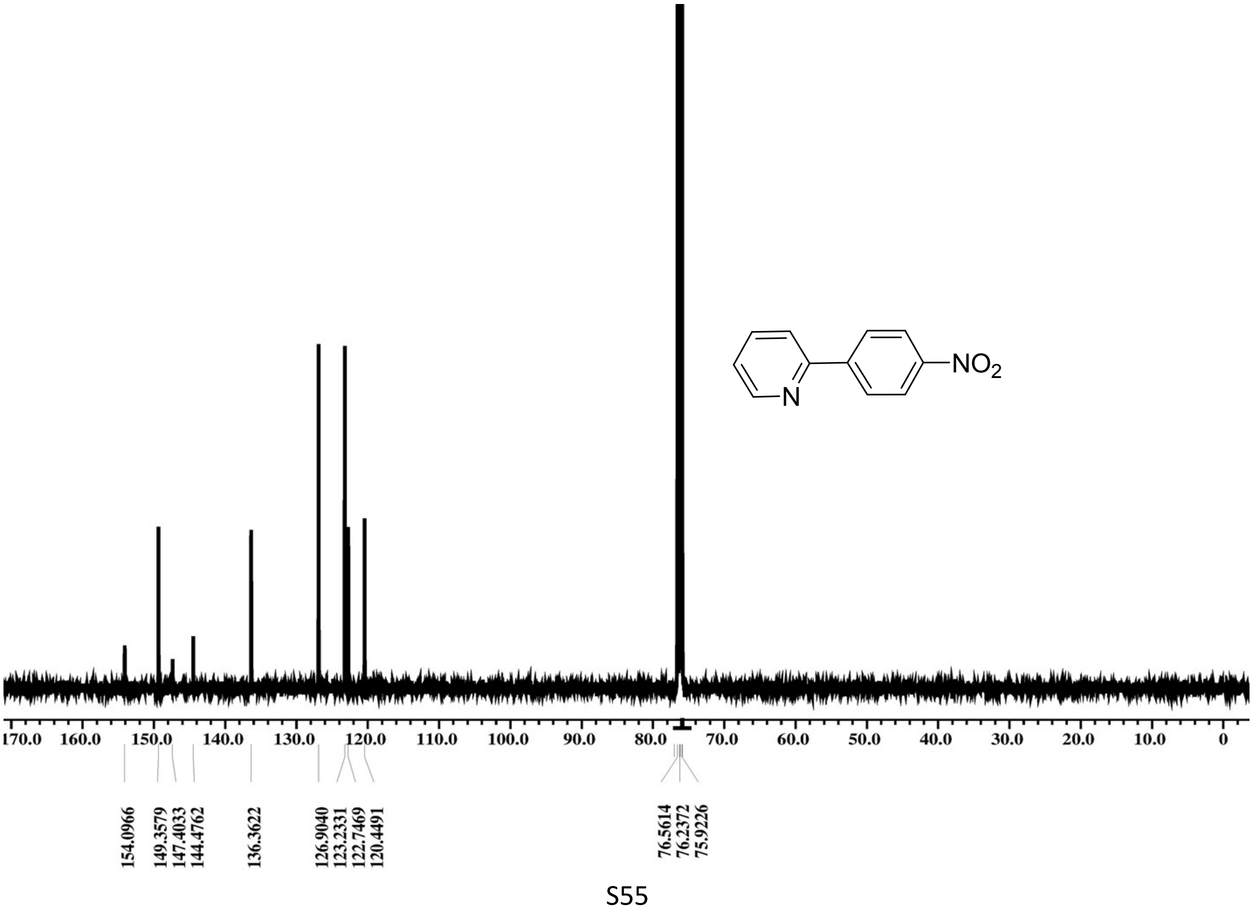

**Figure S66.** $^{1}$H NMR ($CDCl_3$) spectrum of 2-(*p*-tolyl)pyridine (**15c**):

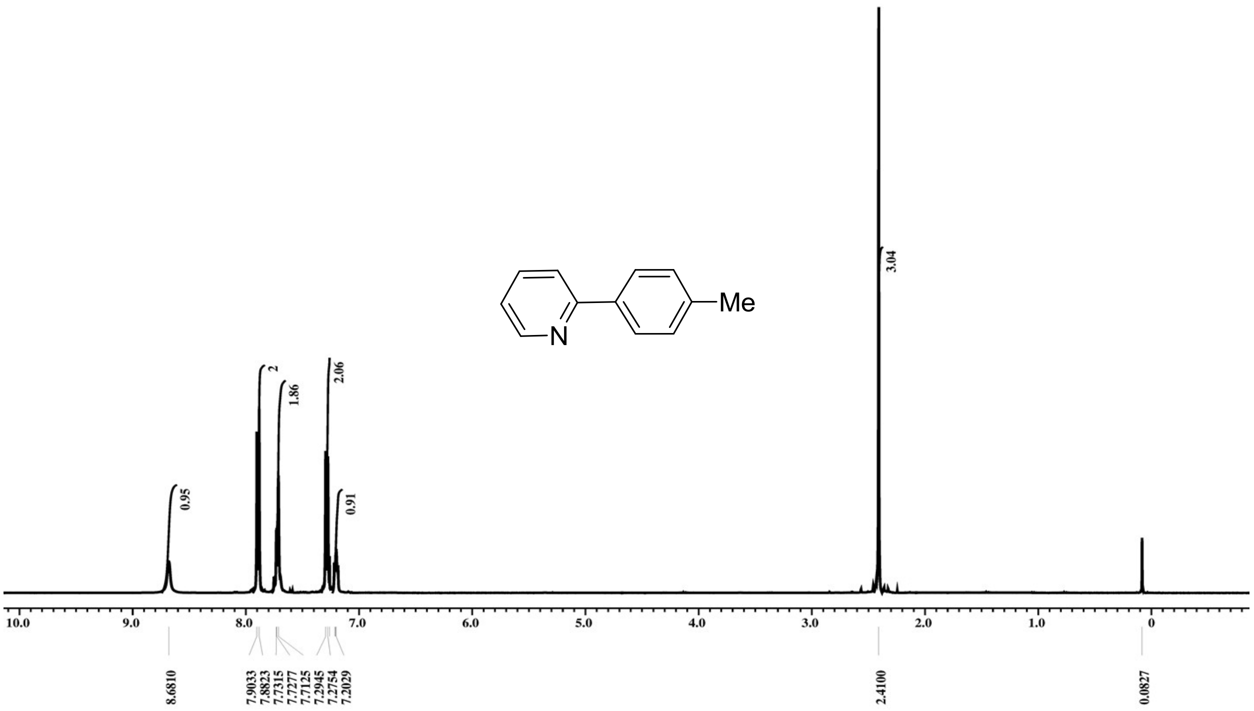


**Figure S67.** $^{13}$C NMR ($CDCl_3$) spectrum of 2-(*p*-tolyl)pyridine (**15c**):

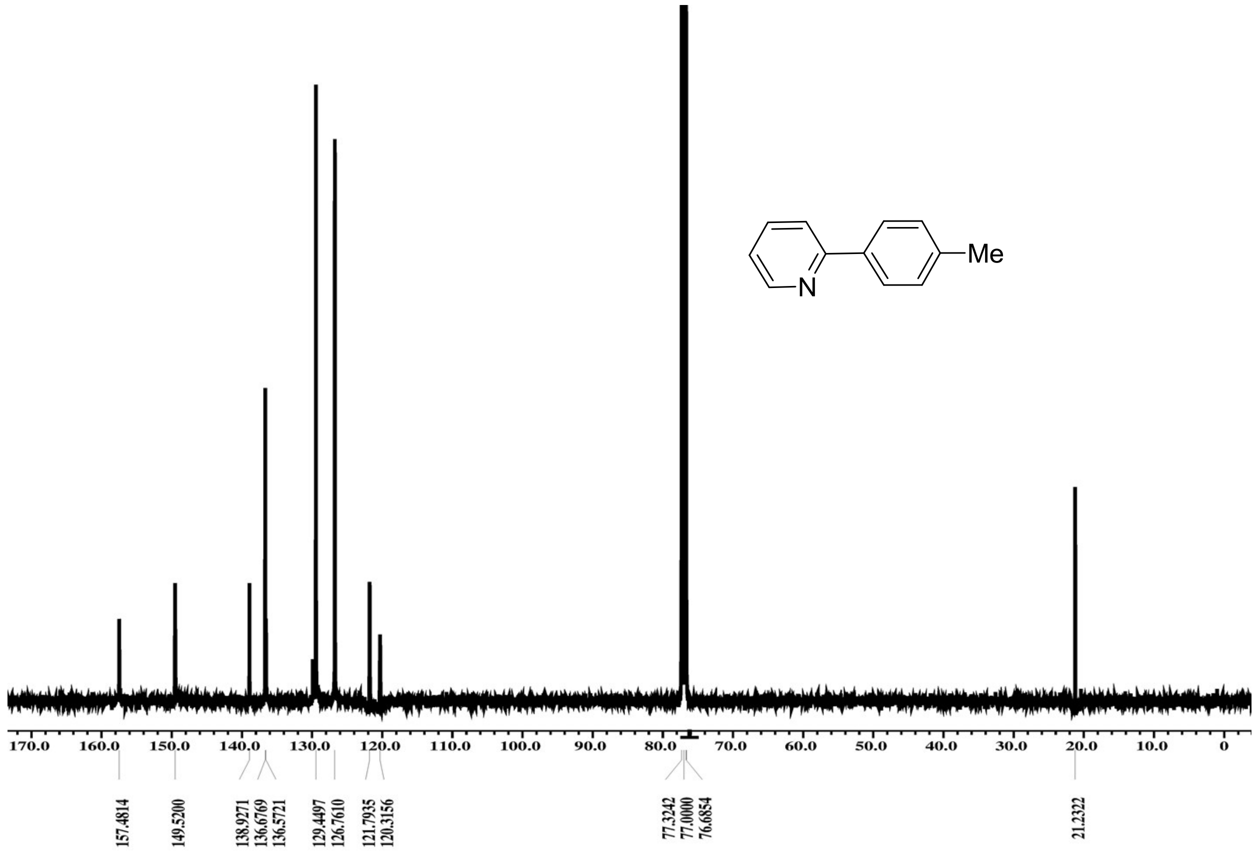

**Figure S68.** $^{1}$H NMR ($CDCl_3$) spectrum of 2-phenylpyridine (**15d**):

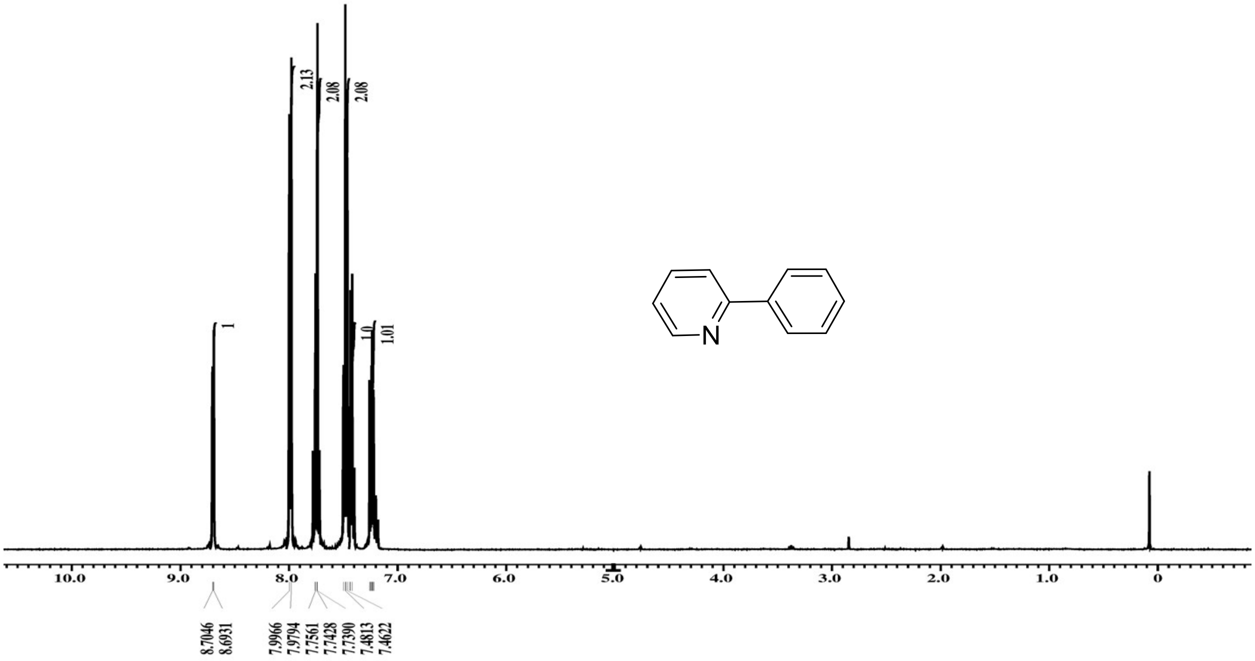


**Figure S69.** $^{13}$C NMR ($CDCl_3$) spectrum of 2-phenylpyridine (**15d**):

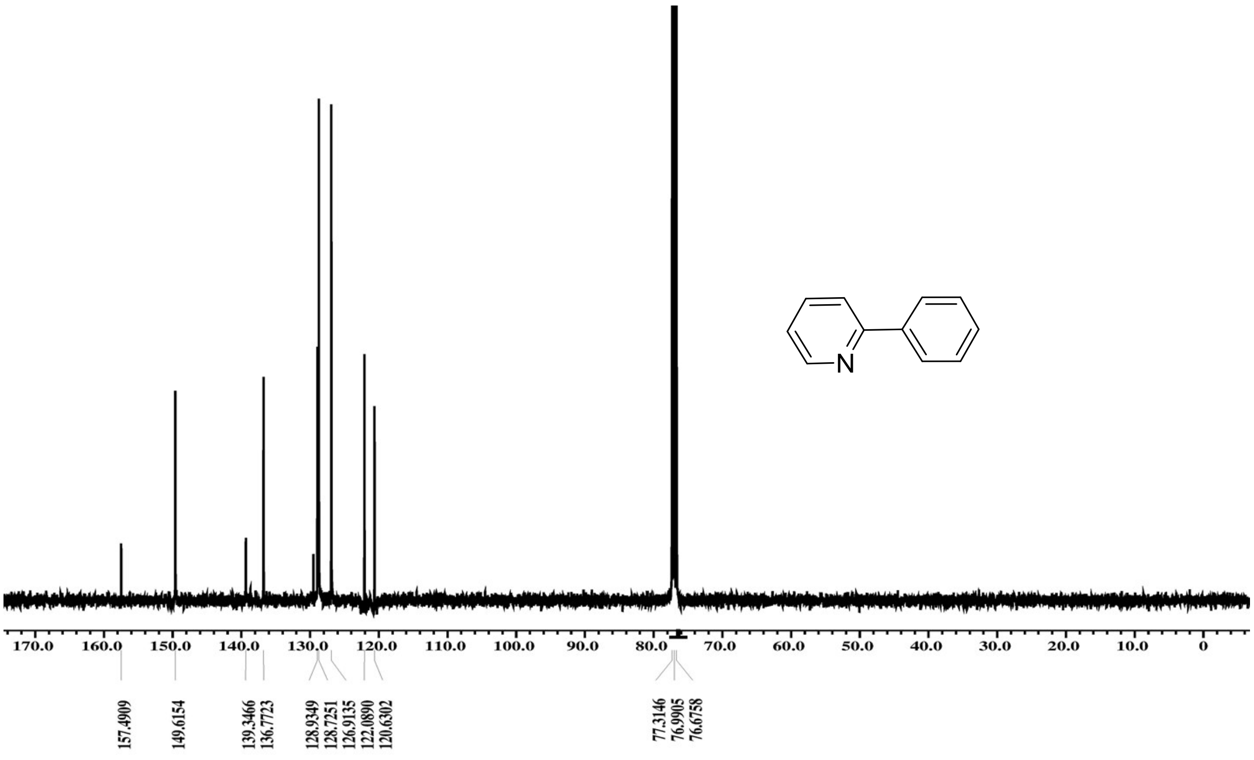

**Figure S70.** $^1$H NMR ($CDCl_3$) spectrum of 2-(4-methoxyphenyl)pyridine (**15e**):

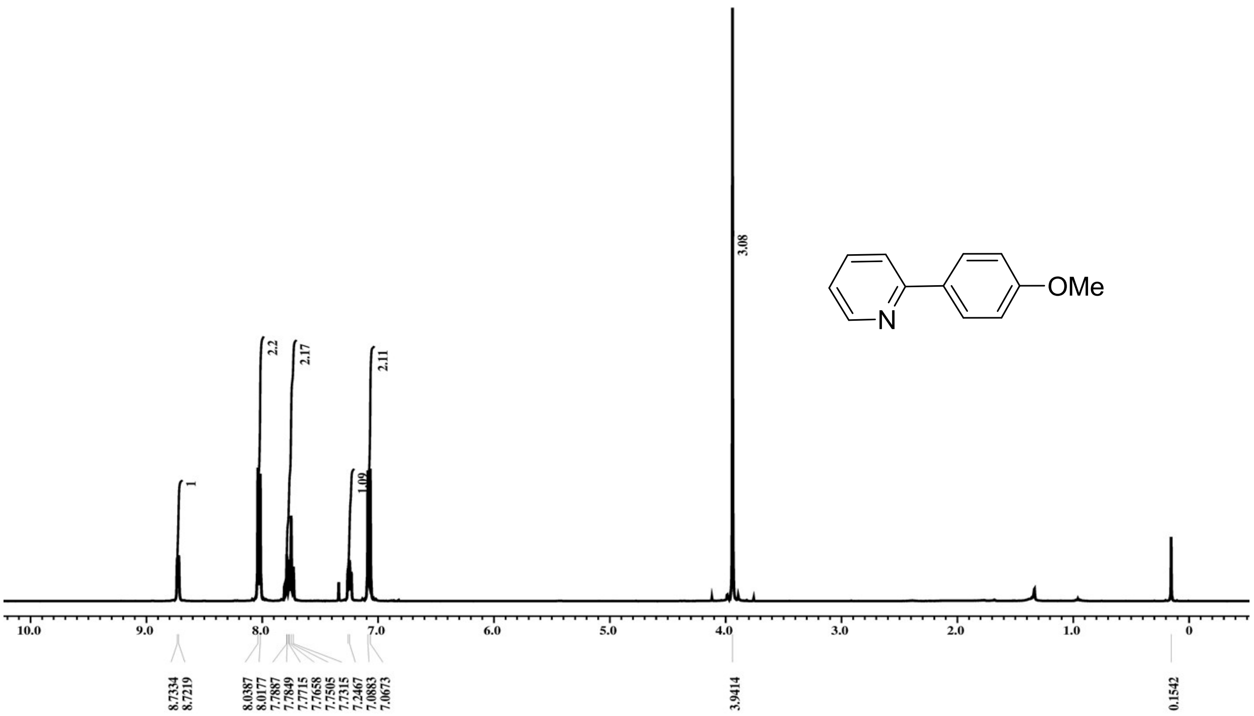


**Figure S71.** $^{13}$C NMR ($CDCl_3$) spectrum of 2-(4-methoxyphenyl)pyridine (**15e**):

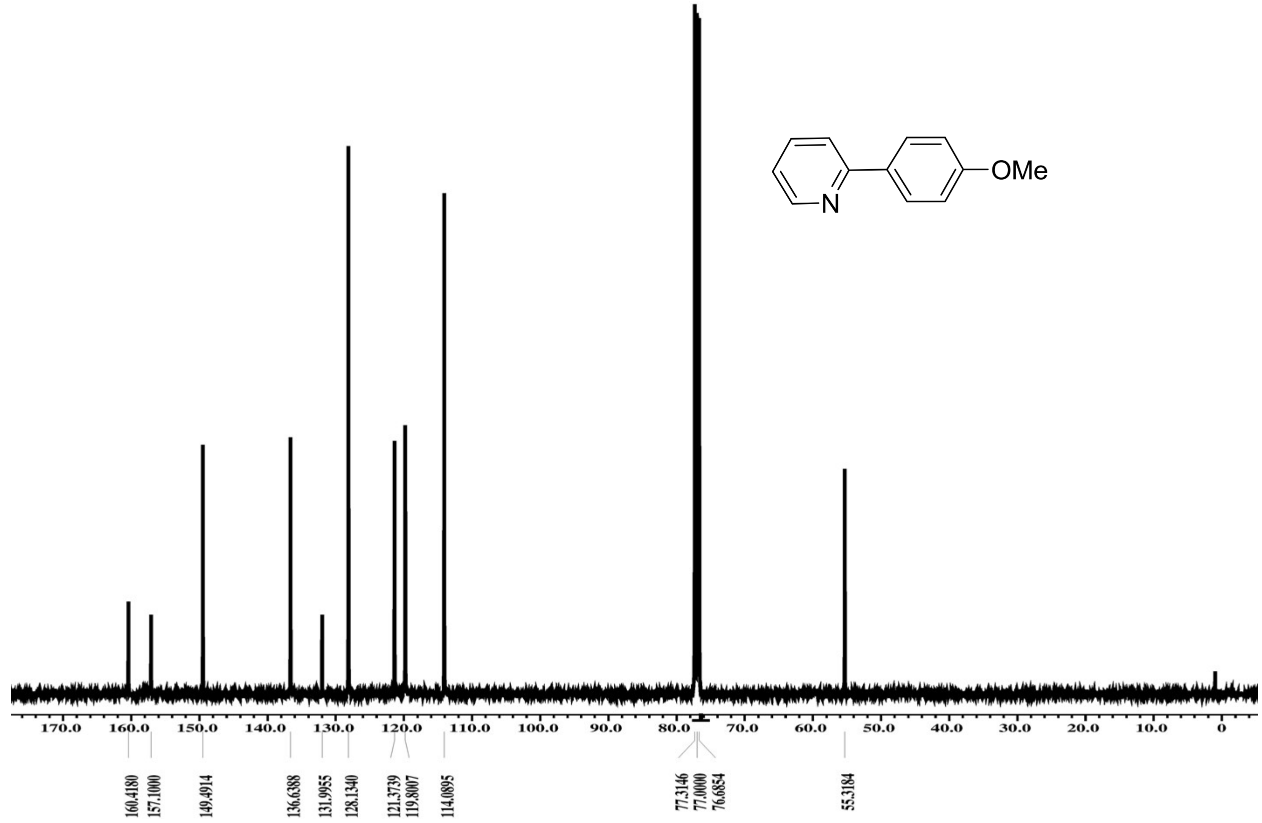

**Figure S72.** $^{1}$H NMR ($CDCl_3$) spectrum of 2-(4-fluorophenyl)pyridine (**15f**):

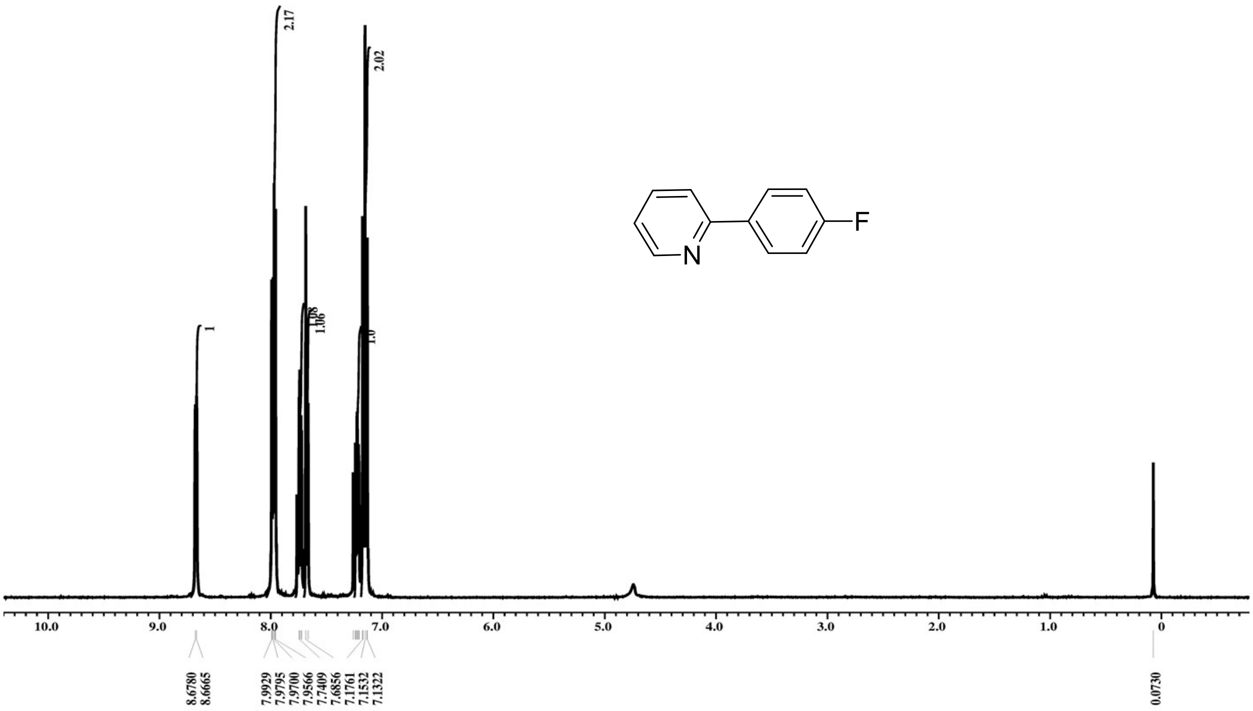


**Figure S73.** $^{13}$C NMR ($CDCl_3$) spectrum of 2-(4-fluorophenyl)pyridine (**15f**):

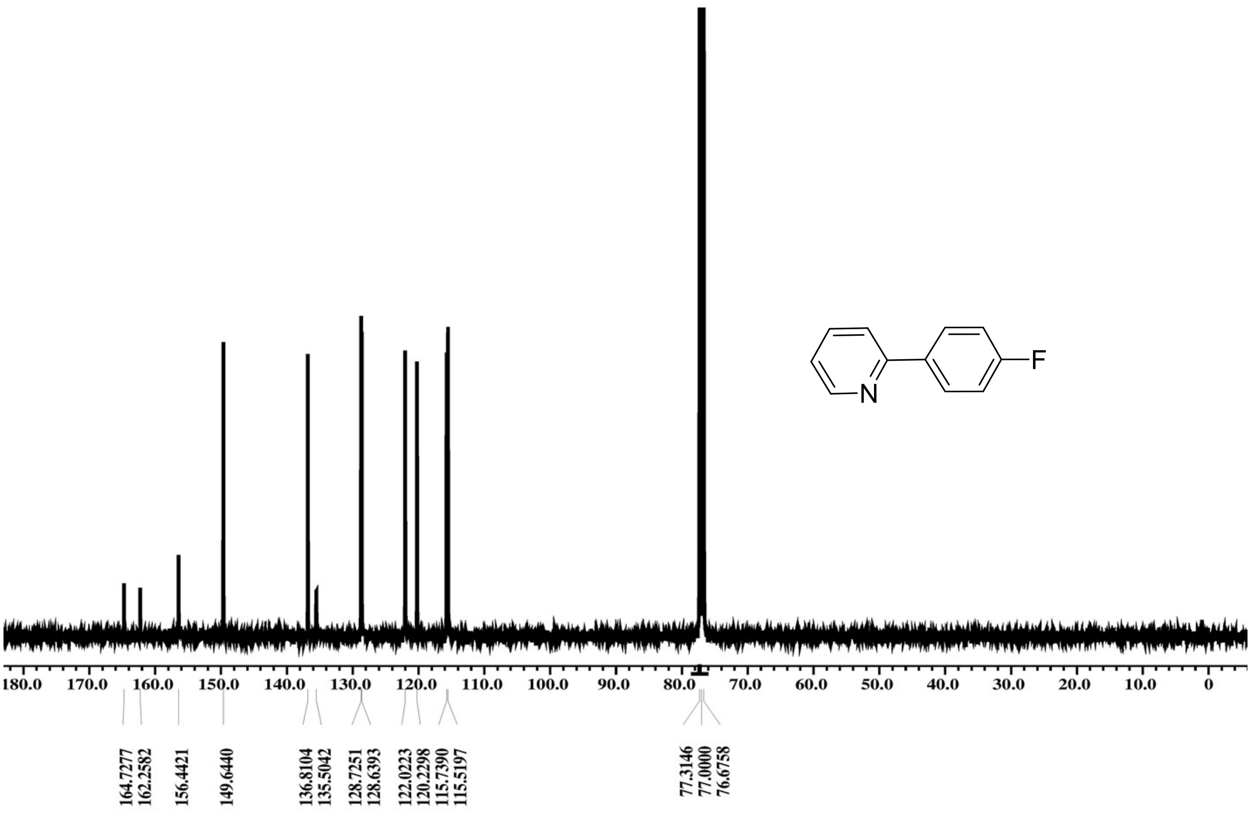

**Figure S74.** $^{1}H$ NMR ($CDCl_3$) spectrum of 2-(3,5-dimethylphenyl)pyridine (**15I**):

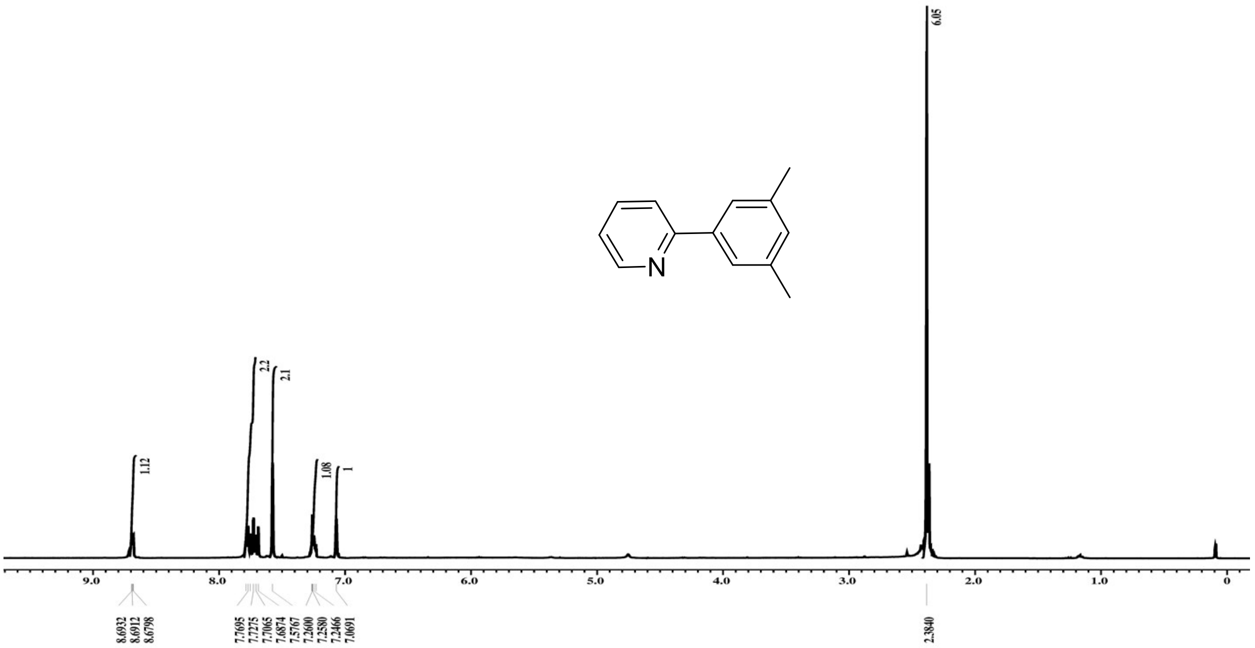


**Figure S75.** $^{13}C$ NMR ($CDCl_3$) spectrum of 2-(3,5-dimethylphenyl)pyridine (**15I**):

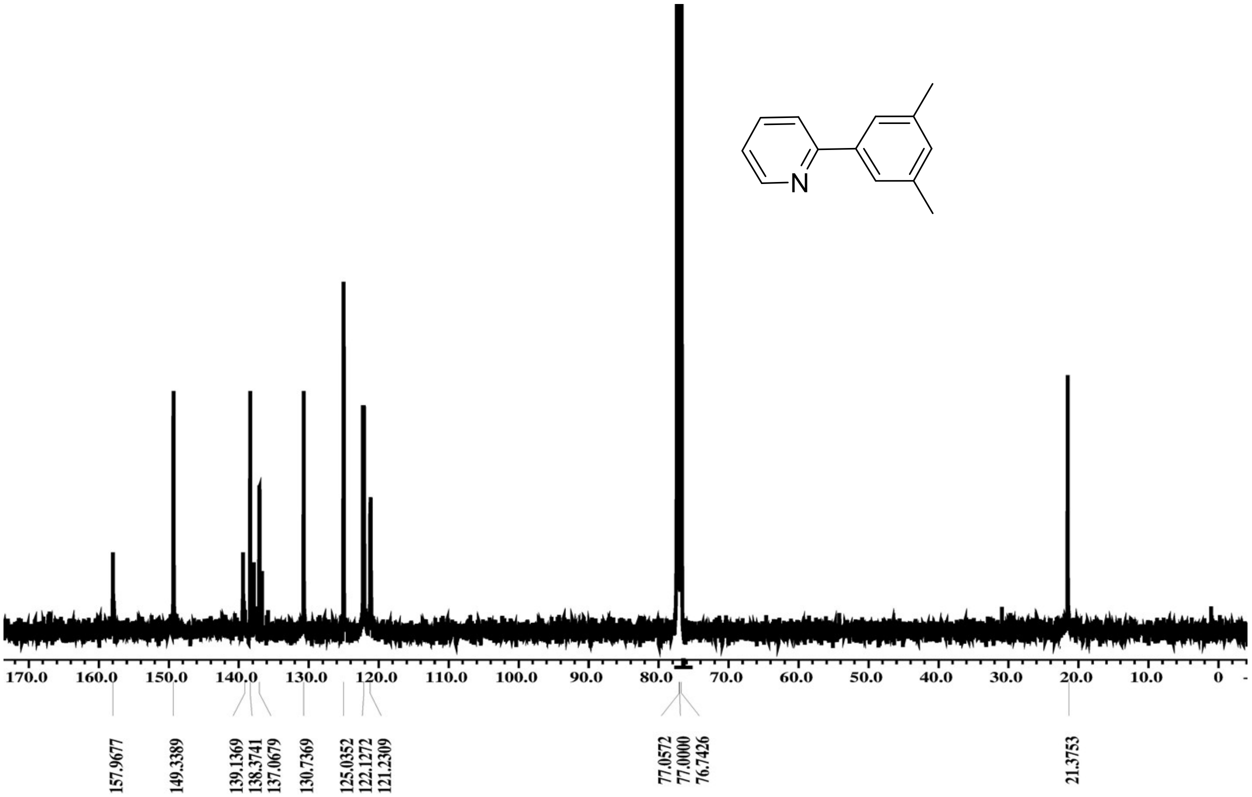

**Figure S76.** $^{1}$H NMR ($CDCl_3$) spectrum of TEMPO-trapped intermediate **16**:

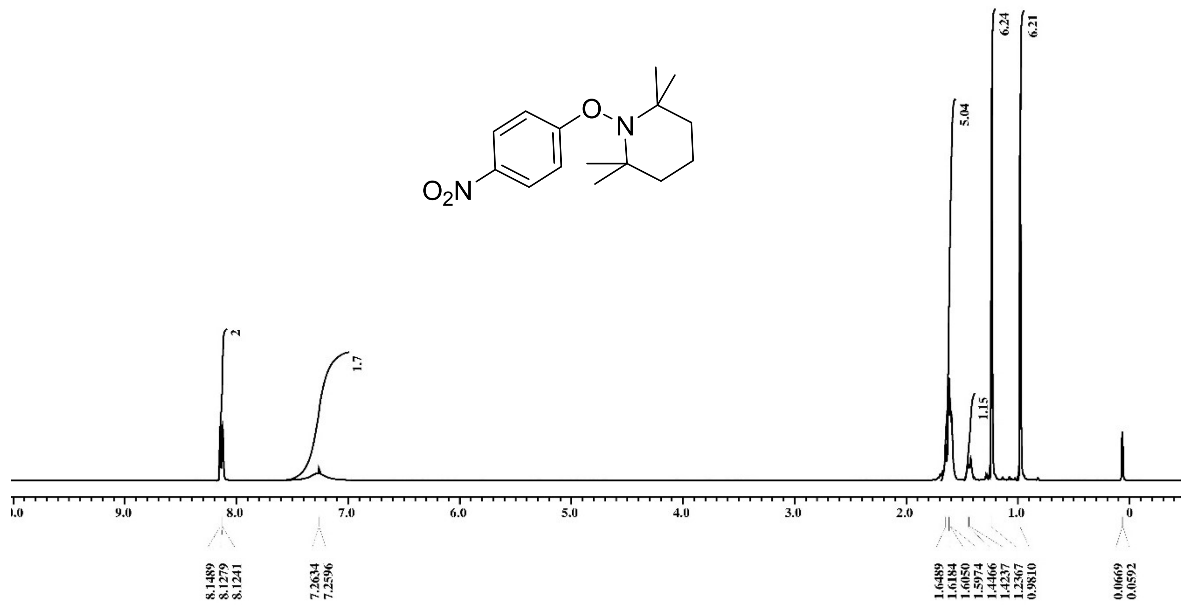


**Figure S77.** $^{13}$C NMR ($CDCl_3$) spectrum of TEMPO-trapped intermediate **16**:

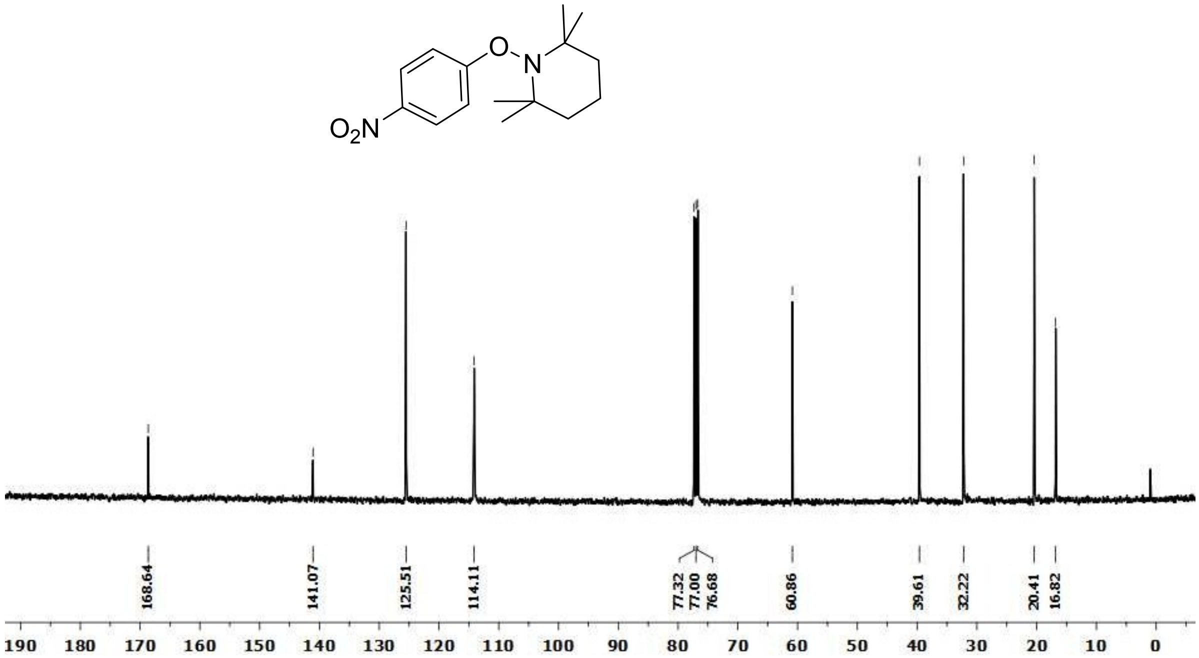

**Figure S78.** $^{1}$H NMR (DMSO-$d_6$) spectrum of Complex **I**:

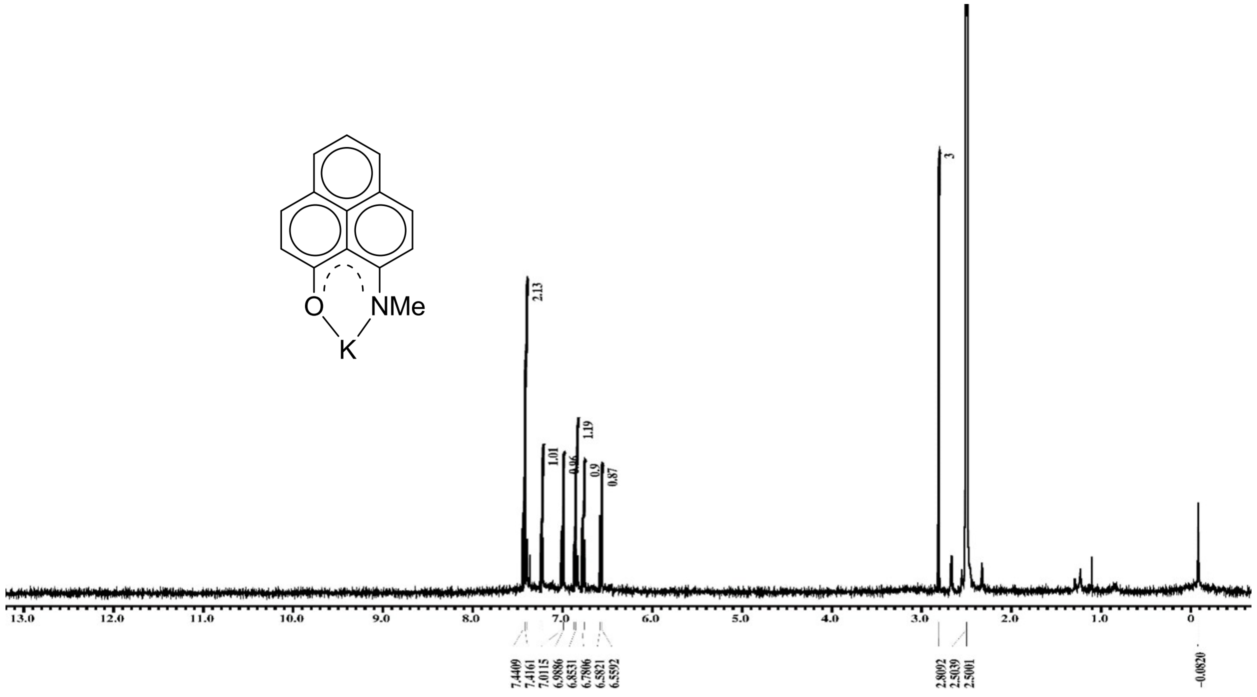

## IV. X-ray structure and data of compound 17:

Suitable single crystal of **17** was selected and an intensity data were collected on a SuperNova, Dual, Cu at zero, Eos diffractometer. Using Olex2[17] the structure was solved with the Superflip[18] structure solution program using Charge Flipping and refined with the ShelXL[19] refinement package using Least Squares minimization.

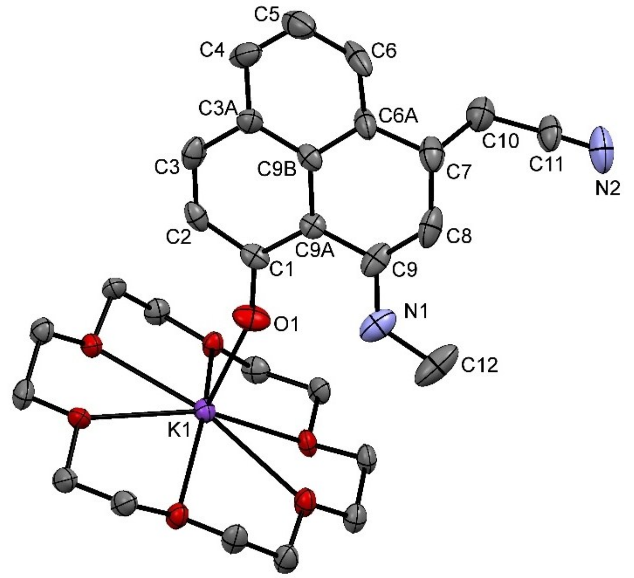


**Figure S79**. ORTEP diagram of **17** with thermal ellipsoids were drawn at 50% probability. The hydrogen atoms are omitted for clarity. Selected bond lengths [Å] and angles [°] in: O1-K1 2.570(4), O1-C1 1.300(6), C10-C11 1.397(8), C7-C10 1.602(8), N2-C11 1.128(7); K1-O1-C1 139.7(3), C6A-C7-C8 112.8(4), C9-C8-C7 123.3(5), C11-C10-C7 111.5(5), N2-C11-C10 173.9(7).

.

**Table S2.** Crystallographic and Data Collection parameters for Compound **17**

| | Compound **17** |
|---|---|
| Empirical formula | $C_{28}H_{37}KN_2O_7$, $C_2H_3N$ |
| Formula weight | 593.75 |
| Temperature/K | 100 |
| Crystal system | Triclinic |
| Space group | P-1 |
| *a* (Å) | 12.0598 (7) |
| *b* (Å) | 16.2046(10) |
| *c* (Å) | 17.0517(10) |
| $\alpha$ (°) | 71.592(5) |
| $\beta$ (°) | 86.228(5) |
| $\gamma$ (°) | 78.678(5) |
| V(Å$^3$) | 3100.3(3) |
| Z | 4 |
| $D_{calc}$(g/cm$^3$) | 1.272 |
| *F* (000) | 1264 |
| $\mu$ (mm$^{-1}$) | 0.220 |
| $\theta$ Range (°) | 1.7 to 25.0 |
| Goodness-of-fit | 1.03 |

| | |
|---|---|
| R, wR2 | 0.0847, 0.2190 |
| Ri ndexes [al l data] | 0.0847 and 0.2190 |
| Largest difference in peak and hole (e/ Å$^3$) | -0.46/0.88 |
| CCDC Number | 1520738 |

**Table S3.** Selected Bond Distances (Å) and Angles (°) for Compound **17**

| | | | | | |
|---|---|---|---|---|---|
| K1 | -O1 | 2.570(4) | N1 | -C16 | 1.454(8) |
| K1 | -O2 | 2.813(3) | C1 | -C2 | 1.400(9) |
| C2 | -C3 | 1.307(11) | C3 | -C4 | 1.407(10) |
| C4 | -C5 | 1.394(9) | C5 | -C6 | 1.336(10) |
| C6 | -C7 | 1.386(9) | C7 | -C8 | 1.430(9) |
| O1 | -C1 | 1.300(6) | C8 | -C9 | 1.480(9) |
| C10 | -C11 | 1.397(8) | C7 | -C10 | 1.602(8) |
| N2 | -C11 | 1.128(7) | C6A | -C7 | 1.477(7) |

| | | | | | | | |
|---|---|---|---|---|---|---|---|
| K1 | -O1 | -C1 | 139.7(3) | O1 | -K1 | -O2 | 91.97(12) |
| C9B | -C6A | -C7 | 121.9(5) | C6 | -C6A | -C7 | 118.5(5) |

| | | | | | | | |
|---|---|---|---|---|---|---|---|
| C9 | -C8 | -C7 | 123.3(5) | C6A | -C7 | -C8 | 112.8(4) |
| C6A | -C7 | -C10 | 108.8(5) | C8 | -C7 | -C10 | 112.8(5) |
| C11 | -C10 | -C7 | 111.5(5) | N2 | -C11 | -C10 | 173.9(7) |
| C2 | -C3 | -C3A | 121.3(5) | | | | |

**Table S4.** Final Coordinates and Equivalent Isotropic Displacement Parameters of the non-Hydrogen Atoms for Compound **17**

| Atom | x | y | z | U(eq) [Ang^2] |
|---|---|---|---|---|
| ---- | --- | --- | --- | ---------- |
| K1 | 0.32943(9) | 0.87536(7) | 0.38977(7) | 0.0259(3) |
| O1 | 0.1229(3) | 0.8533(3) | 0.4078(2) | 0.0519(16) |
| N1 | 0.0319(4) | 0.7173(3) | 0.4936(3) | 0.0471(17) |
| N2 | -0.2954(4) | 0.5301(3) | 0.4515(3) | 0.0430(17) |
| C1 | 0.0261(4) | 0.8860(4) | 0.3705(3) | 0.0321(17) |
| C2 | 0.0145(6) | 0.9685(4) | 0.3093(4) | 0.047(2) |
| C3 | -0.0790(7) | 1.0074(4) | 0.2681(4) | 0.052(3) |
| C4 | -0.1758(5) | 0.9684(4) | 0.2801(3) | 0.0440(19) |
| C5 | -0.2768(6) | 1.0119(4) | 0.2386(4) | 0.061(3) |
| C6 | -0.3672(6) | 0.9729(4) | 0.2533(4) | 0.058(3) |

| | | | | |
|---|---|---|---|---|
| C7 | -0.3666(5) | 0.8892(4) | 0.3087(4) | 0.045(2) |
| C8 | -0.2647(5) | 0.8422(4) | 0.3527(4) | 0.0418(19) |
| C9 | -0.2606(5) | 0.7503(4) | 0.4075(4) | 0.0442(19) |
| C10 | -0.1620(5) | 0.7162(3) | 0.4631(3) | 0.0451(19) |
| C11 | -0.0658(5) | 0.7549(3) | 0.4502(3 | 0.0342(17) |

**Table S5.** Anisotropic Displacement Parameters for Compound **17**

| Atom | U(1,1) | U(2,2) | U(3,3) | U(2,3) | U(1,3) | U(1,2) |
|---|---|---|---|---|---|---|
| ---- | ------ | ------ | ------ | ----- | ------ | ------ |
| K1 | 0.0263(6) | 0.0223(5) | 0.0319(6) | -0.0123(5) | 0.0054(5) | -0.0063(4) |
| O1 | 0.026(2) | 0.093(3) | 0.055(3) | -0.045(3) | 0.0066(19) | -0.019(2) |
| N1 | 0.056(3) | 0.047(3) | 0.028(3) | -0.008(2) | -0.008(2) | 0.011(2) |
| N2 | 0.053(3) | 0.028(3) | 0.048(3) | -0.013(2) | 0.002(2) | -0.006(2) |
| C1 | 0.034(3) | 0.042(3) | 0.027(3) | -0.019(3) | 0.010(2) | -0.012(2) |
| C2 | 0.076(5) | 0.042(3) | 0.038(3) | -0.025(3) | 0.024(3) | -0.034(3) |
| C3 | 0.103(6) | 0.020(3) | 0.035(4) | -0.009(3) | 0.017(4) | -0.022(3) |
| C4 | 0.069(4) | 0.032(3) | 0.025(3) | -0.013(3) | -0.026(3) | 0.020(3) |

| | | | | | | |
|---|---|---|---|---|---|---|
| C5 | 0.100(6) | 0.035(4) | 0.046(4) | -0.016(3) | -0.025(4) | 0.005(4) |
| C6 | 0.071(5) | 0.039(4) | 0.064(5) | -0.018(3) | -0.027(4) | 0.002(3) |
| C7 | 0.046(4) | 0.047(4) | 0.051(4) | -0.026(3) | -0.004(3) | -0.012(3) |
| C8 | 0.037(3) | 0.034(3) | 0.059(4) | -0.023(3) | -0.010(3) | 0.000(2) |
| C9 | 0.035(3) | 0.056(4) | 0.041(3) | -0.017(3) | 0.009(3) | -0.007(3) |
| C10 | 0.072(4) | 0.026(3) | 0.034(3) | 0.003(3) | 0.009(3) | -0.025(3) |
| C11 | 0.041(3) | 0.027(3) | 0.025(3) | -0.004(2) | 0.004(2) | 0.008(2) |

**V. Computational Details:**

All the theoretical calculations performed to interpret the experimental observation are performed using Gaussian09 quantum chemistry package[20]. All the Density Functional Theory (DFT) calculations are performed at B3LYP/6-311g+(d) level of theory. Restricted and unrestricted calculations are performed for closed shell and open shell molecules respectively. Solvent effects are considered by optimizing the molecules with Polarized Continuum Model (PCM) using acetonitrile as solvent. Frequency calculations are performed to confirm the optimization to a minimum for all structures. Thermochemistry calculations are obtained from the frequency calculations.

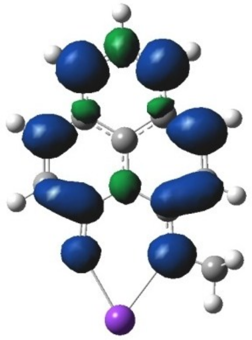

PLY(N,O)-K Radical Anion

PLY(N,O)-K Radical Anion

-1 2

| | | | |
|---|---|---|---|
| 6 | -0.567236211 | -1.484246138 | 0.000258102 |
| 6 | 0.019988139 | -0.143053562 | 0.000168754 |
| 6 | -0.799546103 | 1.094237367 | 0.000138956 |
| 6 | 0.332739043 | -2.594058149 | 0.000109026 |
| 6 | 1.460266794 | -0.029237722 | 0.000056161 |
| 6 | 2.314038838 | -1.193358526 | -0.000033442 |
| 6 | 1.705978803 | -2.466129840 | -0.000017388 |
| 6 | 3.734975744 | -1.053602026 | -0.000134863 |
| 1 | 4.343922076 | -1.954414599 | -0.000188140 |
| 6 | 4.331665950 | 0.201987402 | -0.000162681 |
| 6 | 3.538815458 | 1.346867903 | -0.000121752 |
| 6 | 2.117819575 | 1.258823504 | -0.000039336 |
| 6 | 1.307139059 | 2.411651810 | -0.000056768 |
| 6 | -0.072913258 | 2.331673056 | 0.000027584 |
| 1 | -0.628235769 | 3.261103677 | -0.000089676 |
| 1 | 1.786986378 | 3.389438595 | -0.000162184 |
| 1 | -0.124165156 | -3.581678043 | 0.000146412 |
| 1 | 2.337585352 | -3.353008022 | -0.000104666 |
| 1 | 5.415641613 | 0.290477199 | -0.000225428 |
| 1 | 3.997076918 | 2.333143719 | -0.000167102 |
| 8 | -1.836731585 | -1.734748048 | 0.000493755 |
| 7 | -2.144427173 | 1.065320206 | 0.000202742 |
| 6 | -2.820472773 | 2.346085388 | 0.000209509 |

| | | | |
|---|---|---|---|
| 1 | -2.599588390 | 2.975399573 | -0.882564927 |
| 1 | -3.906760617 | 2.188804893 | 0.000462135 |
| 1 | -2.599194470 | 2.975654213 | 0.882689395 |
| 19 | -4.101998912 | -0.751053739 | -0.000398748 |

*t*BuO Radical

| | | | |
|---|---|---|---|
| 6 | 1.281050000 | -0.785973000 | -0.312893000 |
| 6 | 0.000282000 | -0.031001000 | 0.083884000 |
| 1 | 2.167378000 | -0.212518000 | -0.033027000 |
| 1 | 1.320877000 | -1.753076000 | 0.195005000 |
| 1 | 1.308685000 | -0.967588000 | -1.390279000 |
| 6 | -1.272933000 | -0.798917000 | -0.313182000 |
| 6 | -0.007035000 | 1.388533000 | -0.580193000 |
| 1 | -2.164915000 | -0.234264000 | -0.033502000 |
| 1 | -1.298321000 | -0.980845000 | -1.390567000 |
| 1 | -1.303028000 | -1.766318000 | 0.194803000 |
| 1 | -0.006130000 | 1.245213000 | -1.663259000 |
| 1 | -0.900009000 | 1.945039000 | -0.294344000 |
| 1 | 0.880146000 | 1.954209000 | -0.294219000 |
| 8 | -0.001609000 | 0.266787000 | 1.430462000 |

$CH_3CN$

| | | | |
|---|---|---|---|
| 6 | 0.000000000 | 0.000000000 | -1.175612000 |
| 1 | 0.000000000 | 1.024719000 | -1.550878000 |
| 1 | 0.887433000 | -0.512359000 | -1.550878000 |
| 1 | -0.887433000 | -0.512359000 | -1.550878000 |
| 6 | 0.000000000 | 0.000000000 | 0.279194000 |

| | | | |
|---|---|---|---|
| 7 | 0.000000000 | 0.000000000 | 1.433019000 |

$^t$BuOH

| | | | |
|---|---|---|---|
| 6 | 0.676483000 | 1.263751000 | -0.525141000 |
| 6 | -0.006574000 | -0.000018000 | 0.009523000 |
| 1 | 0.199230000 | 2.159165000 | -0.118802000 |
| 1 | 1.735339000 | 1.284820000 | -0.247313000 |
| 1 | 0.619578000 | 1.310095000 | -1.616273000 |
| 6 | 0.679892000 | -1.261867000 | -0.525280000 |
| 6 | -1.496004000 | -0.001979000 | -0.327421000 |
| 1 | 0.205097000 | -2.158642000 | -0.119071000 |
| 1 | 0.623123000 | -1.308207000 | -1.616418000 |
| 1 | 1.738825000 | -1.280121000 | -0.247511000 |
| 1 | -1.647774000 | -0.001846000 | -1.409379000 |
| 1 | -1.983236000 | -0.888777000 | 0.086186000 |
| 1 | -1.985629000 | 0.883235000 | 0.086742000 |
| 8 | 0.049616000 | 0.000016000 | 1.459517000 |
| 1 | 0.975739000 | 0.000831000 | 1.735615000 |

$CH_2CN$ Radical

| | | | |
|---|---|---|---|
| 6 | 0.000429000 | -1.192376000 | 0.000000000 |
| 1 | 0.000613000 | -1.734859000 | 0.936192000 |
| 1 | 0.000613000 | -1.734859000 | -0.936192000 |
| 6 | 0.000000000 | 0.187917000 | 0.000000000 |
| 7 | -0.000543000 | 1.356639000 | 0.00000000 |

## VI. Reference:

(1) S. H. Cho, J. Y. Kim, S. Y. Lee, and S. Chang, *Angew. Chem. Int. Ed.,* 2009, **48**, 9127 –9130.

(2) F. P. Crisstomo, T. Martin, and R. Carrillo, *Angew. Chem. Int. Ed*., 2014, **53**, 2181–2185.

(3) S. Tani, T. N. Uehara, J. Yamaguchia, and K. Itami, *Chem. Sci.*, 2014, **5**, 123–135.

(4) N. Khatun, S. Guin, S. K. Rout, and B. K. Patel, *RSC Adv.,* 2014, **4**, 10770–10778.

(5) Y. Kawashita, N. Nakamichi, H. Kawabata, and M. Hayashi, *Org. Lett.*, 2003, **5**, 3713-3715.

(6) C. Li, P. Li, J. Yanga, and L. Wang, *Chem. Commun.*, 2012, **48**, 4214–4216.

(7) T. Ertan, I. Yildiz, B. T. Gulbas, K. Bolelli, O. T. Arpaci, S. Ozkan, F. Kaynak, I. Yalcin, and E. Aki, *Eur. J. Med. Chem.*, 2009, **44**, 501-510.

(8) L. Ackermann, S. Barfüsser, and J. Pospech, *Org. Lett.*, 2010, **12**, 724-726.

(9) Y.-X. Chen, L.-F. Qian, W. Zhang, and B. Han, *Angew. Chem. Int. Ed*., 2008, **47**, 9330–9333.

(10) D. Cantillo, C. Mateos, J. A. Rincon, O. de Frutos, and C. O. Kappe, *Chem. Eur. J*., 2015, **21**, 12894-12898.

(11) A. L. Stein, F. N. Bilheri, and G. Zeni, *Chem. Commun.,* 2015, **51**, 15522-15525.

(12) M. D. Perretti, D. M. Monzón, F. P. Crisóstomo, V. S. Martín, and R. Carrillo, *Chem. Commun.*, 2016, **52**, 9036-9039.

(13) D. P. Hari, P. Schroll, and B. König, *J. Am. Chem. Soc*., 2012, **134**, 2958−2961.

(14) X. Wang, X. Ji, C. Shao, Y. Zhang and Y. Zhang, *Org. Biomol. Chem.,* 2017, **15**, 5616-5624.

(15) Q. Xiao, L. Ling, F. Ye, R. Tan, L. Tian, Y. Zhang, Y. Li, J. Wang, *J. Org. Chem.*, 2013, **78**, 3879−3885.

(16) J. S. Ward, J. T. W. Bray, B.J. Aucott, C. Wagner, N. E. Pridmore, A. C. Whitwood, J. W. B. Moir, J. M. Lynam and I. J. S. Fairlamb, *Eur. J. Inorg. Chem*., 2016, 5044–5051.

(17 ) O. V. Dolomanov, L. J. Bourhis, R. J. Gildea, J. A. K. Howard, and H. Puschmann, *J. Appl. Cryst.* 2009, **42**, 339-341.

(18) L. Palatinus, and G. Chapuis, *J. Appl. Cryst.* 2007, **40**, 786-790.

(19) G. M. Sheldrick, *ActaCryst.* 2008, **A64**, 112-122.

(20) Gaussian 09, Rev. D.; M. J. Frisch, G. W. Trucks, H. B. Schlegel, G. E. Scuseria, M. A. Robb, J. R. Cheeseman, G. Scalmani, V. Barone, B. Mennucci, G. A. Petersson, H. Nakatsuji, M. Caricato, X. Li, H. P. Hratchian, A. F. Izmaylov, J. Bloino, G. Zheng, J. L. Sonnenberg, M. Hada, M. Ehara, K. Toyota, R. Fukuda, J. Hasegawa, M. Ishida, T. Nakajima, Y. Honda, O. Kitao, H. Nakai, T. Vreven, J. A. Montgomery, Jr., J. E. Peralta, F. Ogliaro, M. Bearpark, J. J. Heyd, E. Brothers, K. N. Kudin, V. N. Staroverov, R. Kobayashi, J. Normand, K. Raghavachari, A. Rendell, J. C. Burant, S. S. Iyengar, J. Tomasi, M. Cossi, N. Rega, J. M. Millam, M. Klene, J. E. Knox, J. B. Cross, V. Bakken, C. Adamo, J. Jaramillo, R. Gomperts, R. E. Stratmann, O. Yazyev, A. J. Austin, R. Cammi, C. Pomelli, J. W. Ochterski, R. L. Martin, K. Morokuma, V. G. Zakrzewski, G. A. Voth, P. Salvador, J. J. Dannenberg, S. Dapprich, A. D. Daniels, O. Farkas, J. B. Foresman, J. V. Ortiz, J. Cioslowski, and D. J. Fox, Gaussian, Inc., Wallingford CT, 2013.
